# A Systematic Review and New Analyses of the Gender-Equality Paradox

**DOI:** 10.1177/17456916231202685

**Published:** 2024-01-03

**Authors:** Agneta Herlitz, Ida Hönig, Kåre Hedebrant, Martin Asperholm

**Affiliations:** Division of Psychology, Department of Clinical Neuroscience, Karolinska Institutet

**Keywords:** cognition, individual differences, living conditions, personality, sex/gender, sex differences

## Abstract

Some studies show that living conditions, such as economy, gender equality, and education, are associated with the magnitude of psychological sex differences. We systematically and quantitatively reviewed 54 articles and conducted new analyses on 27 meta-analyses and large-scale studies to investigate the association between living conditions and psychological sex differences. We found that sex differences in personality, verbal abilities, episodic memory, and negative emotions are more pronounced in countries with higher living conditions. In contrast, sex differences in sexual behavior, partner preferences, and math are smaller in countries with higher living conditions. We also observed that economic indicators of living conditions, such as gross domestic product, are most sensitive in predicting the magnitude of sex differences. Taken together, results indicate that more sex differences are larger, rather than smaller, in countries with higher living conditions. It should therefore be expected that the magnitude of most psychological sex differences will remain unchanged or become more pronounced with improvements in living conditions, such as economy, gender equality, and education.

Some studies have shown that improved living conditions (e.g., economy, gender equality, education) measured at the country level are associated with larger sex differences^1^ in personality and cognitive functions (e.g., Asperholm, Nagar, et al., 2019; Falk & Hermle, 2018; Giolla & Kajonius, 2019)—sometimes referred to as a “gender-equality paradox” (e.g., Stoet & Geary, 2018). Other studies have reported smaller sex differences in, for example, sexuality (e.g., Schmitt, 2005) or no association in, for instance, spatial skills (e.g., Lauer et al., 2019) in countries with higher^2^ living conditions. Our main aim in this study was to determine whether and, in that case, when variations in living conditions are associated with larger or smaller psychological sex differences. We systematically reviewed previous findings investigating the association between living conditions and sex differences, but we also conducted new analyses on already published large-scale studies and meta-analyses to further investigate the association between living conditions and sex differences.

Systematically investigating factors associated with the magnitude of psychological sex differences is of interest not only because it can contribute with information about causes and explanations but also because it may affect society’s expectations of the effects of societal reforms. Such an investigation might, for example, shape expectations as to whether improvements in gender equality will be associated with women and men becoming more or less similar in their occupational choices or whether higher national gross domestic product (GDP) per capita will be associated with smaller or larger sex differences in aggressive behaviors. Moreover, this may have important policy implications regarding viable means of achieving an even distribution of men and women in different professions, the relevance of sex to educational practices, and other issues.

## Psychological Sex Differences

The existence of sex differences in some psychological dimensions is well documented. For example, women, compared with men, have been reported to have higher academic school grades (measured as a grade point average; Dekhtyar et al., 2018; Voyer & Voyer, 2014), and there is substantial evidence of a female advantage in reading comprehension (Stoet & Geary, 2018) and episodic memory (Asperholm, Högman, et al., 2019; Weber et al., 2014). On the other hand, males typically have an advantage in spatial (Lippa et al., 2010; Voyer et al., 1995) and some numerical tasks (e.g., Weber et al., 2014). In areas of psychological functioning other than cognition, men have been reported to experience fewer depressive symptoms (Salk et al., 2017; Wang et al., 2016), whereas women seem to be less affected by suicide or addictive behaviors (Glenn et al., 2020; Su et al., 2019).

In other instances, there are sex differences that are not necessarily indicative of more or less advantageous performance or behaviors. For example, differences in emotional expression have been reported; females show more internalizing emotions (e.g., sadness) than males, and males display more externalizing emotions (e.g., anger; Chaplin & Aldao, 2013). There are also sex differences when it comes to vocational interest; females prefer to work with people, and males prefer to work with things (Stoet & Geary, 2022; Su et al., 2009). Likewise, several studies have examined personality dimensions and have shown reliable sex differences in which, for instance, females score higher on altruism (Falk & Hermle, 2018) and males score higher on impulsivity (Cross et al., 2011).

Although most of the psychological sex differences are modest in size and the reasons for them insufficiently understood, they are usually reported from early childhood into old age and found in most of the examined regions of the world (e.g., Falk & Hermle, 2018; Weber et al., 2014). The explanations for the sex differences reported in the literature may vary depending on the psychological ability or behavior studied, ranging from being more biologically oriented to more environmentally oriented, but they have typically generated considerable debate. Here, we avoid discussing explanations of the psychological sex differences we examine because our study does not provide causal evidence that can contribute to the explanations of these differences.

## Living Conditions

It may be a truism that the living conditions under which individuals live affect their lives. For example, studies have shown a substantial genetic impact on all psychological traits. Still, that heritability is never estimated to be 100% (it ranges from 30% to 55%) implies that environmental factors also affect the phenotype (Plomin et al., 2016). Another example of how living conditions affect behavior is demonstrated in a natural experiment in which compulsory education was extended from 8 to 9 years, resulting in a 0.75 rise in IQ units (Lager et al., 2017). This increase in IQ units is related to the Flynn effect, which describes the intergenerational gains in IQ scores seen in all regions, often with larger gains in areas that have experienced the fastest development over the examined period. Some living conditions associated with cognitive improvements across generations are increased education, shifts in family size, and improved health care (Pietschnig & Voracek, 2015; Williams, 2013). Collectively, the evidence shows that living conditions can influence psychological functioning in individuals.

There are several composite and more specific country-level indicators of living conditions that can be used to study the environment’s potential effects on psychological dimensions, many of which are publicly available (e.g., World Bank, OECD, UNDP). They focus on several variables, including gender equality, economic opportunities, education, health, and longevity (see Table S1 in the Supplemental Material available online). These indicators are often country-specific because they provide values for countries rather than political or geographical subdivisions. Most are also time-specific,^3^ that is, they provide different values for different years. Some of these indicators are univariate variables, such as women’s share of parliamentary seats and the prevalence of infant mortality. Other indicators are composites of several specific variables, such as the Human Development Index (HDI; UNDP, n.d.) and the Gender Development Index (GDI; UNDP, n.d.). In addition, many indicators of living conditions, both univariate and composite, are correlated. One reason for this is that different composite indexes can be composed of overlapping or similar univariate variables. Another reason is that they are interrelated on a societal level. For instance, economic improvement is typically associated with lifts in education and improvements in female labor-force participation.

## Sex Differences and Living Conditions

The evidence showing that peoples’ living conditions affect their lives (Lager et al., 2017; Pietschnig & Voracek, 2015; Plomin et al., 2016) suggests that living conditions could also affect the magnitude of psychological sex differences. However, some studies have reported that living conditions have little or no impact on the magnitude of sex differences (Lauer et al., 2019; Van de Velde et al., 2013; Zhang et al., 2019). For example, in a previous study (Fellman et al., 2021), we investigated whether the tendency to be more verbally or numerically aligned was influenced by growing up in a gender-typed environment. This was accomplished by examining the entire population of three-sibling families in Sweden, that is, we compared the second-born girl (or boy) growing up with only sisters with the second-born girl (or boy) growing up with only brothers. Despite boys being substantially more numerically aligned and girls being substantially more verbally aligned, there was no evidence supporting the notion that the degree to which one’s tendency to be verbally or numerically aligned at age 16 was affected by growing up in a female- or male-sibling environment.

On the other hand, studies suggest that the extent of the sex differences may differ between countries. This variation may be related to differences in the living conditions of countries. For example, we have reported that for cognitive performance, higher living conditions are associated with larger sex differences in cognitive tasks in which there is an overall female advantage (e.g., episodic memory) and smaller in those in which there is an overall male advantage (e.g., numeracy; Asperholm, Nagar, et al., 2019; Weber et al., 2014). Likewise, Stoet and Geary (2013) reported associations in 15- and 16-year-olds and found smaller sex differences in mathematics performance and larger sex differences in reading comprehension in countries with higher living conditions. For psychological functioning for which sex differences are not indicative of better or worse performance, studies have found that sex differences in personality are more prominent in countries with higher living conditions (Falk & Hermle, 2018; Lippa, 2008). There are also studies that have reported the opposite, that sex differences are smaller in countries with higher living conditions. For example, evidence suggests that sex differences in partner preferences and sexual behavior are smaller in countries with higher living conditions (Barber, 2008; Eagly & Wood, 1999; Lippa, 2009).

## Aims

Because there is no comprehensive analysis of the extent to which living conditions (e.g., indicators of economy, gender equality, education) are associated with the magnitude of psychological sex differences, we investigated this using a three-step approach. First, we reviewed and systematized previous literature in which the association has been assessed (referred to hereafter as “old analyses”). We did this by systematically searching for studies and recording the outcome variable investigated, the indicators of living conditions assessed, and the results of the analyses of the association between these two. Second, to extend these results and reduce possible publication bias, we searched for meta-analyses and large studies on sex differences, made use of the effect sizes collected in these studies, and investigated the associations between the effect sizes and 11 indicators of living conditions (hereafter referred to as “new analyses”). Finally, we summarized the results from the two previous undertakings to determine when variations in living conditions are associated with larger or smaller psychological sex differences.

## Method

Following our first two goals to systemize previous findings (the old analyses) and to complement relevant studies with the necessary data for us to conduct our own analyses (the new analyses), we divided, in large part, subsections of the method description.

### Literature search and article-selection procedure

**Fig. 1.**
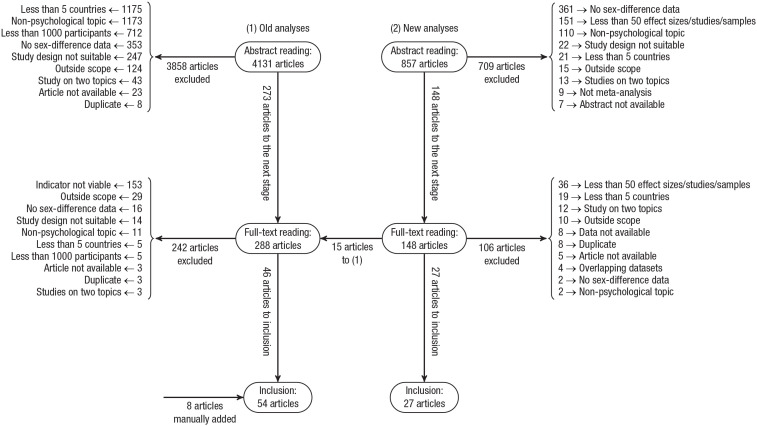
Flowchart of the two article-selection procedures for the old analyses and the new analyses. Explanation of non-self-explanatory exclusion criteria: data not available = sufficient data to conduct new analyses were not available even after trying to contact authors; indicator not viable = the indicators examined were outside the scope of this study (e.g., religiosity); outside scope = the dependent variables investigated were outside the scope of this study (e.g., closeness to kin); overlapping data sets = data were already included from another study; study design not suitable = the study design was not suitable for our investigations (e.g., a systematic review); study on two topics = the dependent variables had to do with a relationship between two variables (e.g., cognitive ability and risk aversion; parental attachment and delinquency).

We conducted two separate literature searches and article-selection procedures for the old analyses and the new analyses (for an overview of the two article-selection procedures, see Fig. 1).

#### Old analyses

Our goal in the literature search for the old analyses was to identify articles in which one or several associations between living conditions and psychological sex differences had been investigated. Thus, in October 2019, a search was performed in the database Web of Science using the search term (“sex* differ*” OR “gender* differ*”) AND (countr* OR nation OR nations OR “*cross nation*” OR “*cross cultur*”) NOT (meta-analy* OR metaanaly*). Only matches in titles, abstracts, and keywords were considered. In addition, only articles in journals likely to publish research relevant to psychology were included. This strategy was achieved by selecting the most relevant categories in the Web of Science journal-classification system.^4^

With these restrictions, the search produced 4,131 abstracts. These abstracts were read to determine their appropriateness for inclusion in the study. Articles that passed this stage were read in full and then either included or excluded from the final study pool. The preliminary selection process was based on five criteria: The study had to (a) be a nonreview scientific report comprising quantitative research on human subjects, (b) examine sex differences, (c) evaluate a clearly delineated measure or behavior broadly construed as psychological, (d) assess more than 1,000 participants, and (e) encompass data from more than five countries. These criteria were applied in the above order. Thus, if an abstract indicated a nonpsychological topic for a study and an insufficient number of participants, the former was registered as the reason for its exclusion. In addition, eight articles that fulfilled our inclusion criteria but did not appear in the search results were manually added.

For the resulting pool of 54 articles, the following general information was registered for each unique combination of a dependent variable and an indicator variable within each sample of each study: country/countries of data collection, number of participants, number of samples, number of data points (and number of studies if applicable), and a basic description of the indicator variable and how it should be interpreted (i.e., whether a higher value of the variable meant higher or lower living conditions). The following information was also registered for the dependent variable: a basic description of it, the effect size in terms of differences between men and women, its type (e.g., Cohen’s *d*, Pearson’s *r*), its significance, and how it should be interpreted (i.e., whether a positive value meant that men or women scored higher or whether such a distinction could not be made, meaning that the effect size indicated a difference but no direction). Finally, the effect size of the test used to investigate the association between the sex difference and the indicator variable and whether this test was performed with single or multiple predictors was registered.

In a few cases in which multiple indicators of living conditions were available, more or less describing the same underlying construct, a subjective selection of relevant indicators was made. Some indicators were not considered, such as population sex ratio and religiosity. In addition, the naming conventions used in the articles were primarily followed for the dependent variables and indicators. However, some adjustments were made to convey when similar underlying constructs were measured or used (e.g., “math” and “math performance”). As for the effect size of association(s), they were noted verbatim as printed (e.g., *b*/beta/β). If the type of effect was unclear, only a number was reported. To the extent specific information was available in the articles, we used that information. However, if specific information was lacking, we used the available information to the best of our ability.

#### New analyses

Our goal was to identify meta-analyses on psychological sex differences. Data were collected from a broad range of locations and time points for which associations between the sex differences and indicators of living conditions had not already been investigated. Thus, in February 2020, a search was performed in the database Web of Science using the search term (“sex* differ*” OR “gender* differ*”) AND (meta-analy* OR metaanaly*). Only matches in titles, abstracts, and keywords were considered. In addition, only articles in journals likely to publish research relevant to psychology were included. This was achieved by selecting the most relevant categories in the Web of Science journal-classification system (for further information, see Note 4).

With these restrictions, the search produced 857 abstracts. These abstracts were read to determine their appropriateness for inclusion in the study. Articles passing this stage were read in their entirety and then either included or excluded from the final study pool. The preliminary selection process was based on six criteria: The study had to (a) be a scientific report comprising quantitative research on human subjects, (b) examine sex differences, (c) evaluate a clearly delineated measure or behavior broadly construed as psychological, (d) be a meta-analysis, (e) include more than 50 studies/samples/effect sizes as presented in the abstract, and (f) encompass data from more than five countries. These criteria were applied in the above order; for example, if an abstract indicated both a nonpsychological topic for a study and an insufficient number of participants, the former was given as the reason for its exclusion.

For the final pool of 27 meta-analyses, the following information was registered: publishing year, country/countries of data collection, the number of male and female participants, the effect sizes regarding differences between men and women, a basic description of the effect size (e.g., Cohen’s *d* or Pearson’s *r*), how it should be interpreted (i.e., whether a positive value meant that men or women scored higher), how it was categorized in terms of broader categories (e.g., Voyer et al., 1995, categorized effect sizes into mental rotation, spatial perception, and spatial visualization), and whether an effect size of 0 was an accurate description of the underlying data or simply a reflection of a nonsignificant result without a specified value.

### Categorization of dependent variables

Some studies investigated several dependent variables. Hence, one study could have variables in several categories and subcategories (e.g., Card et al., 2008). Therefore, to systemize our results and analyze reasonably homogeneous categories of psychological dimensions, we categorized each outcome variable for the old analyses and the new analyses into one of seven categories and, subsequently, to a subcategory of the former. Next, a description of each category, the studies it contains, how data were further subcategorized, and potential difficulties with the collected data are presented (for an overview of the categorizations, see Figs. 4–10).

#### Personal characteristics

Within this category, we have placed dimensions of personality and self-esteem. Personality can be defined as the set of unique and persistent behaviors, cognitions, and emotional patterns characterizing an individual, whereas self-esteem is confidence in one’s abilities or own worth. Some investigators assessed personality through personality inventories, whereas others evaluated specific traits. Mirroring this, we categorized the available studies into Personality-general (Big Five; Costa et al., 2001; Giolla & Kajonius, 2019; Kaiser, 2019; Schmitt et al., 2008), Personality-specific (e.g., risk-taking, narcissism, patience; Cross et al., 2011; Else-Quest et al., 2006; Falk & Hermle, 2018; Grijalva et al., 2015; Lippa, 2008; Miller et al., 2008; Randler & Engelke, 2019; Yang & Girgus, 2019), Self-esteem (Bleidorn et al., 2016; Kling et al., 1999; Zuckerman et al., 2016), and Post-traumatic growth (Vishnevsky et al., 2010).

Sixteen studies investigated sex differences in Personal characteristics: eight in the old analyses (Bleidorn et al., 2016; Costa et al., 2001; Falk & Hermle, 2018; Giolla & Kajonius, 2019; Kaiser, 2019; Lippa, 2008; Schmitt et al., 2008; Zuckerman et al., 2016) and eight in the new analyses (Cross et al., 2011; Else-Quest et al., 2006; Grijalva et al., 2015; Kling et al., 1999; Miller et al., 2008; Randler & Engelke, 2019; Vishnevsky et al., 2010; Yang & Girgus, 2019).

Four studies, all in the old analyses, investigated Personality-general, which constituted sex differences in Big Five inventories (Costa et al., 2001; Giolla & Kajonius, 2019; Kaiser, 2019; Schmitt et al., 2008). Results from these studies show only whether there is a sex difference and not whether one of the sexes scores higher or lower. Note that both Giolla and Kajonius (2019) and Kaiser (2019) used data from International Personality Item Pool (Goldberg et al., 2006); however, because they have used substantially different sample sizes and number of countries, the overlap in analyses appears to be small.

Eight studies investigated Personality-specific, which included more specific personality traits (e.g., altruism, impulsivity, trust). Two of these were in the old analyses (Falk & Hermle, 2018; Lippa, 2008) and six in the new analyses (Cross et al., 2011; Else-Quest et al., 2006; Grijalva et al., 2015; Miller et al., 2008; Randler & Engelke, 2019; Yang & Girgus, 2019).

Self-esteem was examined in a single study in the new analyses (Kling et al., 1999) and in two studies in the old analyses (Bleidorn et al., 2016; Zuckerman et al., 2016). Finally, Post-traumatic growth (resilience after trauma) was examined in a single study in the new analyses (Vishnevsky et al., 2010).

#### Cognition

Cognition can be defined as complex psychological processes active in the acquisition of knowledge and understanding through perception, attention, association, memory, reasoning, judgment, imagination, problem-solving, thought, and language. We subdivided the category Cognition into Math (Dickerson et al., 2015; Else-Quest et al., 2010; Marks, 2008; Stoet & Geary, 2013, 2015; Tao & Michalopoulos, 2018; van Hek et al., 2019; Voyer & Voyer, 2014; Weber et al., 2014), Spatial skills (e.g., line-angle judgment, mental rotation; Lauer et al., 2019; Lippa et al., 2010; Nazareth et al., 2019; Silverman et al., 2007; Voyer et al., 1995, 2017), Verbal abilities (PISA reading performance; Marks, 2008; Stoet & Geary, 2013; van Hek et al., 2019; Voyer & Voyer, 2014), Episodic memory (verbal episodic, object location; Asperholm, Högman, et al., 2019; Silverman et al., 2007; Weber et al., 2014), Autobiographical memory (dream recall; Schredl & Reinhard, 2008), Semantic memory (category fluency; Bonsang et al., 2017; Weber et al., 2014), School performance (Voyer & Voyer, 2014), Duration judgment (R. A. Block et al., 2000), and Science (Stoet & Geary, 2018).

There were 20 studies that investigated sex differences in cognition: 14 in the old analyses (Asperholm, Nagar, et al., 2019; Bonsang et al., 2017; Dickerson et al., 2015; Else-Quest et al., 2010; Lauer et al., 2019; Lippa et al., 2010; Marks, 2008; Silverman et al., 2007; Stoet & Geary, 2013, 2015, 2018; Tao & Michalopoulos, 2018; van Hek et al., 2019; Weber et al., 2014) and six in the new analyses (R. A. Block et al., 2000; Nazareth et al., 2019; Schredl & Reinhard, 2008; Voyer et al., 1995, 2017; Voyer & Voyer, 2014).

Seven studies in the old analyses (Dickerson et al., 2015; Else-Quest et al., 2010; Marks, 2008; Stoet & Geary, 2013, 2015; Tao & Michalopoulos, 2018; van Hek et al., 2019; Weber et al., 2014) and one in the new analyses (Voyer & Voyer, 2014) assessed sex differences in math performance. Six studies in the old analyses used data from PISA (2000–2012) and TIMSS (2003). Thus, there may be duplicate findings reported. However, there is variation in which data wave and how many waves were analyzed, together or apart, and which indicators of living conditions were examined in each study. In Marks (2008), information about the significance of the effects was not reported, but because it was derived from the previously analyzed and well-known PISA database, we assumed that the sex differences and indicator associations were significant (for the latter, if *r* > .20). Only one study investigated math performance in adults (Weber et al., 2014).

Six studies investigated Spatial skills, three in the old analyses (Lauer et al., 2019; Lippa et al., 2010; Silverman et al., 2007) and three in the new analyses (Nazareth et al., 2019; Voyer et al., 1995, 2017). Data in the old analyses came from the BBC study (Lippa et al., 2010; Silverman et al., 2007) and a meta-analysis covering the ages 3 to 18 (Lauer et al., 2019). Both Lippa et al. (2010) and Silverman et al. (2007) used data on mental rotation from the BBC study; Lippa and colleagues seemingly included all countries (*n* = 53) and participants (*n* > 200,000), whereas Silverman and colleagues assessed a subsample of the database. The risk of duplicate reporting is low because they investigated the association between living conditions and sex differences with different living-condition indicators. In addition, for the new analyses, we constructed the data point from Voyer and colleagues (1995) by creating a composite of the three separate measures, mental rotation, spatial visualization, and spatial perception, because they all are facets of spatial skills with similar patterns of sex differences.

Four studies investigated the category Verbal. Three of these were in the old analyses and examined reading performance in the PISA database (2000–2009; Marks, 2008; Stoet & Geary, 2013; van Hek et al., 2019). In the new analyses, scholastic achievement (language school grades; Voyer & Voyer, 2014) was investigated in a single study.

Four studies, all in the old analyses (Asperholm, Nagar, et al., 2019; Bonsang et al., 2017; Silverman et al., 2007; Weber et al., 2014), investigated Episodic memory. Silverman et al. (2007) used data from the BBC study (Reimers, 2007). Both Weber et al., (2014) and Bonsang et al. (2017) used data from SHARE (Survey of Health, Ageing and Retirement in Europe; Börsch-Supan & Jürges, 2005) but assessed associations with different indicators. However, whereas Weber et al. used one wave, Bonsang and colleagues used four waves and included data from four other large databases, making the overlap small. Moreover, although the Asperholm, Nagar, et al. (2019) meta-analysis included data from the same five databases as Bonsang et al., most of the data came from individual studies, resulting in little overlap. Two studies (Bonsang et al., 2017; Weber et al., 2014) that investigated Episodic memory also studied Semantic memory (category “fluency”), again with the same minor overlap in data as described for Episodic memory.

Finally, a number of subcategories were investigated only in single studies: Autobiographical memory (dream recall; Schredl & Reinhard, 2008), Duration judgment (R. A. Block et al., 2000), and School performance (Voyer & Voyer, 2014) were assessed in the new analyses, and Science was assessed in the old analyses (Stoet & Geary, 2018).

#### Interpersonal relations

Interpersonal relations entails behaviors that affect or involve another individual. We subdivided this category into Partner preference (e.g., preference for good looks, domestic skills, earning capacity; Conroy-Beam et al., 2015; Eagly & Wood, 1999; Lippa, 2007; Zentner & Mitura, 2012; Zhang et al., 2019), Sexual behavior (e.g., extramarital sex, frequency of intercourse, sociosexuality, sexting; Barber, 2008; Baumgartner et al., 2014; Lippa, 2009; Petersen & Hyde, 2010; Schmitt, 2005), Aggressive behavior toward others (e.g., frequent fighting, physical aggression, verbal aggression; Archer, 2004; Card et al., 2008; Nivette et al., 2019), Aggressive behavior partners (aggression toward partners; Archer, 2006; Ebbeler et al., 2017), Attachment (anxious attachment, avoidant attachment, dismissing romantic attachment; Del Giudice, 2011; Schmitt, 2008; Schmitt et al., 2003), and Social interaction (cooperation, talkativeness, negotiation performance; Balliet et al., 2011; Leaper & Ayres, 2007; Shan et al., 2019).

Twenty-one studies investigated sex differences in Interpersonal relations: 16 in the old analyses (Archer, 2006; Barber, 2008; Baumgartner et al., 2014; Conroy-Beam et al., 2015; Eagly & Wood, 1999; Ebbeler et al., 2017; Lippa, 2007, 2009; Nivette et al., 2019; Petersen & Hyde, 2010; Schmitt, 2005; Schmitt et al., 2003; Shan et al., 2019; Zentner & Mitura, 2012; Zhang et al., 2019) and five in the new analyses (Archer, 2004; Balliet et al., 2011; Card et al., 2008; Del Giudice, 2011; Leaper & Ayres, 2007).

Five studies, all in the old analyses, investigated Partner preference (Conroy-Beam et al., 2015; Eagly & Wood, 1999; Lippa, 2007; Zentner & Mitura, 2012; Zhang et al., 2019). Data collected by Buss (1989) were analyzed by Eagly and Wood (1999), Conroy-Beam et al. (2015), and Zentner and Mitura (2012). Whereas Eagly and Wood analyzed specific dimensions of Partner preferences (e.g., earning capacity, physical attractiveness), Conroy-Beam and colleagues examined a composite measure, as did Zentner and Mitura. The latter authors also replicated the results in a new data collection. Because there is only partial overlap, the use of the same database should be only mildly problematic.

Five studies, all in the old analyses, investigated Sexual behavior (Barber, 2008; Baumgartner et al., 2014; Lippa, 2009; Petersen & Hyde, 2010; Schmitt, 2005). Barber (2008) and Schmitt (2005) used data from the International Sexuality Description Project (Schmitt et al., 2003). However, Barber limited the investigation to the living-condition indicators GDP and fertility rate, whereas Schmitt investigated these and other indicators.

Two studies in the old analyses (Archer, 2004; Card et al., 2008) and one in the new analyses (Nivette et al., 2019) investigated Aggressive behavior, whereas two studies in the old analyses investigated Aggressive behavior partners (Archer, 2006; Ebbeler et al., 2017).

One study in the new analyses (Del Giudice, 2011) and two in the old analyses (Schmitt, 2008; Schmitt et al., 2003) examined Attachment. Although Schmitt and colleagues (2003, 2008) used the same dependent variable (dismissing romantic attachment) from the International Sexuality Description Project, the indicators of living conditions were mainly different, except for the indicators life expectancy and fertility rate, which were examined in both studies.

Finally, three studies investigated Social interaction: two in the old analyses (Balliet et al., 2011; Leaper & Ayres, 2007) and one in the new analyses (Shan et al., 2019).

#### Emotion

Emotion can be defined as a state of mind associated with thoughts and feelings, intertwined with mood and affect. We subdivided the category Emotion into Negative emotions (e.g., guilt, shame, crying, anger; Archer, 2004; Chaplin & Aldao, 2013; Else-Quest et al., 2012; Fischer et al., 2004; Maes et al., 2019; McDuff et al., 2017; van Hemert et al., 2011), Positive emotions (happiness, smiling; McDuff et al., 2017; Thompson & Voyer, 2014), and Emotion recognition (the process of identifying human emotion; Merten, 2005; Thompson & Voyer, 2014).

Nine studies investigated sex differences in Emotion: four in the old analyses (Ficsher et al., 2004; McDuff et al., 2017; Merten, 2005; van Hemert et al., 2011) and five in the new analyses (Archer, 2004; Chaplin & Aldao, 2013; Else-Quest et al., 2012; Maes et al., 2019; Thompson & Voyer, 2014).

Three studies in the old analyses (Fischer et al., 2004; McDuff et al., 2017; van Hemert et al., 2011) and four studies in the new analyses (Archer, 2004; Chaplin & Aldao, 2013; Else-Quest et al., 2012; Maes et al., 2019) investigated Negative emotions. Positive emotions was investigated in only two studies: one in the old analyses (McDuff et al., 2017) and one in the new analyses (Chaplin & Aldao, 2013). Finally, Emotion recognition was also investigated in only two studies: one in the old analyses (Merten, 2005) and one in the new analyses (Thompson & Voyer, 2014).

#### Mental health

Mental health refers to an individual’s cognitive, emotional, and behavioral well-being. We restricted our examination of potential associations between sex differences in Mental health and living conditions to incorporate the categories Depression (feelings, symptoms, diagnoses; Hopcroft & Bradley, 2007; Hopcroft & McLaughlin, 2012; Salk et al., 2017; Seedat et al., 2009; Van de Velde et al., 2013; Wang et al., 2016), Internet addiction (Su et al., 2019), Nightmare frequency (Schredl & Reinhard, 2011), Problem behavior (Thijs et al., 2015), Life satisfaction (Batz-Barbarich et al., 2018), and Stress appraisal (Davis et al., 1999).

There were 11 studies that investigated sex differences in Mental health: nine in the old analyses (Batz-Barbarich et al., 2018; Hopcroft & Bradley, 2007; Hopcroft & McLaughlin, 2012; Salk et al., 2017; Seedat et al., 2009; Su et al., 2019; Thijs et al., 2015; Van de Velde et al., 2013; Wang et al., 2016) and two in the new analyses (Davis et al., 1999; Schredl & Reinhard, 2011).

Six studies, all in the old analyses, investigated Depression (Hopcroft & Bradley, 2007; Hopcroft & McLaughlin, 2012; Salk et al., 2017; Seedat et al., 2009; Van de Velde et al., 2013; Wang et al., 2016).

All other subcategories were investigated in single studies: Nightmare (frequency; Schredl & Reinhard, 2011) and Stress appraisal (Davis et al., 1999) were assessed in the new analyses, whereas Internet addiction (Su et al., 2019), Problem behavior in adolescents (Thijs et al., 2015), and Life satisfaction (Batz-Barbarich et al., 2018) were assessed in the old analyses.

#### Academic self-concept

Academic self-concept is a construct relating to attitudes, self-efficacy, self-confidence, and motivation toward STEM-oriented school subjects or to academics in general. We subdivided the category academic self-concept into Self-concept STEM (e.g., self-confidence in STEM; Else-Quest et al., 2010; Goldman & Penner, 2016; Huang, 2013; Stoet & Geary, 2018) and Self-concept general academics (self-esteem toward academics in general; Huang, 2013).

Four studies investigated sex differences in Academic self-concept: three in the old analyses (Else-Quest et al., 2010; Goldman & Penner, 2016; Stoet & Geary, 2018) and one in the new analyses (Huang, 2013).

Three studies, all in the old analyses, investigated sex differences in Self-concept STEM (Else-Quest et al., 2010; Goldman & Penner, 2016; Stoet & Geary, 2018). Else-Quest et al. (2010) used data from both PISA and TIMSS from 2003, Goldman and Penner (2016) used TIMSS data from 2007, and Stoet and Geary (2018) used PISA data from 2015. Because data in the studies were derived from different waves, there should be no overlap. Finally, one study in the new analyses (Huang, 2013) investigated sex differences in Self-concept STEM (which we created by combining math and science self-efficacy) and Self-concept general academics.

#### Morals and values

Morals can be defined as the prevailing standards within a group or what society sanctions as correct, whereas values are personal beliefs relating to the judgment of what is important in life. We subdivided Morals and values into Morality (justice- and care-oriented morality; Jaffee & Hyde, 2000) and Values (e.g., marriage defense, hedonism, tradition; Day et al., 2011; Schwartz & Rubel-Lifschitz, 2009).

Three studies investigated sex differences in Morals and values: two in the old analyses (Day et al., 2011; Schwartz & Rubel-Lifschitz, 2009) and one in the new analyses (Jaffee & Hyde, 2000).

Two studies, both in the old analyses (Day et al., 2011; Schwartz & Rubel-Lifschitz, 2009), investigated Values. In Schwartz et al. (2009), each dependent variable (e.g., hedonism, tradition) was analyzed in two separate data sets. A single study in the new analyses investigated Morality (Jaffee & Hyde, 2000).

### Indicators of living conditions

To understand the relationship between variations in sex differences and variations in living conditions, indicators of how living conditions change over time and/or place are needed. Such indicators were already collected and analyzed for the articles assessed in the old analyses, and we added our indicators for the new analyses. More specifically, the indicators collected for the new analyses were country- and time-specific, for example, one value for the United States in 1990, another one for the United States in 1991, and different ones for Sweden in 1990 and 1991.

For ease of interpretation, we divided all the indicators into five overarching categories: Economy, Education, Gender equality, Human development, and Other. Below are descriptions of the indicators included in the old analyses (for an overview of these, see Table S1 in the Supplemental Material) and the new analyses.

#### Old analyses

Several studies analyzed a large number of indicators, some of which were self-constructed composites of specific indicators. In general, we included indicators for which we could determine or infer that they represented higher or lower living conditions. We made several subjective judgments of what constitutes higher living conditions. In most cases, these judgments are unambiguous from a survival perspective (e.g., that more education or access to the health-care system is reflective of higher living conditions). It is less evident in other instances, such as Hofstede’s dimensions (Hofstede et al., 2010). We used our home country (Sweden) as a reference when deciding whether an endpoint of an ambiguous indicator is related to higher or lower living conditions. Thus, higher rather than lower levels of, for instance, individualism (Hofstede et al., 2010) were deemed to constitute higher living conditions. Indicators for which value judgments could not be made (e.g., time) were removed.

##### Economy

Under the category Economy, relatively few (*n* = 4) indicators were used, and the majority used GDP or variants of GDP (gross national income; World Bank, n.d.). We subdivided these indicators into Economy-GDP and Economy-others.

##### Education

The category Education consisted of indicators that referred to, for instance, the enrollment ratio in primary, secondary, and tertiary education (World Bank, n.d.) and literacy ratio (World Bank, n.d.). There were seven indicators in this category, which were not further grouped.

##### Gender equality

Gender equality is the category with the most indicators (*n* = 30), ranging from composite measures, such as the Global Gender Gap Index (GGGI; World Economic Forum, 2021) or the Gender Inequality Index (GGI; UNDP, n.d.) to specific measures, such as women’s share of research positions or share of parliamentary seats (OECD, n.d.; World Bank, n.d.). We decided to categorize indicators explicitly related to the female share/rate of the indicator into the Gender-equality category. However, the gender-neutral version or male version (e.g., labor participation rate) of the same indicators was categorized into Human development or Education. We further subdivided the category Gender equality into GE-composite indicators, GE-culture (e.g., gender role traditionality), GE-economy (e.g., women’s wage equality), GE-education (e.g., female/male ratio in education), GE-life expectancy (e.g., maternal mortality), and GE-representation (e.g., executive positions).

##### Human development

Indicators categorized as assessing Human development were those relating to areas such as disease, demographics, and fertility. There were composite indicators, such as the HDI (UNDP, n.d.) and Regional Development Index (RDI; Weber et al., 2014), and specific indicators, such as teen birth rate, infant mortality, and life expectancy (Gapminder, 2020; World Bank, n.d.). We further subdivided the 16 indicators categorized as assessing human development into HD-composite indicators (HDI, RDI), HD-health (e.g., child malnutrition, infant mortality), HD-fertility (e.g., teen birth rate, contraceptive prevalence), and HD-labor (male labor-participation rate).

##### Other

The category Other includes those indicators that do not fit into the categories Economy, Education, Gender equality, and Human development. Most of these are indicators of Hofstede’s dimensions assessing a society’s cultural values along such dimensions as individualism-collectivism or masculinity-femininity (Hofstede et al., 2010) but also indicators associated with political systems (e.g., level of democracy). The 16 indicators were divided into Other-culture, Other-Hofstede, and Other-miscellaneous.

#### New analyses

In total, 11 indicators were collected. All indicators were calculated so that higher values indicated higher living conditions. The specific indicators used were chosen based on several factors: (a) They should cover a wide range of concepts that capture the overall concept of living conditions, (b) they should mirror the concepts already explored in the old analyses, (c) the underlying data should cover an acceptable range of years and number of countries, and (d) the indicator values between different country–year combinations should have enough variability to be meaningful to analyze. Thus, the indicators should have a specific value for every year (19XX–20XX) and every country, enabling us to map each value to a specific country–year effect size.

##### Gender equality

Four indicators were collected and categorized as belonging to the overarching category Gender equality:

- The Historical Gender Equality Index (Dilli et al., 2019) is a composite measure based on sex ratios on four dimensions: (a) health, (b) autonomy within the household, (c) political power, and (d) socioeconomic status (Dilli et al., 2019). The index comprises 129 countries and ranges from the year 1950 to 2003. The final measure ranges from 0 to 100, “where 100 is a score indicating women have at least achieved an equal position to men on each of the indicators” (Dilli et al., 2019, pp. 42–43).
- Gender Equality in Respect for Civil Liberties (variable v2clgencl in Coppedge et al., 2021) is a composite score measuring to what extent “women enjoy the same level of civil liberties as men” (Coppedge et al., 2021, p. 211); civil liberties here is “understood to include access to justice, private property rights, freedom of movement, and freedom from forced labor” (Coppedge et al., 2021, p. 211). The measure tops out when women enjoy the same level of civil liberties as men and “is similar to a normal (‘Z’) score (e.g. typically between -5 and 5, with 0 approximately representing the mean for all country–years in the sample) though it does not necessarily follow a normal distribution” (Coppedge et al., 2021, p. 31). The index ranges from the year 1900 to 2020.
- Equality in labor-force participation is a measure that indicates the relative participation rate between the sexes in the labor force of people 15 to 64 years of age. More specifically, this measure was created by taking the participation rate of women (0–1) minus the participation rate of men (0–1). These measures were taken from the International Labour Organization’s (2020) ILOSTAT database. The index ranges from the year 1990 to 2019.
- Equality in education is a measure indicating the sex difference in the average years of total schooling of persons 25 years or older. More specifically, this measure was created by taking the average years of schooling for women minus the average years of schooling for men. These measures were taken from Version 2.2 of the Barro-Lee Educational Attainment Dataset (Barro & Lee, 2013). The index ranges from the year 1950 to 2010.

##### Human development

The following four indicators were collected and categorized as belonging to the overarching category Human development:

- RDI is a composite measure of GDP per capita, total fertility rate (representing family size), child-mortality rate, life expectancy, and national education levels. These measures indicate a general-development level, and higher values indicate a higher level of development. The scale is standardized with 0 as the mean value. This measure was initially constructed and used by Weber et al. (2014). The index ranges from the year 1950 to 2018.
- Life expectancy is a measure indicating the number of years newborn infants would live if prevailing patterns of mortality at the time of their birth were the same throughout their lives. The index ranges from the year 1800 to the present. Data were taken from Gapminder (2020; Version 11 of variable GD004).
- Child-mortality rate is a measure indicating the probability of dying between birth and 5 years of age per 1,000 inhabitants. Data were taken from Gapminder (2020; Version 11 of variable GD005). The measure was multiplied by −1 to make higher values indicate lower child mortality. The index ranges from the year 1800 to the present.
- Labor-force participation is a measure indicating the participation rate (0–1) in the labor force of individuals ages 15 to 64 years. Data were taken from the International Labour Organization’s (2020) ILOSTAT database. The index ranges from the year 1990 to 2019.

##### Education

For Education, a single indicator, average years of schooling, was collected. The measure indicates the average years of total schooling of individuals 25 years or older. Data were taken from the Barro-Lee Educational Attainment Dataset (Version 2.2; Barro & Lee, 2013). The index ranges from the year 1950 to 2010.

##### Economy

For Economy, a single indicator, logged GDP per capita, was collected from the 2020 version of the Maddison Project Database (The Maddison Project, 2020). The original measure was logged using the natural logarithm. The index ranges from the year 1950 to 2018.

##### Other

For Other, a single indicator was collected: the Liberal Democracy Index (LDI; variable v2x_libdem in Coppedge et al., 2021). The indicator is a composite measure that “takes a ‘negative’ view of political power insofar as it judges the quality of democracy by the limits placed on government” (Coppedge et al., 2021, p. 44). More specifically, it takes into account “civil liberties, strong rule of law, an independent judiciary, and effective checks and balances that, together, limit the exercise of executive power” (p. 44) and “the level of electoral democracy” (Coppedge et al., 2021, p. 44). The LDI ranges from 0 to 1; higher values indicate more liberal democracies. The index ranges from the year 1800 to 2020.

### Data analysis and interpretation

Although a meta-analytic approach to assess the data would have been beneficial, it was not possible because of the considerable content variation in the dependent variables and within each category and subcategory, making every meaningful division of the data set too small to conduct a meta-analysis. Below is a description of how the data were analyzed in the old analyses and the new analyses, followed by a section on how the results were interpreted.

#### Old analyses

**Fig. 2.**
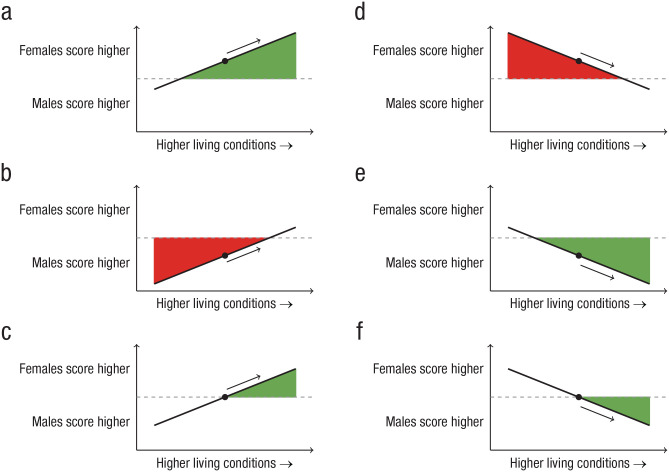
Some scenarios in which the slope describing the linear regression between living conditions and the magnitude of sex differences is always the same. The interpretation of whether the sex differences are larger (green) or smaller (red) in countries with higher living conditions is contingent on the overall magnitude of the mean sex difference (marked by a black dot). (a) Starting from the black dot (indicating that females score higher than males), higher living conditions are associated with a larger sex difference. However, (b) starting from the black dot (indicating that males score higher than females), improvements in living conditions are associated with a smaller sex difference. Moreover, (c) and (f) illustrate the rare occasions in which there are no overall sex differences, but higher living conditions are associated with larger sex differences favoring either (c) females or (f) males.

We determined first, whether the effect size of the sex difference on the dependent variable was statistically significant and second, whether males or females scored higher. If this difference was nonsignificant, we assumed that there were no sex differences. When presenting these original effect sizes in the results, we do not distinguish between different types of standardized mean differences (SMD), such as *d*, *g*, or *z* scores. The term “SMD” is used because it is sometimes unclear exactly which measure was used. Note that this does not affect the final results because it is the direction of the effect (males or females higher) that is used when interpreting the results, rather than the magnitude of the effect. Third, we used the reported indicator analysis (i.e., the association between the effect size and the indicator) to determine whether a sex difference was larger or smaller in countries with higher living conditions, with the overall reported sex difference as a reference point (for more details, see section Interpretation below and Fig. 2). If a significant *p* value was not given for the analysis of the association between the sex difference and the living condition, the magnitude of the sex difference was assumed to be unrelated to living conditions.

#### New analyses

The R package *compute.es* (Del Re, 2013) was used to derive a Cohen’s *d* effect size and a variance of that measure for each data point. All effect sizes were adjusted so that positive values entailed that females scored higher than males. To achieve this, in some of the published meta-analyses and large-scale studies, the effect sizes were multiplied by −1. Whenever it was indicated that an effect size of zero had been imputed in the data set, because the effect size was reported as nonsignificant instead of unknown in the original study, this effect size was removed.

For each outcome variable, a four-level metaregression was performed to estimate the overall sex difference and to conduct indicator analyses. If the overall sex difference was nonsignificant, we assumed that there were no sex differences. These analyses were done using the *rma.mv* function of the *Metafor* package (Viechtbauer, 2010) for R with a random-effects model taking into account the hierarchical structure of studies and samples. The indicator value used was the data point 2 years before the publication of each study included in the meta-analyses because we deemed this value to be closer to when the actual data probably were collected. Whenever there were gaps in the values of the indicator data (e.g., if data were reported in only 5-year-intervals), these values were estimated using linear extrapolation with the nearest available data points. Moreover, if indicator values from before the first or after the last available indicator value were needed, the first or last indicator values were used, respectively, but never more than 5 years away. If it was more than 5 years away, the data point was removed from the indicator analysis.

We used the computed indicator analysis to judge whether a sex difference was larger or smaller in countries with higher living conditions, with the overall computed sex difference as a reference point (for more details, see section Interpretation below and Fig. 2). There were no missing values. We also registered indicator analyses with a *p* value below .15 as possibly smaller or possibly larger (in contrast to the old analyses, in which this information was unavailable).

Note that although the overall sex-difference values were similar to those given in the original meta-analyses, they were not exactly the same. This difference is mainly because we often used a model that differed from the model used in the original meta-analysis and omitted data points with no reported effects.

#### Interpretation

The main concept of importance for the analysis is to what extent sex differences are larger or smaller in countries with higher living conditions. The sex difference indicates whether females or males score higher, whereas the indicator analysis shows whether there is a positive or negative association between the living condition and sex difference. Together, they determine whether the association between the living conditions and the sex difference show larger or smaller sex differences with improvements in living conditions. This is exemplified in Figure 2. For example, if females score higher (Figs. 2a and 2d), a positive linear regression line (Fig. 2a) indicates that with improvements in living conditions, the sex difference is larger in countries with higher living conditions. In contrast, Figure 2d illustrates a scenario in which females score higher but in which improvements in living conditions are associated with smaller sex differences (a negative linear regression line approaching 0). Figures 2b and 2e illustrate scenarios in which males score higher and in which the sex difference is smaller (Fig. 2b) or larger (Fig. 2e) in countries with higher living conditions. Figures 2c and 2f illustrate the rare occasions in which there are no overall sex differences but higher living conditions are associated with larger sex differences favoring either females (Fig. 2c) or males (Fig. 2f).

To summarize and interpret the findings from the old analyses and new analyses, we evaluated the general pattern of findings within each subcategory. Specifically, we determined whether the pattern of data indicated larger (one or several associations), smaller (one or several associations), conflicting (associations were in different directions), or no association (no or occasional associations) between sex differences and living conditions. When interpreting the data for the new analyses, we also considered possibly significant values (i.e., a *p* value below .15) to be able to interpret general but statistically weak patterns.

We cannot make any causality statements from the analyses alone: We can observe only whether there are associations between living conditions and sex differences.

## Results and Discussion

Results on sex differences, associations between the magnitude of sex differences and indicators of living conditions, and subsequent summary of results are presented separately for each category, followed by general analyses of indicators of living conditions. An overview of the results for Personal characteristics, Cognition, Interpersonal relations, Emotion, Mental health, Academic self-concept, and Morals and values appears in Figures 4 through 10.

### Personal characteristics

Within this category, we have placed dimensions of personality and self-esteem. Personality can be defined as the sets of unique and persistent behaviors, cognitions, and emotional patterns characterizing an individual, whereas self-esteem is confidence in one’s abilities or own worth. Some investigators have assessed personality through personality inventories, whereas others have evaluated specific traits. Mirroring this, we categorized the available studies into Personality-general (Big Five), Personality-specific (e.g., risk-taking, patience), Self-esteem, and Post-traumatic growth.

#### Sex differences in personal characteristics

There were reliable sex differences in Personality-general: The magnitude of the difference was Mahalanobis distance = 0.89 to 1.97 (Costa et al., 2001; Falk & Hermle, 2018; Giolla & Kajonius, 2019; Kaiser, 2019; Schmitt et al., 2008). Regarding Personality-specific, we found that females scored higher on agreeableness (SMD = 0.56), altruism (SMD = 0.11), effortful control (SMD = 0.12), extraversion (SMD = 0.15), forgiveness (SMD = 0.28), morningness (SMD = 0.08), neuroticism (SMD = 0.41), positive reciprocity (SMD = 0.06), trust (SMD = 0.06), and sociotropy (SMD = 0.34; Else-Quest et al., 2006; Falk & Hermle, 2018; Lippa, 2008; Miller et al., 2008; Randler & Engelke, 2019; Yang & Girgus, 2019). Females were also higher in Post-traumatic growth (SMD = 0.26; Vishnevsky et al., 2010). Males, on the other hand, were higher on negative reciprocity (SMD = 0.13), patience (SMD = 0.05), risk-taking (SMDs = 0.17–SMD = 0.36), narcissism (SMD = 0.27), and general impulsivity (SMD = 0.09; Cross et al., 2011; Else-Quest et al., 2006; Falk & Hermle, 2018; Grijalva et al., 2015). Negative affectivity was the only univariate personality dimension that showed no sex differences (Else-Quest et al., 2006). For Self-esteem, all studies showed higher levels of general self-esteem for males (SMDs = 0.05–0.25; Bleidorn et al., 2016; Kling et al., 1999; Zuckerman et al., 2016).

#### Living conditions and personal characteristics

The association between living conditions and sex differences in Personality-general in the old analyses showed a relatively homogeneous pattern of larger sex differences in countries with higher living conditions. The two studies in the old analyses (Falk & Hermle, 2018; Lippa, 2008) that investigated the relationship between living conditions and six personality dimensions (altruism, positive reciprocity, trust, negative reciprocity, patience, risk-taking) also indicated that regardless of the direction of the sex difference, they are more prominent in countries with higher living conditions. Likewise, the new analyses revealed that in the univariate personality dimensions in which females score higher (morningness, effortful control, forgiveness, sociotropy), sex differences are larger in countries with higher living conditions. Note that in those personality dimensions in which males score higher (risk-taking, narcissism, general impulsivity, surgency factor), the new analyses showed less systematic effects.

For Self-esteem, the old analyses indicated larger sex differences in self-esteem in countries with higher living conditions. By contrast, the one study in the new analyses showed no systematic effect. We found no association between living conditions and sex differences in Post-traumatic growth.

**Fig. 3.**
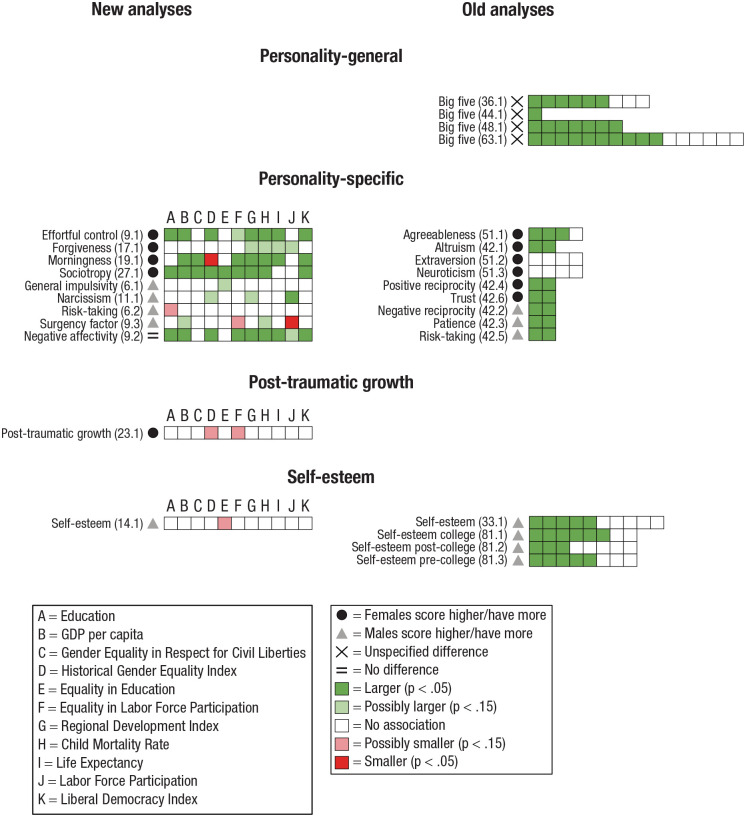
Results for each subcategory of Personality characteristics. Significant overall sex differences are indicated with symbols (see legend at right). Each square denotes a tested association between a dependent variable, listed to the left of the square, and an indicator of a living condition (see left side of legend for indicators used in new analyses). A dark green square indicates a significant (*p* < .05) association in which higher living conditions are associated with larger sex differences. A light green square indicates a marginally significant (*p* < .15) association of the same sort (applicable only for new analyses). A dark red square indicates a significant (*p* < .05) association in which higher living conditions are associated with smaller sex differences. A light red square indicates a marginally significant (*p* < .15) association of the same sort (applicable only for new analyses). A white square indicates a nonsignificant association between living conditions and sex differences (*p* > .05 for the old analyses; *p* > .15 for the new analyses). Numbers within parentheses (x.y) indicate study (x) and dependent variable (y) analyzed, with corresponding numbers given in the data tables (see the column Dependent Variable Number in Table S2 in the Supplemental Material). Studies: (6) Cross et al. (2011), (9) Else-Quest et al. (2006), (11) Grijalva et al. (2015), (14) Kling et al. (1999), (17) Miller et al. (2008), (19) Randler and Engelke (2019), (23) Vishnevsky et al. (2010), (27) Yang and Girgus (2019), (33) Bleidorn et al. (2016), (36) Costa et al. (2001), (42) Falk and Hermle (2018), (44) Giolla and Kajonius (2019), (48) Kaiser (2019), (51) Lippa (2008), (63) Schmitt et al. (2008), (81) Zuckerman et al. (2016).

For an overview of the results for the category personal characteristics, see Figure 3.

#### Summary of personal characteristics

The general pattern indicates that sex differences in personality characteristics are larger in countries with higher living conditions. There is somewhat weaker support for larger sex differences in countries with higher living conditions in some of the personality dimensions in which males scored higher (risk-taking, surgency). Note that the consistency in results is more robust in the old analyses than in the new analyses.

### Cognition

Cognition can be defined as complex psychological processes active in the acquisition of knowledge and understanding through perception, attention, association, memory, reasoning, judgment, imagination, problem-solving, thought, and language. We subdivided the category cognition into Math, Spatial skills, Verbal abilities (PISA reading performance), Episodic memory (verbal episodic, object location), Autobiographical memory (dream recall), Semantic memory (category fluency), School performance, Duration judgment, and Science.

#### Sex differences in cognition

In general, males performed at a higher level in Math, and this holds true for both young and old (Dickerson et al., 2015; Else-Quest et al., 2010; Marks, 2008; Stoet & Geary, 2013, 2015; Tao & Michalopoulos, 2018; van Hek et al., 2017; Weber et al., 2014), although sex differences are lacking in data from TIMSS (Else-Quest et al., 2010). Voyer and Voyer (2014) investigated school grades in mathematics and found a female advantage, as is typically the case for school grades (SMD = 0.15).

Regarding the category Spatial, all studies from the old analyses and new analyses found sex differences favoring males and had relatively homogeneous effect sizes from SMD = −0.39 to SMD = −0.49 (Lauer et al., 2019; Lippa et al., 2010; Silverman et al., 2007) and from SMD = −0.23 to SMD = −0.39 (Nazareth et al., 2019; Voyer et al., 1995, 2017), respectively.

For Episodic memory, there is a female advantage regardless of the type of memory task (verbal episodic, immediate and delayed word recall, object location); effect sizes ranged from SMD = 0.23 to SMD = 0.31 (Asperholm, Högman, et al., 2019; Bonsang et al., 2017; Silverman et al., 2007; Weber et al., 2014). A female advantage is also seen for Autobiographical memory (SMD = 0.23; Schredl & Reinhard, 2008). Likewise, all studies that investigated Verbal abilities showed an advantage for females (SMDs = 0.32–0.41), although specific effect sizes were not always reported. By contrast, two studies that investigated Semantic memory (category fluency) indicated that there is a male advantage (Bonsang et al., 2017; Weber et al., 2014). Finally, School performance showed a female advantage (Voyer & Voyer, 2014), whereas no sex differences could be found for Duration judgment (R. A. Block et al., 2000) or Science (Stoet & Geary, 2018).

#### Living conditions and cognition

**Fig. 4.**
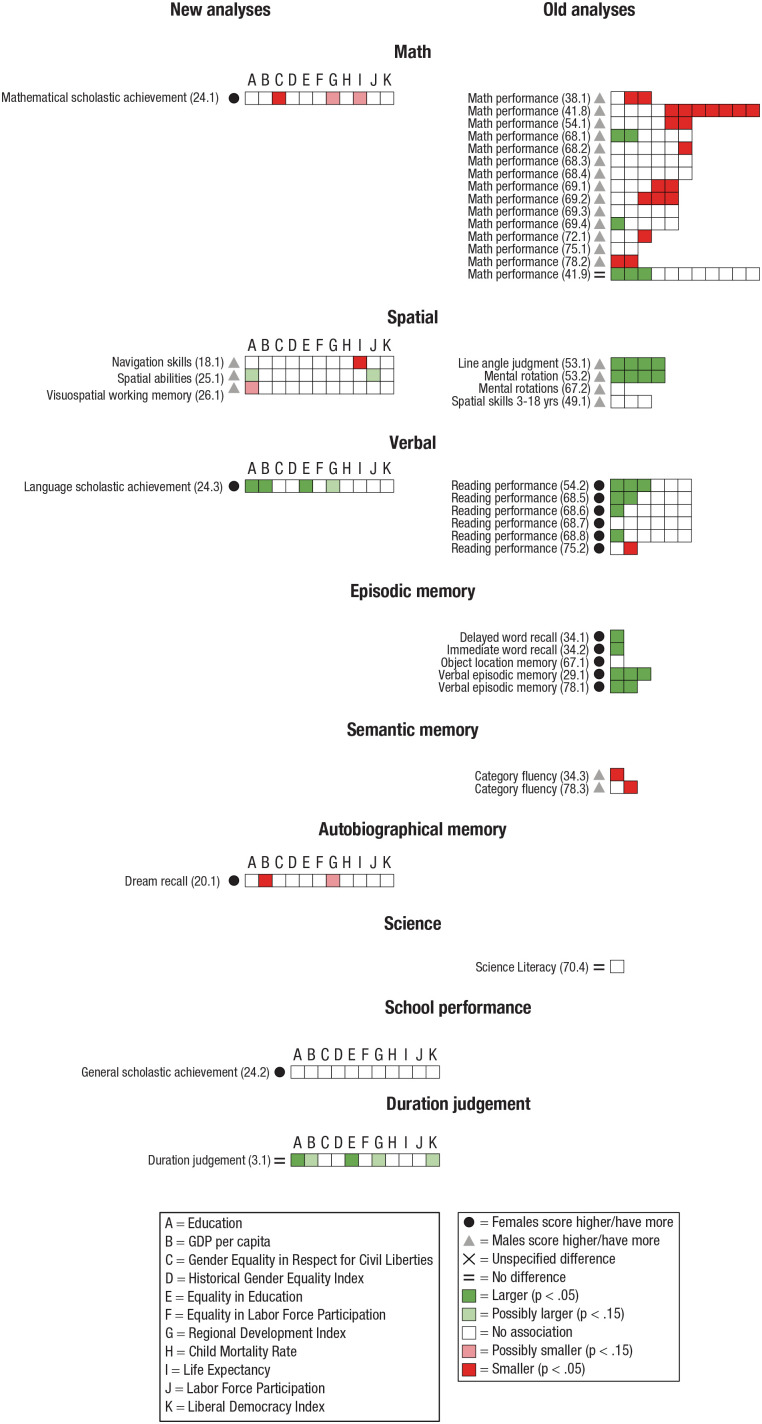
Results for each subcategory of Cognition. Significant overall sex differences are indicated with symbols (see legend on right). Each square denotes a tested association between a dependent variable, listed to the left of the square, and an indicator of a living condition (see left side of legend for indicators used in new analyses). A dark green square indicates a significant (*p* < .05) association in which higher living conditions are associated with larger sex differences. A light green square indicates a marginally significant (*p* < .15) association of the same sort (applicable only for new analyses). A dark red square indicates a significant (*p* < .05) association in which higher living conditions are associated with smaller sex differences. A light red square indicates a marginally significant (*p* < .15) association of the same sort (applicable only for new analyses). A white square indicates a nonsignificant association between living conditions and sex differences (*p* > .05 for the old analyses; *p* > .15 for the new analyses). Numbers within parentheses (x.y) indicate study (x) and dependent variable (y) analyzed, with corresponding numbers given in the data tables (see the column Dependent Variable Number in Table S2 in the Supplemental Material). Studies: (3) R. A. Block et al. (2000); (18) Nazareth et al. (2019); (20) Schredl and Reinhard (2008); (24) Voyer and Voyer (2014); (25) Voyer et al. (1995); (26) Voyer et al. (2017); (29) Asperholm, Nagar, et al. (2019); (34) Bonsang et al. (2017); (38) Dickerson et al. (2015); (41) Else-Quest et al. (2010); (49) Lauer et al. (2019); (53) Lippa et al. (2010); (54) Marks (2008); (67) Silverman et al. (2007); (68) Stoet and Geary (2013); (69) Stoet and Geary (2015); (70) Stoet and Geary (2018); (72) Tao and Michalopoulos (2018); (75) van Hek et al. (2019); (78) Weber et al. (2014).

For Math, the old analyses indicated that the male advantage is smaller in countries in which living conditions are higher. At first sight, it might appear that the results of Else-Quest et al. (2010) run contrary to this pattern because the sex differences are larger in countries in which living conditions are higher (see Fig. 4). However, as exemplified in Figure 2, the same underlying slope can yield larger differences in one case (e.g., Fig. 2c, as in Else-Quest et al., 2010) and smaller differences in another (e.g., Fig. 2b, as in Marks, 2008). Hence, the larger effect in Else-Quest et al. (2010) indicates that the nonsignificant overall sex difference becomes a female advantage in countries with higher living conditions. Two studies showed some effects in the opposite direction, that is, that sex differences are larger in countries with higher living conditions (Stoet & Geary, 2013, 2015). The new analyses, based on a single study that investigated school grades in mathematics, indicated that sex differences are smaller in countries with higher living conditions (Voyer & Voyer, 2014). Note that studies that used data on older adults (55+; Dickerson et al., 2015; Voyer & Voyer, 2014; Weber et al., 2014) found a rather coherent pattern of sex differences being smaller in countries with higher living conditions. These results lend support to the notion that the male advantage in mathematics is smaller in countries with higher living conditions.

Regarding Spatial skills, one of the three studies in the old analyses found that sex differences are larger in countries with higher living conditions (Lippa et al., 2010), whereas two other studies found no association (Lauer et al., 2019; Silverman et al., 2007). For the new analyses, there were no systematic effects pointing in any direction among the three studies investigated (Nazareth et al., 2019; Voyer et al., 1995, 2017).

For Verbal, the old analyses suggested that sex differences favoring females are larger in countries with higher living conditions (Marks, 2008; Stoet & Geary, 2013), although one study showed a contradictory result (van Hek et al., 2019). The pattern indicating larger sex differences in countries with higher living conditions is strengthened by the same pattern found in the new analyses (Voyer & Voyer, 2014).

For Episodic memory, sex differences favoring women appear to be larger in countries with higher living conditions (Asperholm, Nagar, et al., 2019; Bonsang et al., 2017; Weber et al., 2017), although one study that examined object-location memory did not find such an association (Silverman et al., 2007). For Semantic memory (category fluency), analyzed in the old analyses, both studies indicated that the male advantage is smaller in countries with higher living conditions (Bonsang et al., 2017; Weber et al., 2014).

Neither the single study in the Science category (Stoet & Geary, 2018) nor the single study that assessed School performance (Voyer & Voyer, 2014) showed any association between sex differences and living conditions. However, the single study for Duration judgment showed that the female advantage was larger in countries with higher living conditions.

For an overview of the results for the category Cognition, see Figure 4.

#### Summary of cognition

Some evidence suggests that females gain cognitively more than males in countries with higher living conditions such that the male advantage in math and semantic memory is smaller in countries with higher living conditions. In line with this notion, the female advantage in verbal episodic memory and verbal abilities is larger in countries with higher living conditions. However, the same pattern was not found for scholastic achievement in mathematics (indeed, the opposite) or spatial skills.

### Interpersonal relations

Interpersonal relations entails behaviors that affect or involve another individual. We subdivided this category into Partner preference (e.g., preference for good looks, domestic skills, earning capacity), Sexual behavior (e.g., extramarital sex, frequency of intercourse, sociosexuality, sexting), Aggressive behavior toward others (e.g., frequent fighting, physical aggression, verbal aggression), Aggressive behavior partners (aggression toward partners), Attachment (e.g., anxious attachment, avoidant attachment), and Social interaction (cooperation, talkativeness, negotiation performance).

#### Sex differences in interpersonal relations

Regarding partner preferences, men and women tend to value somewhat different capacities in their partners (*r*s = .48–.84; Buss & Schmitt, 2019; Conroy-Beam et al., 2015; Zentner & Mitura, 2012). Although women value good earning capacity (Eagly & Wood, 1999; Zhang et al., 2019) and niceness (SMD = 0.20; Lippa, 2007) to a higher extent than men do, men value physical attractiveness (SMD = 0.55; Eagly & Wood, 1999; Lippa, 2007; Zhang et al., 2019) and intelligence (SMD = 0.10; Lippa, 2007) more than women do. No sex difference was seen for preference for domestic skills (Zhang et al., 2019).

There are sex differences indicating that men score higher on measures of Sexual behavior (e.g., extramarital sex, number of partners, pornography consumption, sex drive, sociosexuality; SMDs = 0.06–0.74; Barber, 2008; Lippa, 2009; Petersen & Hyde, 2010; Schmitt, 2005). Moreover, men dismiss or avoid romantic attachment more than women do (SMDs = 0.12–0.18; Del Giudice, 2011; Schmitt, 2008; Schmitt et al., 2003), whereas anxious attachment is higher in women (SMD = 0.08; Del Giudice, 2011). Women engage more in same-gender sex (SMD = 0.05; Petersen & Hyde, 2010), and there are no sex differences in sexting (Baumgartner et al., 2014).

Both the old analyses and the new analyses indicated that there are sex differences in Aggressive behavior toward others; there are higher levels for men in almost all types of aggression (e.g., physical, verbal; odds ratio = 2.68, SMDs = 0.29–0.62; Archer, 2004; Card et al., 2008; Nivette et al., 2019). Men also display higher levels of Aggressive behavior partners (SMDs = 0.04–9.075; Archer, 2006; Ebbeler et al., 2017). Women, on the other hand, display higher levels of indirect aggression (SMD = 0.13; Archer, 2004) or a tendency toward higher levels (SMD = 0.072; Card et al., 2008).

Regarding Social interactions, men display higher levels of talkativeness (SMD = 0.21; Leaper & Ayres, 2007), negotiating performance (SMD = 0.15; Shan et al., 2019), and cooperation for same-sex interactions in social dilemmas (SMD = 0.17; Balliet et al., 2011). Women are more likely to cooperate in mixed-sex interactions (SMD = 0.24; Balliet et al., 2011).

#### Living conditions and interpersonal relations

The association between living conditions and sex differences in Sexual behavior shows a rather consistent pattern with most previous data indicating that sex differences in Sexual behavior are smaller in countries with higher living conditions (Barber, 2008; Lippa, 2009; Petersen & Hyde, 2010; Schmitt, 2005). For sexting (Baumgartner et al., 2014), in which no sex differences are seen in the average level, the sex difference is larger in countries with higher living conditions. This pattern indicates that sexting is more common among males in more traditional countries and more common among females in less traditional countries (for an illustration, see Fig. 2c). The pattern seen for Sexual behavior is also found for Partner preference such that sex differences, regardless of direction, are smaller in countries with higher living conditions.

In the new analyses for Aggressive behavior, sex differences seem to be larger in countries with higher living conditions for overall aggression, for which males score higher, and also for indirect aggression, for which females score higher or no difference can be found (Archer, 2004; Card et al., 2008). This pattern is also present for Aggressive behavior (frequency of fighting) in the old analyses, which found that men engage more in frequent fighting in countries with higher living conditions (Nivette et al., 2019). The opposite pattern is seen for Aggressive behavior partners, indicating that the sex difference (i.e., men display higher levels of physical aggression toward their partners) is smaller in countries with higher living conditions, as reported in two studies in the old analyses (Archer, 2006; Ebbeler et al., 2017).

Regarding Attachment, two studies in the old analyses (Schmitt, 2008; Schmitt et al., 2003), likely using the same data with different indicators of living conditions, convincingly indicate that the sex differences (males higher) in attachment (dismissing romantic attachment) are larger in countries with higher living conditions. A weaker but similar pattern was found for avoidant attachment in the new analyses (Del Giudice, 2011). For Attachment, for which women score higher (anxious attachment; Del Giudice, 2011), there is an ambiguous pattern with effects indicating both a larger and a smaller sex difference in countries with higher living conditions (Del Giudice, 2011).

Regarding Social interactions, there seems to be no association between sex differences and living conditions, although one effect in negotiation performance indicates larger sex differences favoring men in countries with higher living conditions (Shan et al., 2019). In the new analyses, there are, however, no associations between living conditions and sex differences in talkativeness or cooperation in social dilemmas (Balliet et al., 2011; Leaper & Ayres, 2007).

**Fig. 5.**
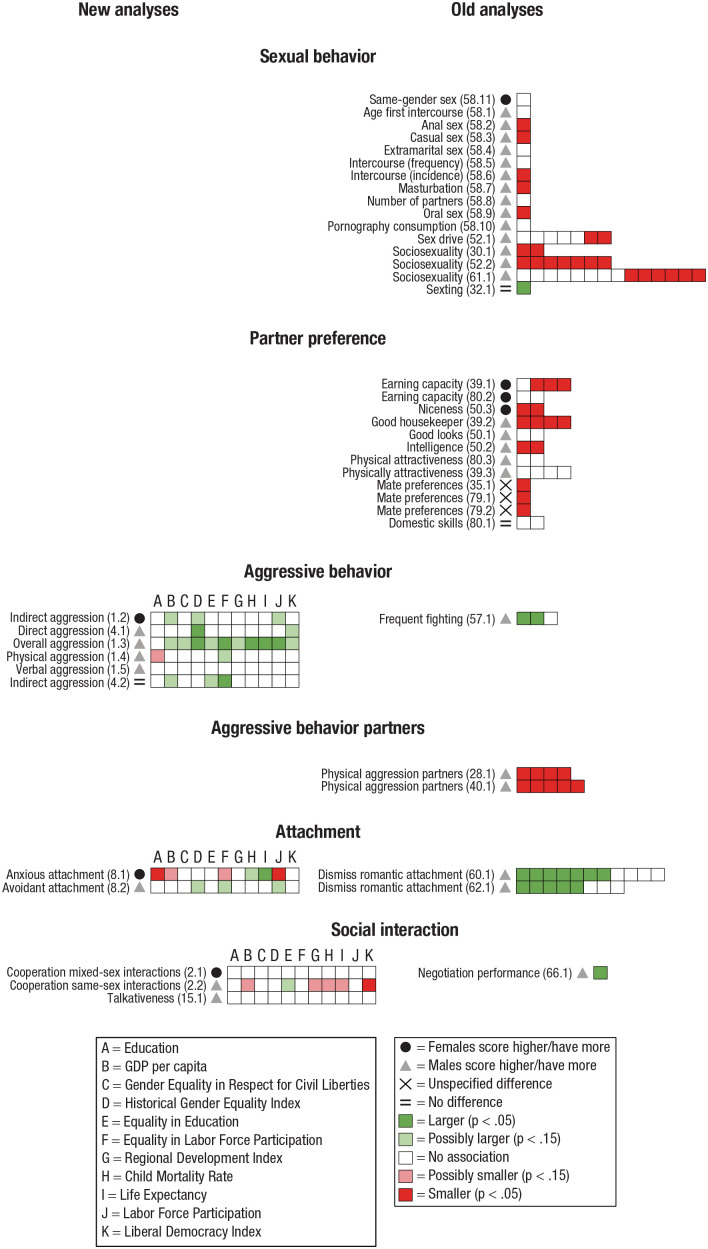
Results for each subcategory of Interpersonal relations. Significant overall sex differences are indicated with symbols (see right side legend). Each square denotes a tested association between a dependent variable, listed to the left of the square, and an indicator of a living condition (see left side of legend for indicators used in new analyses). A dark green square indicates a significant (*p* < .05) association, in which higher living conditions are associated with larger sex differences. A light green square indicates a marginally significant (*p* < .15) association of the same sort (applicable only for new analyses). A dark red square indicates a significant (*p* < .05) association, in which higher living conditions are associated with smaller sex differences. A light red square indicates a marginally significant (*p* < .15) association of the same sort (applicable only for new analyses). A white square indicates a nonsignificant association between living conditions and sex differences (*p* > .05 for the old analyses; *p* > .15 for the new analyses). Numbers within parentheses (x.y) indicate study (x) and dependent variable (y) analyzed, with corresponding numbers given in the data tables (see the column Dependent Variable Number in Table S2 in the Supplemental Material). Studies: (1) Archer (2004), (2) Balliet et al. (2011), (4) Card et al. (2008), (8) Del Giudice (2011), (15) Leaper and Ayres (2007), (28) Archer (2006), (30) Barber (2008), (32) Baumgartner et al. (2014), (35) Conroy-Beam et al. (2015), (39) Eagly and Wood (1999), (40) Ebbeler et al. (2017), (50) Lippa (2007), (52) Lippa (2009), (57) Nivette et al. (2019), (58) Petersen and Hyde (2010), (60) Schmitt (2008), (61) Schmitt (2005), (62) Schmitt et al. (2003), (66) Shan et al. (2019), (79) Zentner and Mitura (2012), (80) Zhang et al. (2019).

For an overview of the results for the category interpersonal relations, see Figure 5.

#### Summary of interpersonal behavior

On the basis of studies from the old analyses, we found a consistent pattern showing that sex differences in sexual behavior and partner preferences are smaller in countries with higher living conditions. For Aggressive behavior partners, we found a clear and consistent pattern with smaller sex differences in countries with higher living conditions. By contrast, for Aggressive behavior, the analyses indicated that there were larger sex differences in countries with higher living conditions regardless of whether males or females displayed higher levels. Concerning attachment, the pattern is less straightforward.

### Emotion

Emotion can be defined as a state of mind associated with thoughts and feelings, intertwined with mood and affect. We subdivided the category Emotion into Negative emotions (e.g., guilt, shame, crying, anger), Positive emotions (happiness, smiling), and Emotion recognition.

#### Sex differences in emotion

Regarding Negative emotions, females reported higher levels of internalizing emotions, such as guilt, shame, crying, and fear (SMDs = 0.12–1.11; Chaplin & Aldao, 2013; Else-Quest et al., 2010; Fischer et al., 2004; van Hemert et al., 2011), whereas males had higher levels of externalizing anger, antagonism, brow furrowing, and loneliness (SMDs = 0.07–0.11; Chaplin & Aldao, 2013; Fischer et al., 2004; Maes et al., 2019; McDuff et al., 2017). However, there were also studies that indicated no sex differences regarding Negative emotions (self-reported anger, negative emotion, powerful emotions; Archer, 2004; Chaplin & Aldao, 2013; Fischer et al., 2004). For Positive emotions, women have higher levels of happiness and smile more (SMD = 0.10; Chaplin & Aldao, 2013; McDuff et al., 2017).

Considering the results from both the old analyses and new analyses together, there were sex differences indicating that females relative to males are more proficient in recognizing emotions (SMDs = 0.22–0.30; Merten, 2005; Thompson & Voyer, 2014).

#### Living conditions and emotion

For those cases in which there is an overall sex difference such that females score higher on Negative emotions, higher living conditions seem to predict that they express and feel even more negative emotions (e.g., guilt and crying) compared with men (Archer, 2004; Chaplin & Aldao, 2013; Else-Quest et al., 2012; Fischer et al., 2004; Maes et al., 2019; McDuff et al., 2017; van Hemert et al., 2011). However, no clear pattern can be seen for measures for which males express more negative emotions; regarding loneliness and antagonism, sex differences seem to be smaller in countries with higher living conditions (Fischer et al., 2004; Maes et al., 2019). On the other hand, if anything, an opposite pattern seems to be present for self-reported anger and brow furrowing, that is, there are larger sex differences in countries with higher living conditions (Chaplin & Aldao, 2013; McDuff et al., 2017). Finally, no clear pattern can be seen for measures for which there is no sex difference (Archer, 2004; Chaplin & Aldao, 2013; Fischer et al., 2004).

For Positive emotions, the single study in the old analyses that investigated smiling (McDuff et al., 2017) showed no effect of living conditions. However, for the single study analyzed in the new analyses, in which females scored higher (Chaplin & Aldao, 2013), sex differences were smaller in countries with higher living conditions.

Finally, for Emotion recognition, the studies in the old analyses and new analyses (Merten, 2005; Thompson & Voyer, 2014) indicated that the sex differences favoring females are larger in countries with higher living conditions, but the evidence is weak.

**Fig. 6.**
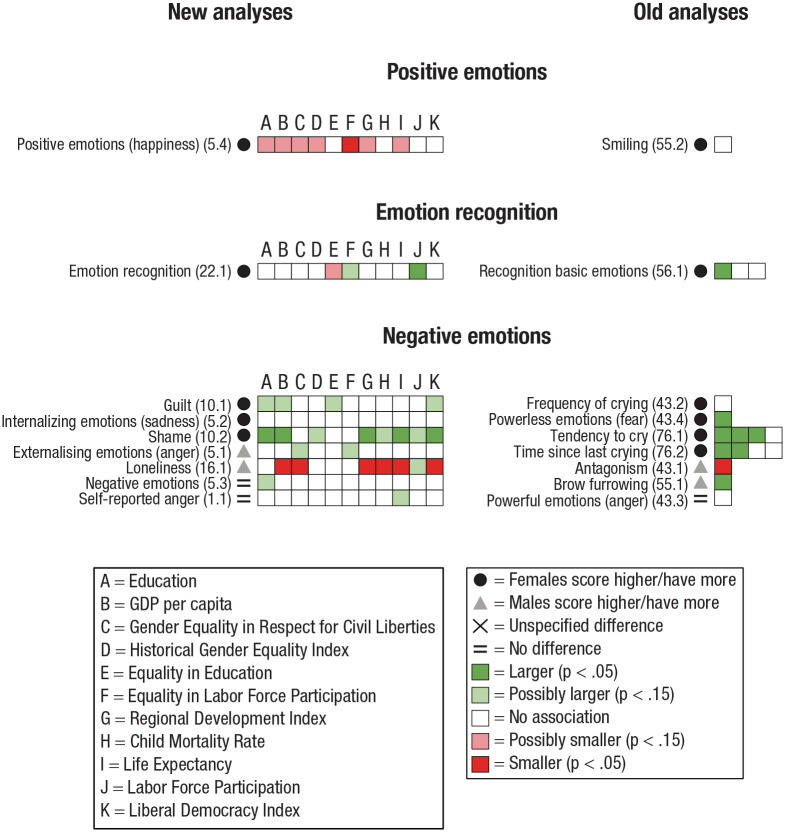
Results for each subcategory of Emotions. Significant overall sex differences are indicated with symbols (see right side of legend). Each square denotes a tested association between a dependent variable, listed to the left of the square, and an indicator of a living condition (see left side of legend for indicators used in new analyses). A dark green square indicates a significant (*p* < .05) association, in which higher living conditions are associated with larger sex differences. A light green square indicates a marginally significant (*p* < .15) association of the same sort (applicable only for new analyses). A dark red square indicates a significant (*p* < .05) association, in which higher living conditions are associated with smaller sex differences. A light red square indicates a marginally significant (*p* < .15) association of the same sort (applicable only for new analyses). A white square indicates a nonsignificant association between living conditions and sex differences (*p* > .05 for the old analyses; *p* > .15 for the new analyses). Numbers within parentheses (x.y) indicate study (x) and dependent variable (y) analyzed, with corresponding numbers given in the data tables (see the column Dependent Variable Number in Table S2 in the Supplemental Material). Studies: (1) Archer (2004), (5) Chaplin and Aldao (2013), (10) Else-Quest et al. (2012), (16) Maes et al. (2019), (22) Thompson and Voyer (2014), (43) Fischer et al. (2004), (55) McDuff et al. (2017), (56) Merten (2005), (76) van Hemert et al. (2011).

For an overview of the results for the category Emotion, see Figure 6.

#### Summary of emotion

Sex differences in emotions show that some are more prevalent or stronger in females, whereas others are stronger or more prevalent in men. Results indicate that those sex differences in negative emotions that are more prevalent or stronger in females are larger in countries with higher living conditions. By contrast, but much less systematically, there are some indications that those sex differences in negative emotions that are more prevalent or stronger in males are smaller in countries with higher living conditions. Note that one study from the new analyses suggests that the sex difference in positive emotions that is more prevalent or stronger in females is smaller in countries with higher living conditions. Cautiously, these results could be interpreted as if higher living conditions are associated with more adverse effects on emotions in females than in males.

### Mental health

Mental health refers to an individual’s cognitive, emotional, and behavioral well-being. We restricted our examination of potential associations between sex differences in Mental health and living conditions to incorporate the categories Depression (feelings, symptoms, diagnoses), Internet addiction, Nightmare frequency, Problem behavior, Life satisfaction, and Stress appraisal.

#### Sex differences in mental health

As has often been reported, females have in most studies been found to have higher rates of Depression (SMDs = 0.19–0.38; Hopcroft & Bradley, 2007; Hopcroft & McLaughlin, 2012; Salk et al., 2017; Seedat et al., 2009; Van de Velde et al., 2013; Wang et al., 2016). By contrast, males have higher levels of Internet addiction (SMD = 0.15; Su et al., 2019) and Problem behavior (Thijs et al., 2015). The new analyses indicated that females report higher levels of Nightmare frequency (SMD = 0.18; Schredl & Reinhard, 2011) and Stress appraisal (SMD = 0.24; Davis et al., 1999). Finally, there are no apparent sex differences in Life satisfaction (Batz-Barbarich et al., 2018).

#### Living conditions and mental health

Considering studies from only the old analyses, we found that females have more symptoms, diagnoses, or feelings of depression in countries with higher living conditions (Hopcroft & Bradley, 2007; Hopcroft & McLaughlin, 2012; Salk et al., 2017; Seedat et al., 2009; Van de Velde et al., 2013; Wang et al., 2016). Note, however, that one study showed a single association in which the sex difference in depression diagnosis, instead, is smaller in countries with higher living conditions (Seedat et al., 2009).

In the remaining categories, there is only one study per category. With that said, the single study in the old analyses that investigated associations between sex differences in Internet addiction and living conditions reported that when living conditions are higher, the sex difference is smaller (Su et al., 2019). For Nightmare frequency, the new analyses seemed to show a somewhat consistent pattern (Schredl & Reinhard, 2011) indicating that females remember more nightmares in countries with higher living conditions. No consistent pattern is seen between living conditions and sex differences in Problem behavior in adolescence (Thijs et al., 2015), Life satisfaction (Batz-Barbarich et al., 2018), or Stress appraisal (Davis et al., 1999).

**Fig. 7.**
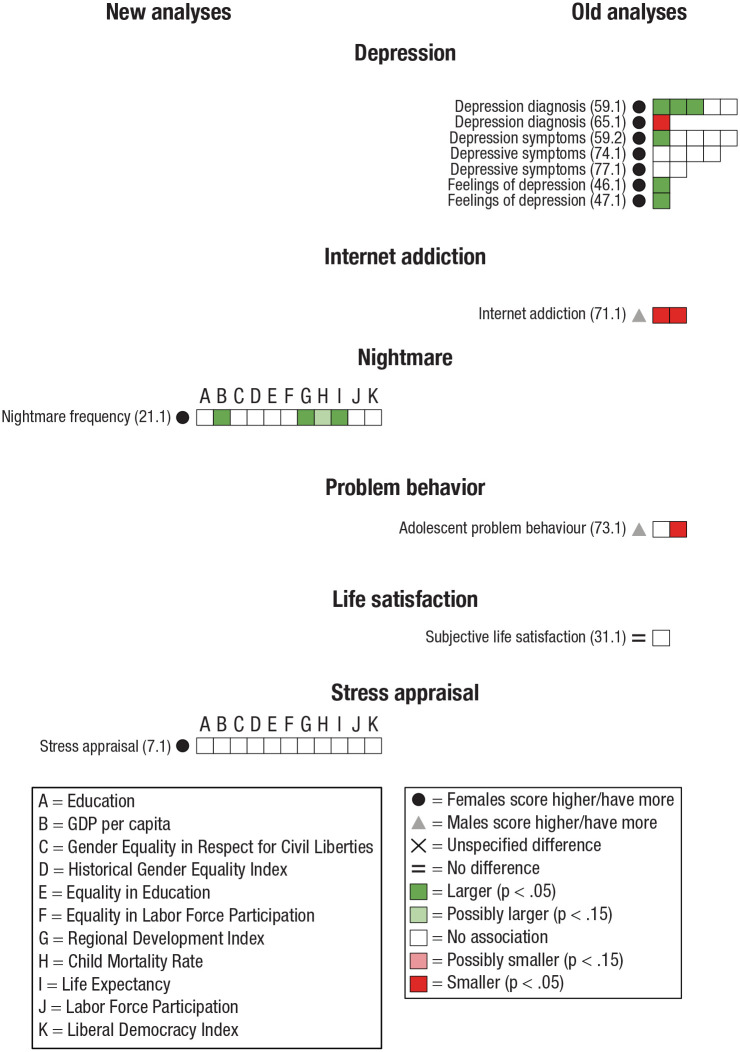
Results for each subcategory of Mental health. Significant overall sex differences are indicated with symbols (see right side of legend). Each square denotes a tested association between a dependent variable, listed to the left of the square, and an indicator of a living condition (see left side of legend for indicators used in new analyses). A dark green square indicates a significant (*p* < .05) association, in which higher living conditions are associated with larger sex differences. A light green square indicates a marginally significant (*p* < .15) association of the same sort (applicable only for new analyses). A dark red square indicates a significant (*p* < .05) association, in which higher living conditions are associated with smaller sex differences. A light red square indicates a marginally significant (*p* < .15) association of the same sort (applicable only for new analyses). A white square indicates a nonsignificant association between living conditions and sex differences (*p* > .05 for the old analyses; *p* > .15 for the new analyses). Numbers within parentheses (x.y) indicate study (x) and dependent variable (y) analyzed, with corresponding numbers given in the data tables (see the column Dependent Variable Number in Table S2 in the Supplemental Material). Studies: (7) Davis et al. (1999), (21) Schredl and Reinhard (2011), (31) Batz-Barbarich et al. (2018), (46) Hopcroft and Bradley (2007), (47) Hopcroft and McLaughlin (2012), (59) Salk et al. (2017), (65) Seedat et al. (2009), (71) Su et al. (2019), (73) Thijs et al. (2015), (74) Van de Velde et al. (2013), (77) Wang et al. (2016).

For an overview of the results for the category Mental health, see Figure 7.

#### Summary of mental health

Our results suggest that in countries with higher living conditions, sex differences are larger in outcomes in which females score higher than males, such as depression (feelings, symptoms, diagnosis) and nightmare frequency. In addition, sex differences tend to be smaller in countries with higher living conditions in outcomes showing higher levels in males than in females (e.g., internet addiction and problem behavior in adolescence). However, apart from the subcategory Depression, there is only one study per subcategory, indicating that more research is needed in this area.

### Academic self-concept

Academic self-concept is a construct relating to attitudes, self-efficacy, self-confidence, and motivation toward STEM-oriented school subjects or to academics in general. We subdivided the category Academics self-concept into Self-concept STEM (e.g., self-confidence in STEM) and Self-concept general academics (self-esteem toward academics in general).

#### Sex differences in academic self-concept

Considering both the old analyses and new analyses, we found that males show higher Self-concept STEM (SMDs = 0.10–0.33; Else-Quest et al., 2010; Goldman & Penner, 2016; Huang, 2013; Stoet & Geary, 2018). The one study in the new analyses that investigated Self-concept general academics (general academic self-efficacy) showed no sex difference (Huang, 2013). Taken together, there seems to be an advantage for males regarding interest, motivation, and self-efficacy directed toward STEM.

#### Living conditions and academic self-concept

Regarding analyses conducted with data from either PISA or TIMSS on Self-concept STEM, there are 19 analyses in the old analyses that showed associations suggesting that sex differences are larger in societies with higher living conditions (Else-Quest et al., 2010; Goldman & Penner, 2016; Stoet & Geary, 2018). By contrast, there are 13 analyses in the old analyses that indicated that these sex differences are smaller (Else-Quest et al., 2010), and 50 analyses indicated no associations between living conditions and the magnitude of sex differences in Self-concept STEM. Thus, according to data from PISA or TIMSS, it does not seem to be a general association between sex differences in Self-concept STEM and living conditions. However, the new analyses indicated that sex differences favoring men are smaller in countries with higher living conditions in Self-concept STEM, focusing specifically on math and computer self-efficacy (Huang, 2013).

There is only one study (Huang, 2013) in the new analyses in which the associations between sex differences and living conditions for Self-concept general academics could be analyzed. No association between sex differences and living conditions was found.

**Fig. 8.**
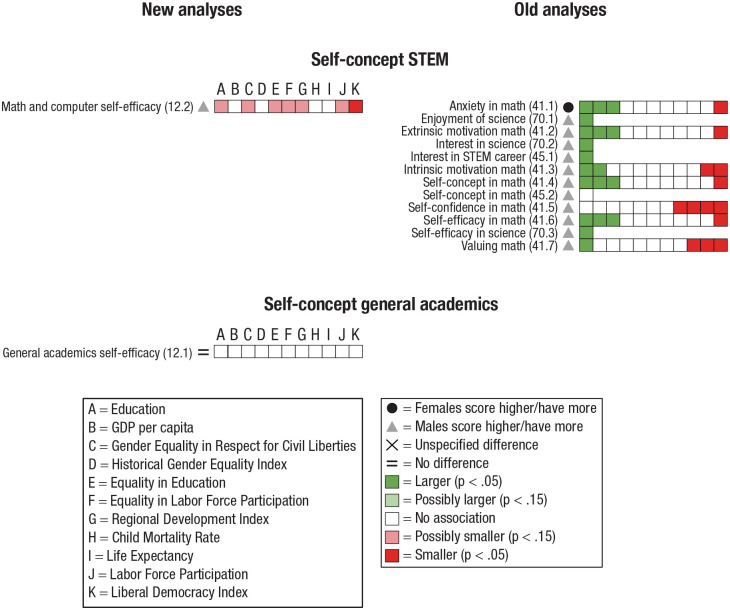
Results for each subcategory of Academic self-concept. Significant overall sex differences are indicated with symbols (see right side of legend). Each square denotes a tested association between a dependent variable, listed to the left of the square, and an indicator of a living condition (see left side of legend for indicators used in new analyses). A dark green square indicates a significant (*p* < .05) association, in which higher living conditions are associated with larger sex differences. A light green square indicates a marginally significant (*p* < .15) association of the same sort (applicable only for new analyses). A dark red square indicates a significant (*p* < .05) association, in which higher living conditions are associated with smaller sex differences. A light red square indicates a marginally significant (*p* < .15) association of the same sort (applicable only for new analyses). A white square indicates a nonsignificant association between living conditions and sex differences (*p* > .05 for the old analyses; *p* > .15 for the new analyses). Numbers within parentheses (x.y) indicate study (x) and dependent variable (y) analyzed, with corresponding numbers given in the data tables (see the column Dependent Variable Number in Table S2 in the Supplemental Material). Studies: (12) Huang (2013), (41) Else-Quest et al. (2010), (45) Goldman and Penner (2018), (70) Stoet and Geary (2018).

For an overview of the results for the category Academic self-concept, see Figure 8.

#### Summary of academic self-concept

The results for academic self-concept are inconclusive. We found that for Self-concept STEM, mainly from PISA and TIMSS data, the pattern is mixed such that most studies indicated both larger and smaller sex differences in countries with higher living conditions. Note, however, that sex differences in math and computer self-efficacy appear to be smaller in countries with higher living conditions.

### Morals and values

Morals can be defined as the prevailing standards within a group or what society sanctions as correct, whereas values typically relate to the judgment of what is important in life. We subdivided the category Morals and values into Morality (justice- and care-oriented morality) and Values (e.g., marriage defense, hedonism, tradition).

#### Sex differences in morals and values

A male advantage is seen for justice-oriented morality (SMD = 0.26; Jaffee & Hyde, 2000), whereas females have higher care-oriented morality (SMD = 0.34; Jaffee & Hyde, 2000). The two studies that examined Values showed that there are some measures in which females score higher (benevolence, tradition, universalism, marriage defense; Day et al., 2011; Schwartz & Rubel-Lifschitz, 2009) and some measures in which males score higher (power, stimulation, achievement, self-direction, hedonism (Schwartz & Rubel-Lifschitz, 2009).

#### Living conditions and morals and values

There are no systematic associations between living conditions and sex differences in Morality. For Values, one study, using two databases (Schwartz & Rubel-Lifschitz, 2009), indicated that sex differences in several aspects of values are larger in countries with higher living conditions. The other study (Day et al., 2011) indicated that females’ higher levels of marriage defense may be smaller in countries with higher living conditions.

**Fig. 9.**
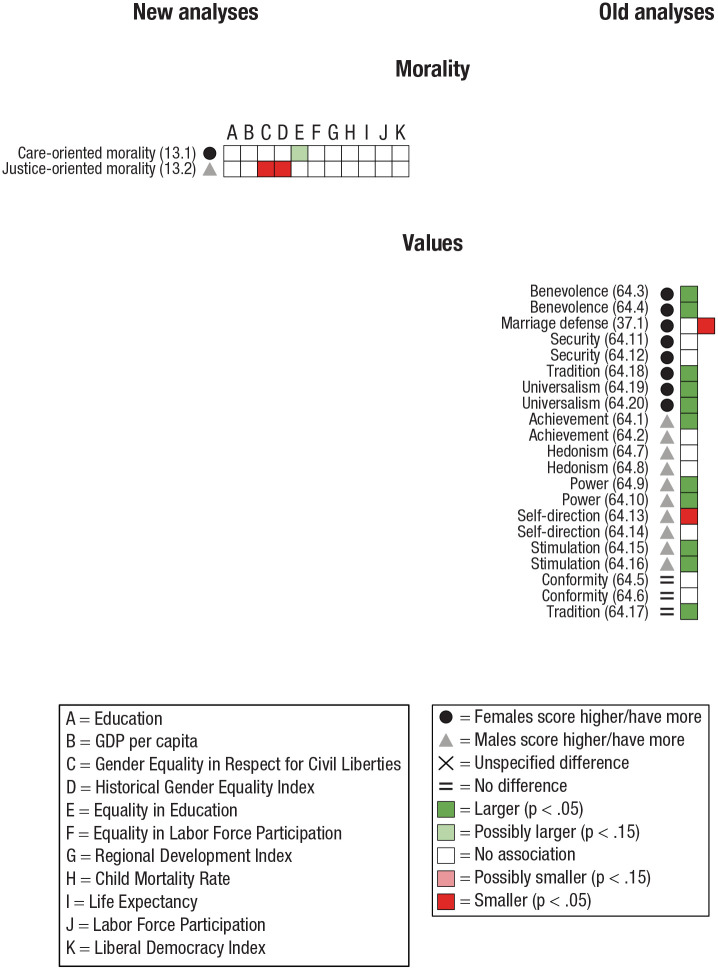
Results for each subcategory of Morals and values. Significant overall sex differences are indicated with symbols (see right side of legend). Each square denotes a tested association between a dependent variable, listed to the left of the square, and an indicator of a living condition (see left side of legend for indicators used in new analyses). A dark green square indicates a significant (*p* < .05) association, in which higher living conditions are associated with larger sex differences. A light green square indicates a marginally significant (*p* < .15) association of the same sort (applicable only for new analyses). A dark red square indicates a significant (*p* < .05) association, in which higher living conditions are associated with smaller sex differences. A light red square indicates a marginally significant (*p* < .15) association of the same sort (applicable only for new analyses). A white square indicates a nonsignificant association between living conditions and sex differences (*p* > .05 for the old analyses; *p* > .15 for the new analyses). Numbers within parentheses (x.y) indicate study (x) and dependent variable (y) analyzed, with corresponding numbers given in the data tables (see the column Dependent Variable Number in Table S2 in the Supplemental Material). Studies: (13) Jaffee and Hyde (2000), (37) Day et al. (2011), (64) Schwartz and Rubel-Lifschitz (2009).

For an overview of the results for the category Morals and values, see Figure 9.

#### Summary of morals and values

There is weak evidence to suggest that sex differences in many aspects of values are larger in countries with higher living conditions, although this pattern does not seem to apply to marriage defense. More research is warranted because these patterns are based on only two studies from three databases.

### Indicators of living conditions

We classified the more than 70 indicators of living conditions used in the old analyses (see Table S1 in the Supplemental Material) and new analyses into five categories: Economy, Education, Gender equality, Human development, and Other. As shown in Table 1, the indicator category most often reliably associated with sex differences in psychological capacities and behaviors, both in the old analyses and the new analyses, was Economy, closely followed by Human development and Other in the old analyses and by Other and Education in the new analyses. Indicators of Gender equality are among the least sensitive in predicting the extent of sex differences, both in the old analyses and new analyses. There was also a large difference in the percentage of significant results between the old analyses and the new analyses; the old analyses yielded approximately 48% significant associations, and the new analyses yielded only 15%. This difference was smaller but still substantial when instead considering associations with *p* values < .15 (see the column Significant associations, lenient in Tables 1 and 2) in the new analyses, raising the proportion of significant results to 30%. More associations indicated that there are larger sex differences in countries with higher living conditions both in the old analyses (30% vs. 18%) and the new analyses (21% vs. 10%, including *p* values <.15).

**Table 1.** Overview of Associations Between Indicators of Living Conditions and Sex Differences, Divided by Type of Analysis (New Analyses and Old Analyses) and Living-Conditions Category (Education, Economy, Gender Equality, Human Development, Other)

Analysis	Living-conditions category	Significant associations	Significant associations, lenient	Larger	Possibly larger	No association	Possibly smaller	Smaller
New analyses	Economy	9/45 (20%)	18/45 (40%)	7/45 (16%)	6/45 (13%)	27/45 (60%)	3/45 (7%)	2/45 (4%)
	Education	7/45 (16%)	15/45 (33%)	6/45 (13%)	3/45 (7%)	30/45 (67%)	5/45 (11%)	1/45 (2%)
	Gender equality	21/180 (12%)	49/180 (27%)	15/180 (8%)	17/180 (9%)	131/180 (73%)	11/180 (6%)	6/180 (3%)
	Human development	27/180 (15%)	56/180 (31%)	21/180 (12%)	19/180 (11%)	124/180 (69%)	10/180 (6%)	6/180 (3%)
	Other	8/45 (18%)	12/45 (27%)	5/45 (11%)	4/45 (9%)	33/45 (73%)	0/45 (0%)	3/45 (7%)
	All categories	72/495 (15%)	150/495 (30%)	54/495 (11%)	49/495 (10%)	345/495 (70%)	29/495 (6%)	18/495 (4%)
Old analyses	Economy	28/50 (56%)	—	23/50 (46%)	—	22/50 (44%)	—	5/50 (10%)
	Education	11/34 (32%)	—	6/34 (18%)	—	23/34 (68%)	—	5/34 (15%)
	Gender equality	135/282 (48%)	—	74/282 (26%)	—	147/282 (52%)	—	61/282 (22%)
	Human development	37/64 (58%)	—	25/64 (39%)	—	27/64 (42%)	—	12/64 (19%)
	Other	24/45 (53%)	—	19/45 (42%)	—	21/45 (47%)	—	5/45 (11%)
	All categories	235/495 (48%)	—	147/495 (30%)	—	240/495 (48%)	—	88/495 (18%)

Note: Ratios and percentages are given when sex differences are larger (*p* < .05), possibly larger (*p* < .15; applicable only for new analyses), smaller (*p* < .05), or possibly smaller (*p* < .15; applicable only for new analyses) or if associations cannot be detected. The combined ratios and percentages of the columns Larger and Smaller are given in Significant associations. The column Significant associations, lenient also includes the added values of Possibly larger and Possibly smaller.

**Table 2.** Overview of Associations Between Indicators of Living Conditions and Sex Differences, Divided by Type of Analysis (New Analyses and Old analyses), Living-Conditions Category (Education, Economy, Gender Equality, Human Development, Other), and Living-Conditions Subcategory

Analysis	Living-conditions category	Living-conditions subcategory	Significant associations	Significant associations, lenient	Larger	Possibly larger	No association	Possibly smaller	Smaller
New analyses	Economy	Logged GDP per capita	9/45 (20%)	18/45 (40%)	7/45 (16%)	6/45 (13%)	27/45 (60%)	3/45 (7%)	2/45 (4%)
	Education	Average years of schooling	7/45 (16%)	15/45 (33%)	6/45 (13%)	3/45 (7%)	30/45 (67%)	5/45 (11%)	1/45 (2%)
	Gender equality	GECL	5/45 (11%)	9/45 (20%)	2/45 (4%)	2/45 (4%)	36/45 (80%)	2/45 (4%)	3/45 (7%)
		HGEI	7/45 (16%)	13/45 (29%)	5/45 (11%)	4/45 (9%)	32/45 (71%)	2/45 (4%)	2/45 (4%)
		Equality in education	3/45 (7%)	12/45 (27%)	3/45 (7%)	6/45 (13%)	33/45 (73%)	3/45 (7%)	0/45 (0%)
		Equality in labor-force participation	6/45 (13%)	15/45 (33%)	5/45 (11%)	5/45 (11%)	30/45 (67%)	4/45 (9%)	1/45 (2%)
	Human development	RDI	7/45 (16%)	17/45 (38%)	6/45 (13%)	5/45 (11%)	28/45 (62%)	5/45 (11%)	1/45 (2%)
		Child-mortality rate	6/45 (13%)	12/45 (27%)	5/45 (11%)	5/45 (11%)	33/45 (73%)	1/45 (2%)	1/45 (2%)
		Life expectancy	9/45 (20%)	14/45 (31%)	7/45 (16%)	2/45 (4%)	31/45 (69%)	3/45 (8%)	2/45 (4%)
		Labor-force participation	5/45 (11%)	13/45 (29%)	3/45 (7%)	7/45 (16%)	32/45 (71%)	1/45 (2%)	2/45 (4%)
	Other	LDI	8/45 (18%)	12/45 (27%)	5/45 (11%)	4/45 (9%)	33/45 (73%)	0/45 (0%)	3/45 (7%)
	All categories	All subcategories	72/495 (15%)	150/495 (30%)	54/495 (11%)	49/495 (10%)	345/495 (70%)	29/495 (6%)	18/495 (4%)
Old analyses	Economy	Economy-GDP	19/29 (66%)	—	14/29 (48%)	—	10/29 (34%)	—	5/29 (17%)
		Economy-other	9/21 (43%)	—	9/21 (43%)	—	12/21 (57%)	—	0/21 (0%)
	Education	Education	11/34 (32%)	—	6/34 (18%)	—	23/34 (68%)	—	5/34 (15%)
	Gender equality	GE-composite indicators	91/194 (47%)	—	54/194 (28%)	—	103/194 (53%)	—	37/194 (19%)
		GE-representation	26/50 (52%)	—	10/50 (20%)	—	24/50 (48%)	—	16/50 (32%)
		GE-economy	4/21 (19%)	—	0/21 (0%)	—	17/21 (81%)	—	4/21 (19%)
		GE-education	7/7 (100%)	—	5/7 (71%)	—	0/7 (0%)	—	2/7 (29%)
		GE-culture	5/7 (71%)	—	3/7 (43%)	—	2/7 (29%)	—	2/7 (29%)
		GE-life expectancy	2/3 (67%)	—	2/3 (67%)	—	1/3 (33%)	—	0/3 (0%)
	Human development	HD-composite indicators	10/21 (48%)	—	7/21 (33%)	—	11/21 (52%)	—	3/21 (14%)
		HD-fertility	11/13 (85%)	—	4/13 (31%)	—	2/13 (15%)	—	7/13 (54%)
		HD-health	16/27 (59%)	—	14/27 (52%)	—	11/27 (41%)	—	2/27 (7%)
		HD-labor	0/3 (0%)	—	0/3 (0%)	—	3/3 (100%)	—	0/3 (0%)
	Other	Other-Hofstede	19/36 (53%)	—	15/36 (42%)	—	17/36 (47%)	—	4/36 (11%)
		Other-miscellaneous	5/9 (56%)	—	49 (44%)	—	4/9 (44%)	—	1/9 (11%)
	All categories	All subcategories	235/495 (47%)	—	147/495 (30%)	—	240/495 (48%)	—	88/495 (18%)

Note: Ratios and percentages are given when sex differences are larger (*p* < .05), possibly larger (*p* < .15; applicable only for new analyses), smaller (*p* < .05), or possibly smaller (*p* < .15; applicable only for new analyses) or if an association cannot be detected. The combined ratios and percentages of the columns Larger and Smaller are given in Significant associations. The column Significant associations, lenient also includes the added values of Possibly larger and Possibly smaller. GDP = gross domestic product; GECL = Gender Equality in Respect for Civil Liberties; HGEI = Historical Gender Equality Index; RDI = Regional Development Index; LDI = Liberal Democracy Index; GE = Gender equality; HD = Human development.

By summarizing the results into subcategories within each indicator category, the results are more detailed. However, they are based on fewer analyses and therefore less reliable. Table 2 shows again that the results indicate that the subcategory Economy-GDP is relatively effective in predicting the magnitude of sex differences for both the old analyses and new analyses, although HD-fertility in the old analyses is an even stronger predictor.

## General Discussion

Our primary aim in this study was to review and systematize previous literature that examined the association between living conditions and psychological sex differences (old analyses). We summarized 54 studies and 133 dependent variables in the old analyses. The secondary aim was to investigate associations between living conditions and sex differences by using data mainly from meta-analyses to broaden and replicate previously published findings and avoid publication bias (new analyses). We analyzed an additional 27 studies and 45 dependent variables in the new analyses. Taken together, more than 70 indicators of living conditions have been used (see Table S1 in the Supplemental Material). We draw three conclusions from our analysis. First, sex differences in personality, verbal episodic memory, verbal ability, aggressive behavior, female negative emotions, depression, and general self-esteem are larger in countries with higher living conditions. By contrast, sex differences in mathematics, semantic memory, sexual behavior, partner preferences, and aggression toward partners are smaller in countries with higher living conditions. No systematic associations were observed between sex differences in the remaining assessed psychological capacities and behaviors (e.g., Academic Self-Concept, Spatial skills) and living conditions. Second, although results indicate that most of the assessed indicators of living conditions are not associated with the magnitude of the sex differences, results from both the old analyses and new analyses demonstrate that more sex differences are larger, rather than smaller, in countries with higher living conditions. Moreover, third, it seems that economic indicators of living conditions (e.g., GDP) are the most sensitive indicators in detecting associations with the magnitude of sex differences and that indicators of gender equality are less sensitive. Below, we discuss these findings, their relevance, and some limitations of the study.

### Conclusion 1: sex differences in countries with higher living conditions

How should one understand the pattern showing that men and women tend to be more alike in some regards and more dissimilar in others in countries with higher living conditions? The explanations are probably not the same for all psychological dimensions.

#### Personal characteristics

The categories showing the most consistent and convincing results are those relating to personality, demonstrating that sex differences are larger in countries with higher living conditions. This effect, sometimes referred to as the “gender-equality paradox,” has been reported previously. It has been argued that these findings lend support to the resource hypothesis over the social-role hypothesis. The resource hypothesis rests on the assumption that free expression of preferences requires meeting basic material needs and that when basic needs are met, sex-specific goals can develop. Thus, countries with higher living conditions presumably would allow females and males to pursue the values they care about more freely (i.e., presumably not because of social-role expectations). A more free expression would, in turn, result in larger sex differences in countries with higher living conditions. This hypothesis is supported by results from the old analyses (Costa et al., 2001; Falk & Hermle, 2018; Giolla & Kajonius, 2019; Kaiser, 2019) and, more importantly, by results from the new analyses (Grijalva et al., 2015; Miller et al., 2008; Randler & Engelke, 2019; Yang & Girgus, 2019).

#### Cognition

For cognition, some sex differences show a female advantage, and others show a male advantage. Our results suggest that in those cognitive abilities in which females perform at a higher level than men (episodic memory, verbal abilities), sex differences are larger in countries with higher living conditions. By contrast, sex differences in those cognitive abilities in which males perform at a higher level (math, semantic memory), sex differences are smaller in countries with higher living conditions. These data suggest that more than men, females benefit cognitively from societal improvements, giving rise to larger sex differences in episodic memory and verbal abilities and smaller sex differences in math and semantic memory. This interpretation of the results is in line with other findings showing that increased exposure to cognitive stimulation, economic prosperity, health improvements, and changes in average family size is associated with increases in cognitive performance over time (i.e., the Flynn effect; Flynn, 1987; Pietschnig & Voracek, 2015). Our results are also consistent with those that showed that females have a larger Flynn effect than males (Weber et al., 2017). Although it is still an open question why women appear to be more positively affected by improved living conditions than men, it could be hypothesized that women benefit disproportionately from societal improvements because they may start from a more disadvantaged position. This assumption can be compared with a group with only 1 year of schooling that would experience a greater improvement from an extra year compared with a group that had several years of education. However, spatial skills do not seem to follow this pattern; one study indicated larger sex differences (favoring males) in countries with higher living conditions (Lippa et al., 2010), and other studies showed no association with living conditions. Thus, more research on spatial abilities is needed before concluding that higher living conditions are associated with smaller sex differences in all those cognitive abilities for which males have an advantage.

#### Interpersonal behavior

Another area in which the pattern of results is consistent and systematic is sexuality and partner preference, for which differences are smaller in countries with higher living conditions. Sex differences in sexual behavior have typically been explained from an evolutionary perspective through the parental-investment theory (Schmitt, 2005; Trivers, 1972). The theory underlines that males and females differ significantly in the time and energy devoted to the offspring, mostly because females carry the child for 9 months and because unrestricted sexual behavior can yield only one offspring at a time. By contrast, the number of offspring a male can produce during the same period is substantially higher. Because of this, unrestricted sexual behavior is a more viable evolutionary strategy for males than for females. However, this evolutionary explanation does not preclude that environmental factors can further restrict sexuality. Results that showed sex differences in sexual behavior are smaller in countries with higher living conditions imply that female sexuality is affected by living conditions (e.g., availability of contraceptives or cultural restrictions on freely pursuing sexual desires). Sex differences in partner preference have often been explained from an evolutionary perspective as well (Buss & Schmitt, 2019). According to this perspective, males prefer features that signal youth and fertility, whereas females focus more on status and resource acquisition. Our results show that sex differences in partner preference are smaller in countries with higher living conditions, suggesting that these preferences to some degree depend on living conditions such as women’s earning capacity and cultural norms.

#### Emotions and mental health

Results for sex differences in emotions and mental health show some consistency. It seems that sex differences in those negative emotions that are more prevalent/stronger in females are more prominent in countries with higher living conditions. By contrast, the female advantage in feelings of happiness is smaller in those countries. Consistent with this pattern, there is weak evidence suggesting that sex differences in negative emotions that are more prevalent/stronger in males are smaller in countries with higher living conditions. Also in accordance with this pattern are findings in mental health showing that females’ higher levels of symptoms and diagnosed depression are greater in countries with higher living conditions. Overall, these results suggest that higher living conditions are associated with higher adverse effects on emotions and mental health in females than in males.

### Conclusion 2: implications of robust sex differences

As illustrated by the large proportion of sex differences that remain unaffected by living conditions, the robustness of the sex differences across countries is striking. Although many of the sex differences reported in the 54 studies reviewed in the old analyses and the 27 meta-analyses analyzed in the new analyses are small, they are systematic and reliable across countries. Moreover, the old analyses and the new analyses results demonstrate that more sex differences are larger, rather than smaller, in countries with higher living conditions. What are the implications of these findings? If there is a causal relationship between living conditions and psychological sex differences, and not just a correlation, one important implication is that there is little to indicate that psychological sex differences will disappear in the future even if living conditions, including gender equality, continue to increase.

Although many of the psychological sex differences are minor, several small sex differences in fundamental areas of psychological functioning may generate larger differences in everyday composite behaviors (Eagly & Revelle, 2022). For instance, considering personal characteristics, including personality and traits such as self-esteem and altruism, we find that they affect people’s behaviors and choices in many situations. Among other things, Big Five traits predict political preferences. To illustrate, people who score high in openness to experience and low in conscientiousness tend to be more left-leaning (Mondak & Halperin, 2008). By extension, larger sex differences in these traits might reinforce political divisions along gender lines. Arguably, then, even a “small” difference could have considerable effects.

An area that has received plenty of attention from policymakers and others is sex differences in educational and occupational choices. Attempts have been made in countries with relatively high levels of gender equality to reduce these differences. Research shows that cognitive strengths are related to academic strengths and that individuals adjust their educational and occupational choices with their academic strengths (Dekhtyar et al., 2018). Because more males exhibit technical/numerical skills and more females exhibit verbal/language skills, these findings imply that sex differences in academic skills likely contribute to the horizontal gender segregation in education and occupation (Dekhtyar et al., 2018). A further implication is that gender segregation in education and occupation will be difficult to avoid altogether, at least if individuals can freely pursue educational and occupational career paths and base these choices on academic strengths.

### Conclusion 3: important indicators

We found that indicators of economy (mainly GDP) were more sensitive in detecting associations with sex differences than indicators of gender equality in both the old analyses and new analyses. Indicators of gender equality displayed a relatively small proportion of significant results (12% in the new analyses and 48% in the old analyses). By contrast, indicators of economic development displayed a higher proportion of significant results (20% in the new analyses and 56% in the old analyses). A priori, it might seem that indicators taking dimensions of gender equality into account should be the most predictive of psychological sex differences. The fact that this was not the case might be connected to differences in the historical and cross-national availability of the indicators. Economic indicators, such as GDP, are available for most countries as far back as 1960. By contrast, most indicators of gender equality are either not time-specific or start only in the mid-1990s. This relative lack of variance in the gender-equality data may make effects less apparent.

Another possible reason as to why economic indicators are particularly sensitive in detecting associations with psychological sex differences concerns the way greater economic prosperity alters educational and occupational structures (e.g., K. Block et al., 2022; Charles & Bradley, 2009; Wong & Charles, 2020). In most countries, female labor-force participation has increased substantially in the last century (Ortiz-Ospina & Tzvetkova, 2017). Nonetheless, occupational gender segregation is notable. For instance, globally, a large part of people working in health care, education, and social services are female, whereas a greater share of males is found in areas requiring physical strength and technical skills (e.g., Statistics Sweden, 2020; Wong & Charles, 2020). Occupational gender segregation also manifests as a greater share of females than males working part-time, a form of employment that is more prevalent in countries with higher living conditions (e.g., Ortiz-Ospina & Tzvetkova, 2017). Plausibly, the economically driven growth of these female-dominated sectors and forms of employment would strengthen and reinforce psychological sex differences. As more females enter the labor market in “female” professional roles, they might internalize the “female” values of these roles, such as the care-oriented values of some health-care professions.

This point is central to the social-role theory proposed by Eagly and Wood (1999, 2012). They argued that the gendered division of labor is originally rooted in sex differences in physical attributes. These differences led women and men to take on different social roles that, in turn, led to shared beliefs, or stereotypes, regarding males’ and females’ social roles. With increased economic prosperity and the appearance of new jobs viewed as suitable to the female social role, more women become socialized to the occupations they pursue. We would thus expect to find larger psychological sex differences in countries with higher living conditions. This offers an explanation of the gender-equality paradox that is distinct from those that rely on innate gender-specific preferences. To further investigate the viability of the respective explanations, comprehensive year- and country-specific indicators of occupational gender segregation would be needed. Analyses of these data could help to determine whether occupational gender segregation is the reason why economic indicators were most sensitive in predicting psychological sex differences.

### Advantages and limitations

Our systematic review has several advantages. First, the search for published articles was done systematically and objectively, thereby ensuring that we identified the most relevant articles. Second, we could broaden and replicate previously published findings and avoid publication bias by empirically investigating associations between living conditions and sex differences because the results in the new analyses have never been analyzed, presented, or published previously.

Although our review of previous results and new analyses have contributed to a better understanding of the associations between indicators of living conditions and psychological sex differences, it also has some limitations. First, relatively few studies on psychological dimensions have collected data from enough countries to warrant inclusion in the present study. Furthermore, the difficulties in collecting reliable data from many countries have sometimes forced researchers to rely on the same data sets, leading to similar research questions and analyses. As a result, we have some duplicate findings in the old analyses, often regarding the PISA data set. Taken together, we present a limited and nonexhaustive depiction of psychological sex differences and the association between these sex differences and living conditions.

The old analyses, comprising published analyses, often provide a more coherent pattern of findings than the new analyses, which present previously unpublished results. This fact might indicate publication bias, that is, a state of affairs in which the publication of results depends on the significance and direction of effects detected and not solely on research quality. However, other factors may contribute to this pattern as well. The old analyses included a greater proportion of data for which tests and tasks were the same in all assessed countries and study sites, making data less susceptible to noise, thereby making effects more easily detectable. By contrast, in the new analyses, we always used data collected for meta-analyses, which typically consist of data from many studies, often using different tests and instruments to assess sex differences in the underlying psychological dimensions. Thus, these studies inherently have more noise in the data. The new analyses may therefore provide a more conservative test of the hypotheses but also possibly hide true effects. To detect more nuance and, hopefully, identify systematic but weak patterns of results, we chose to also report marginally significant associations.

Moreover, for many studies in the old analyses, there may have been prior theoretical grounds or findings underlying the decision to investigate associations between living conditions and certain sex differences. A collection of studies taking this approach is likely to find a greater proportion of significant effects than a less selective investigation of many areas. Indeed, the psychological dimensions investigated in the old analyses are rarely the same as those in the new analyses.

Limitations can occur when investigating potential associations between behavioral measures and living conditions. Researchers’ analyses are generally confined by the limited number of publicly available indicators, often provided by the UN, World Bank, and OECD. These indicators typically offer crude summary measures of the underlying construct of interest. Furthermore, the indicators may not be available for all years/countries of interest, and this absence became more of a problem further back in time. The limited availability of early data is important because it may lead to an underestimation of the actual associations between living conditions and psychological sex differences. For instance, for any given age cohort, the living conditions of greatest interest are likely those of the participants’ formative years, not at the age when being assessed. Having no access to the living conditions from the formative years, which may be the case with older participants, is a troublesome constraint. Taken together, indicators of living conditions can be unspecific measures of the actual living conditions and may therefore provide an underestimation of their real effect.

**Fig. 10.**
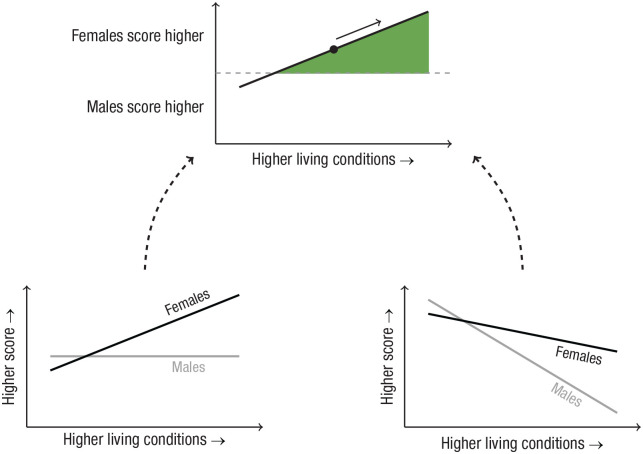
Illustration of how two distinct patterns of sex differences can produce the same observed outcome. Given that the type of data that we have extracted consists of contrasts between males and females (i.e., effect sizes) rather than absolute values that are comparable between studies, we cannot deduce the underlying pattern making up these contrasts. For example, the relationship between living conditions and sex differences depicted in the top part of the figure, showing that sex differences favoring females are more extensive in countries with higher living conditions, could be a result of either one of the data patterns below. In the left figure, females score higher in countries with higher living conditions, and in the right figure, both males and females score lower/have less in countries with higher living conditions.

Another limitation is that when we state that there are larger or smaller sex differences in countries with higher living conditions, the underlying pattern of male and female scores cannot be determined (for an illustration and further explanation, see Fig. 10). This problem arises because data from meta-analyses comprise contrasts between males and females (i.e., effect sizes) rather than absolute values that are comparable between studies.^5^ Future research using digital data collections of the same measures in wider geographic settings will allow researchers to address such issues.

Furthermore, we have investigated only linear relationships between living conditions and sex differences in the new analyses despite the possibility of nonlinear associations. The main reason is that it enabled us to compare our results with those in the old analyses more easily. Future large-scale studies should investigate such potential nonlinear associations between living conditions and sex differences.

### Summary

We found that sex differences in some cognitive functions, personality, and negative emotions are larger in countries with higher living conditions. We also found that some sex differences, notably in mathematics and sexual behavior, are smaller in countries with higher living conditions. Moreover, our study showed that economic indicators of living conditions, such as GDP, are the most sensitive indicators in predicting the magnitude of sex differences. The results of this systematic review and new analyses highlight various psychological sex differences and that the pattern of female and male strengths and weaknesses is the same in most countries examined. Our results also show that there are little data to suggest that men and women will become more alike as the world develops. Instead, policymakers should expect sex differences to mostly remain unchanged or become larger as the world develops, with important exceptions in sexual behavior and partner preferences.

Does this mean that there is a “gender-equality paradox”? Yes, a gender-equality paradox was indeed observed for some psychological dimensions. This refines the understanding of when and where gender-equality policies are associated with the expected changes in outcomes. However, we note that indicators of economic development, rather than gender equality, were most reliably associated with larger psychological sex differences. Often, several indicators (e.g., GDP and Gender equality) were associated with larger psychological sex differences, stressing that further research is warranted to better understand the causes that underlie these associations.

## Supplemental Material

sj-docx-1-pps-10.1177_17456916231202685 – Supplemental material for A Systematic Review and New Analyses of the Gender-Equality Paradox Supplemental material, sj-docx-1-pps-10.1177_17456916231202685 for A Systematic Review and New Analyses of the Gender-Equality Paradox by Agneta Herlitz, Ida Hönig, Kåre Hedebrant and Martin Asperholm in Perspectives on Psychological Science

sj-docx-2-pps-10.1177_17456916231202685 – Supplemental material for A Systematic Review and New Analyses of the Gender-Equality Paradox Supplemental material, sj-docx-2-pps-10.1177_17456916231202685 for A Systematic Review and New Analyses of the Gender-Equality Paradox by Agneta Herlitz, Ida Hönig, Kåre Hedebrant and Martin Asperholm in Perspectives on Psychological Science

## Transparency

*Action Editor:* Leonel Garcia-Marques

*Editor:* Interim Editorial Panel

## Notes

1. We use the term “sex” rather than “gender” here because it relates specifically to the construct (legal sex) underlying the dichotomy (male/female) used here and in the articles we examine, whereas “gender” relates to a social construct that is not exclusive to women and men. We use the term “gender” for roles and internal feelings and in association with equality and other aspects of living conditions in which “gender,” rather than “sex,” is typically used.

2. We use the terms “higher” and “lower” living conditions to illustrate, for instance, more years of education, higher GDP, or lower levels of infant mortality but also higher levels of individualism, the latter being based on a subjective judgment using Sweden (our home country) as a reference when deciding whether an endpoint of an ambiguous indicator is an example of higher or lower living conditions.

3. Throughout this article, we use the phrase “countries with higher/lower living conditions,” which in many cases is a simplification given that the indicators of living conditions may be both country- and time-specific.

4. More specifically, the Web of Science categories selected were: anthropology; behavioral sciences; biology; clinical neurology; criminology and penology; economics; education and educational research; environmental studies; evolutionary biology; family studies; language and linguistics; linguistics; neurosciences; psychiatry; psychology; psychology, applied; psychology, biological; psychology, clinical; psychology, developmental; psychology, educational; psychology, experimental; psychology, mathematical; psychology, multidisciplinary; psychology, psychoanalysis; psychology, social; public, environmental, and occupational health; rehabilitation; social issues; social sciences, interdisciplinary; sociology; substance abuse; women’s studies.

5. It would be possible to discern the underlying pattern in a few, specific cases for which all the data come from the same type of test/measurement if data were available for males and females separately. However, such circumstances are rare.
