# Supplementary material for: A Systematic Review and New Analyses of the Gender-Equality Paradox
Source: Perspect Psychol Sci. 2024 Jan 3;20(3):503–39. doi: 10.1177/17456916231202685 (PMC12065958; doi:10.1177/17456916231202685)
Supplement: sj-docx-1-pps-10.1177_17456916231202685 – Supplemental material for A Systematic Review and New Analyses of the Gender-Equality Paradox [file sj-docx-1-pps-10.1177_17456916231202685.docx]

**Table S1**

*Indicators Used in the Old Analyses, Divided Into Indicator Category and Subcategory, With Reference and Starting Year*

| **Living conditions category** | **Living conditions subcategory** | **Name** | **Main reference** | **Starting year** |
| --- | --- | --- | --- | --- |
| Economy | Economy-Other | GINI | OECD | 2004 |
|  |  | Public spending/GDP | OECD | 1980 |
|  |  | Government services | The World Fact Book | - |
|  | Economy-GDP | GDP per capita | World Bank | 1960 |
|  |  | GNI | World Bank | 1962 |
| Education | Education-Education | Primary enrollment ratio | World Bank | 1970 |
|  |  | Secondary enrollment ratio | World Bank | 1970 |
|  |  | Tertiary enrollment ratio | World Bank | 1970 |
|  |  | Literacy ratio | World Bank | 1975 |
|  |  | Percentage with university degree | World Bank | 1970 |
|  |  | Education index (EI) | UNDP | 1990 |
|  |  | Population edu + employment | UNDP/World Bank | 1990 |
| Gender Equality | GE-Composite indicators | Gender equality | Schwartz et al., 2009 | * |
|  |  | GGI | World Economic Forum | 2006 |
|  |  | SIGE | Dijkstra, 2002 | * |
|  |  | GEM | UNDP | 1995 |
|  |  | Gender Equality Index | Falk & Hermle, 2018 | - |
|  |  | Gender Development Index | UNDP | 1995 |
|  |  | GII | UNDP | 1995 |
|  |  | GEQ | White, 1997 | 1995 |
|  |  | Emancipation | Ebbeler et al., 2017 | - |
|  | GE-Culture | Gender role traditionality | WHO Mental Health Surveys | - |
|  |  | Gender stereotype differentiation | Williams & Best, 1990 | * |
|  |  | Traditional gender role attitudes | World Values Surveys | 1981 |
|  | GE-Economy | Economic activity rate female/male ratio | UNDP | 1995 |
|  |  | Womens/mens mean hourly earnings | OECD | 1977 |
|  |  | Women's wage equality | UNDP | 2001 |
|  |  | Gender gap in economy | World Economic Forum | 2006 |
|  | GE-Education | Female/male ratio in edu + labor force particip. | UNDP/World Bank | 1990 |
|  |  | Gender differences in education | UNDP/World Bank | 1980 |
|  |  | Women's illiteracy rate | World Bank | 1975 |
|  | GE-Life expectancy | Maternal mortality | The World Factbook | - |
|  |  | Women's life expectancy | UNSD | 1950 |
|  | GE-Representation | Women's suffrage (years) | World Economic Forum | * |
|  |  | Women' share of parliamentary seats | World Bank | 1960 |
|  |  | Women's share of research positions | OECD | 1989 |
|  |  | Executive positions | UNDP | 1995 |
|  |  | Labor participation rate (females) | UNDP | 1995 |
|  |  | Women's share of employment | OECD | 2000 |
|  |  | Women's share of higher labor market positions | UNDP | 1995 |
|  |  | % Women-headed households | UNSD | - |
| Human development | HD-Composite | Human Development Index | UNDP | 1990 |
|  |  | Regional Development Index | Weber et al., 2014 | 1950 |
|  | HD-Fertility | Mean female age marriage | UNSD | - |
|  |  | Contraceptive prevalence | World Bank | 1969 |
|  |  | Fertility | World Bank | 1960 |
|  |  | Teen birth rate | World Bank | 1960 |
|  |  | Contraception use | World Bank | 1969 |
|  | HD-Health | Life expectancy | Gapminder | 1800 |
|  |  | Historic pathogen prevalence | Fincher et al., 2008 | * |
|  |  | Pathogen stress | Gangestad & Buss, 1993; Low, 1990 | * |
|  |  | HIV/AIDS rate | World Bank | 1990 |
|  |  | Food consumption | Food and Agriculture Organization of the United Nations | - |
|  |  | Child malnutrition | World Bank | 1986 |
|  |  | Infant mortality | Gapminder | 1800 |
|  |  | Low birthweight | World Bank | 1969 |
|  | HD-Labor | Labor participation rate (males) | World Bank | 1990 |
| Other | Other-Hofstede | Cultural masculinity | Hofstede | * |
|  |  | Femininity | Hofstede | * |
|  |  | Femininity/masculinity | Hofstede | * |
|  |  | Individualism | Hofstede | * |
|  |  | Individualism-collectivism | Hofstede | * |
|  |  | Indulgence | Hofstede | * |
|  |  | Power Distance | Hofstede | * |
|  |  | Pragmatism | Hofstede | * |
|  |  | Uncertainty avoidance | Hofstede | * |
|  | Other-Miscellaneous | Level of democracy | van de Vliert et al. (1999) | - |
|  |  | Traditionalism | European Social Survey | 2002 |
|  |  | Rule of Law Index | The World Bank | 1996 |
|  |  | Quality of life | The World Fact Book | - |

*Note.* Information presented in this table is imprecise in that the indicators used in the 54 reviewed articles have frequently been named differently, used various references, and often been modified or constructed. Moreover, to summarize the data, we have attempted to categorize several indicators under the same *Name*. Thus, the table presents an underestimation of the number of indicators used. GE = Gender Equality. HD = Human Development. For *Starting year*, indicators that are not time-specific are marked with *. Indicators where *Starting year* cannot be resolved are marked with -.
