# Supplementary material for: A Systematic Review and New Analyses of the Gender-Equality Paradox
Source: Perspect Psychol Sci. 2024 Jan 3;20(3):503–39. doi: 10.1177/17456916231202685 (PMC12065958; doi:10.1177/17456916231202685)
Supplement: sj-docx-2-pps-10.1177_17456916231202685 – Supplemental material for A Systematic Review and New Analyses of the Gender-Equality Paradox [file sj-docx-2-pps-10.1177_17456916231202685.docx]

**Table S2***Simplified List of Data Used in the Old and New Analyses*

| **Study** | **Dependent variable number** | **Countries** | **Studies** | **Subjects** | **Living conditions subcategory** | **Association** |
| --- | --- | --- | --- | --- | --- | --- |
| Archer (2004) | 1.1 | 11 | 35 | 19986 | Child Mortality Rate | None |
| Archer (2004) | 1.1 | 11 | 34 | 19563 | Education | None |
| Archer (2004) | 1.1 | 11 | 34 | 19563 | Equality in Education | None |
| Archer (2004) | 1.1 | 11 | 34 | 19563 | GDP per capita | None |
| Archer (2004) | 1.1 | 11 | 35 | 19986 | GECL | None |
| Archer (2004) | 1.1 | 11 | 34 | 19563 | HGEI | None |
| Archer (2004) | 1.1 | 11 | 32 | 19380 | Labor Force Participation | None |
| Archer (2004) | 1.1 | 11 | 32 | 19380 | Equality in Labor Force Participation | None |
| Archer (2004) | 1.1 | 11 | 35 | 19986 | LDI | None |
| Archer (2004) | 1.1 | 11 | 35 | 19986 | Life Expectancy | Possibly larger |
| Archer (2004) | 1.1 | 11 | 34 | 19563 | RDI | None |
| Archer (2004) | 1.2 | 14 | 53 | 15901 | Child Mortality Rate | None |
| Archer (2004) | 1.2 | 14 | 53 | 15901 | Education | None |
| Archer (2004) | 1.2 | 14 | 53 | 15901 | Equality in Education | None |
| Archer (2004) | 1.2 | 14 | 53 | 15901 | GDP per capita | Possibly larger |
| Archer (2004) | 1.2 | 14 | 53 | 15901 | GECL | None |
| Archer (2004) | 1.2 | 14 | 53 | 15901 | HGEI | Possibly larger |
| Archer (2004) | 1.2 | 14 | 45 | 14173 | Labor Force Participation | Possibly larger |
| Archer (2004) | 1.2 | 14 | 45 | 14173 | Equality in Labor Force Participation | None |
| Archer (2004) | 1.2 | 14 | 53 | 15901 | LDI | None |
| Archer (2004) | 1.2 | 14 | 53 | 15901 | Life Expectancy | None |
| Archer (2004) | 1.2 | 14 | 53 | 15901 | RDI | None |
| Archer (2004) | 1.3 | 16 | 122 | 43781 | Child Mortality Rate | Larger |
| Archer (2004) | 1.3 | 16 | 122 | 43781 | Education | None |
| Archer (2004) | 1.3 | 16 | 122 | 43781 | Equality in Education | Possibly larger |
| Archer (2004) | 1.3 | 16 | 122 | 43781 | GDP per capita | Possibly larger |
| Archer (2004) | 1.3 | 16 | 122 | 43781 | GECL | Possibly larger |
| Archer (2004) | 1.3 | 16 | 122 | 43781 | HGEI | Larger |
| Archer (2004) | 1.3 | 16 | 89 | 35389 | Labor Force Participation | Larger |
| Archer (2004) | 1.3 | 16 | 89 | 35389 | Equality in Labor Force Participation | Larger |
| Archer (2004) | 1.3 | 16 | 122 | 43781 | LDI | Possibly larger |
| Archer (2004) | 1.3 | 16 | 122 | 43781 | Life Expectancy | Larger |
| Archer (2004) | 1.3 | 16 | 122 | 43781 | RDI | Possibly larger |
| Archer (2004) | 1.4 | 20 | 124 | 122947 | Child Mortality Rate | None |
| Archer (2004) | 1.4 | 19 | 122 | 121897 | Education | Possibly smaller |
| Archer (2004) | 1.4 | 19 | 122 | 121897 | Equality in Education | None |
| Archer (2004) | 1.4 | 18 | 122 | 121849 | GDP per capita | None |
| Archer (2004) | 1.4 | 18 | 124 | 122851 | GECL | None |
| Archer (2004) | 1.4 | 18 | 122 | 121849 | HGEI | None |
| Archer (2004) | 1.4 | 20 | 106 | 119875 | Labor Force Participation | None |
| Archer (2004) | 1.4 | 20 | 106 | 119875 | Equality in Labor Force Participation | Possibly larger |
| Archer (2004) | 1.4 | 18 | 124 | 122851 | LDI | None |
| Archer (2004) | 1.4 | 20 | 124 | 122947 | Life Expectancy | None |
| Archer (2004) | 1.4 | 18 | 122 | 121849 | RDI | None |
| Archer (2004) | 1.5 | 14 | 80 | 24728 | Child Mortality Rate | None |
| Archer (2004) | 1.5 | 14 | 80 | 24728 | Education | None |
| Archer (2004) | 1.5 | 14 | 80 | 24728 | Equality in Education | None |
| Archer (2004) | 1.5 | 14 | 80 | 24728 | GDP per capita | None |
| Archer (2004) | 1.5 | 14 | 80 | 24728 | GECL | None |
| Archer (2004) | 1.5 | 14 | 80 | 24728 | HGEI | None |
| Archer (2004) | 1.5 | 14 | 68 | 22839 | Labor Force Participation | None |
| Archer (2004) | 1.5 | 14 | 68 | 22839 | Equality in Labor Force Participation | None |
| Archer (2004) | 1.5 | 14 | 80 | 24728 | LDI | None |
| Archer (2004) | 1.5 | 14 | 80 | 24728 | Life Expectancy | None |
| Archer (2004) | 1.5 | 14 | 80 | 24728 | RDI | None |
| Else-Quest et al. (2012) | 10.1 | 23 | 180 | 54228 | Child Mortality Rate | None |
| Else-Quest et al. (2012) | 10.1 | 24 | 181 | 54619 | Education | Possibly larger |
| Else-Quest et al. (2012) | 10.1 | 24 | 181 | 54619 | Equality in Education | Possibly larger |
| Else-Quest et al. (2012) | 10.1 | 24 | 181 | 54619 | GDP per capita | Possibly larger |
| Else-Quest et al. (2012) | 10.1 | 24 | 181 | 54619 | GECL | None |
| Else-Quest et al. (2012) | 10.1 | 18 | 122 | 37403 | HGEI | None |
| Else-Quest et al. (2012) | 10.1 | 23 | 176 | 53504 | Labor Force Participation | None |
| Else-Quest et al. (2012) | 10.1 | 23 | 176 | 53504 | Equality in Labor Force Participation | None |
| Else-Quest et al. (2012) | 10.1 | 24 | 181 | 54619 | LDI | Possibly larger |
| Else-Quest et al. (2012) | 10.1 | 23 | 180 | 54228 | Life Expectancy | None |
| Else-Quest et al. (2012) | 10.1 | 24 | 181 | 54619 | RDI | None |
| Else-Quest et al. (2012) | 10.2 | 37 | 125 | 45783 | Child Mortality Rate | Possibly larger |
| Else-Quest et al. (2012) | 10.2 | 37 | 125 | 45983 | Education | Larger |
| Else-Quest et al. (2012) | 10.2 | 37 | 125 | 45983 | Equality in Education | None |
| Else-Quest et al. (2012) | 10.2 | 38 | 125 | 46070 | GDP per capita | Larger |
| Else-Quest et al. (2012) | 10.2 | 38 | 125 | 46070 | GECL | None |
| Else-Quest et al. (2012) | 10.2 | 33 | 71 | 25752 | HGEI | Possibly larger |
| Else-Quest et al. (2012) | 10.2 | 37 | 124 | 45581 | Labor Force Participation | Possibly larger |
| Else-Quest et al. (2012) | 10.2 | 37 | 124 | 45581 | Equality in Labor Force Participation | None |
| Else-Quest et al. (2012) | 10.2 | 38 | 125 | 46070 | LDI | Larger |
| Else-Quest et al. (2012) | 10.2 | 37 | 125 | 45783 | Life Expectancy | Larger |
| Else-Quest et al. (2012) | 10.2 | 38 | 125 | 46070 | RDI | Larger |
| Grijalva et al. (2015) | 11.1 | 18 | 221 | 297769 | Child Mortality Rate | None |
| Grijalva et al. (2015) | 11.1 | 19 | 215 | 296760 | Education | None |
| Grijalva et al. (2015) | 11.1 | 19 | 215 | 296760 | Equality in Education | None |
| Grijalva et al. (2015) | 11.1 | 19 | 222 | 301444 | GDP per capita | None |
| Grijalva et al. (2015) | 11.1 | 19 | 222 | 301444 | GECL | None |
| Grijalva et al. (2015) | 11.1 | 5 | 65 | 29804 | HGEI | Possibly larger |
| Grijalva et al. (2015) | 11.1 | 19 | 222 | 301444 | Labor Force Participation | Larger |
| Grijalva et al. (2015) | 11.1 | 19 | 222 | 301444 | Equality in Labor Force Participation | None |
| Grijalva et al. (2015) | 11.1 | 19 | 222 | 301444 | LDI | None |
| Grijalva et al. (2015) | 11.1 | 18 | 221 | 297769 | Life Expectancy | None |
| Grijalva et al. (2015) | 11.1 | 19 | 222 | 301444 | RDI | Possibly larger |
| Huang (2013) | 12.1 | 4 | 53 | 15553 | Child Mortality Rate | None |
| Huang (2013) | 12.1 | 5 | 55 | 16847 | Education | None |
| Huang (2013) | 12.1 | 5 | 55 | 16847 | Equality in Education | None |
| Huang (2013) | 12.1 | 5 | 55 | 16847 | GDP per capita | None |
| Huang (2013) | 12.1 | 5 | 55 | 16847 | GECL | None |
| Huang (2013) | 12.1 | 4 | 49 | 14178 | HGEI | None |
| Huang (2013) | 12.1 | 4 | 53 | 15553 | Labor Force Participation | None |
| Huang (2013) | 12.1 | 4 | 53 | 15553 | Equality in Labor Force Participation | None |
| Huang (2013) | 12.1 | 5 | 55 | 16847 | LDI | None |
| Huang (2013) | 12.1 | 4 | 53 | 15553 | Life Expectancy | None |
| Huang (2013) | 12.1 | 5 | 55 | 16847 | RDI | None |
| Huang (2013) | 12.2 | 9 | 121 | 26534 | Child Mortality Rate | None |
| Huang (2013) | 12.2 | 9 | 128 | 29851 | Education | Possibly smaller |
| Huang (2013) | 12.2 | 9 | 128 | 29851 | Equality in Education | Possibly smaller |
| Huang (2013) | 12.2 | 10 | 129 | 30016 | GDP per capita | None |
| Huang (2013) | 12.2 | 10 | 129 | 30016 | GECL | Possibly smaller |
| Huang (2013) | 12.2 | 8 | 117 | 25216 | HGEI | None |
| Huang (2013) | 12.2 | 9 | 121 | 26534 | Labor Force Participation | Possibly smaller |
| Huang (2013) | 12.2 | 9 | 121 | 26534 | Equality in Labor Force Participation | Possibly smaller |
| Huang (2013) | 12.2 | 10 | 129 | 30016 | LDI | Smaller |
| Huang (2013) | 12.2 | 9 | 121 | 26534 | Life Expectancy | None |
| Huang (2013) | 12.2 | 10 | 129 | 30016 | RDI | Possibly smaller |
| Jaffee & Hyde (2000) | 13.1 | 7 | 91 | 9697 | Child Mortality Rate | None |
| Jaffee & Hyde (2000) | 13.1 | 7 | 91 | 9697 | Education | None |
| Jaffee & Hyde (2000) | 13.1 | 7 | 91 | 9697 | Equality in Education | Possibly larger |
| Jaffee & Hyde (2000) | 13.1 | 7 | 91 | 9697 | GDP per capita | None |
| Jaffee & Hyde (2000) | 13.1 | 7 | 91 | 9697 | GECL | None |
| Jaffee & Hyde (2000) | 13.1 | 7 | 91 | 9697 | HGEI | None |
| Jaffee & Hyde (2000) | 13.1 | 7 | 87 | 9496 | Labor Force Participation | None |
| Jaffee & Hyde (2000) | 13.1 | 7 | 87 | 9496 | Equality in Labor Force Participation | None |
| Jaffee & Hyde (2000) | 13.1 | 7 | 91 | 9697 | LDI | None |
| Jaffee & Hyde (2000) | 13.1 | 7 | 91 | 9697 | Life Expectancy | None |
| Jaffee & Hyde (2000) | 13.1 | 7 | 91 | 9697 | RDI | None |
| Jaffee & Hyde (2000) | 13.2 | 4 | 57 | 6815 | Child Mortality Rate | None |
| Jaffee & Hyde (2000) | 13.2 | 4 | 57 | 6815 | Education | None |
| Jaffee & Hyde (2000) | 13.2 | 4 | 57 | 6815 | Equality in Education | None |
| Jaffee & Hyde (2000) | 13.2 | 4 | 57 | 6815 | GDP per capita | None |
| Jaffee & Hyde (2000) | 13.2 | 4 | 57 | 6815 | GECL | Smaller |
| Jaffee & Hyde (2000) | 13.2 | 4 | 57 | 6815 | HGEI | Smaller |
| Jaffee & Hyde (2000) | 13.2 | 4 | 57 | 6815 | Labor Force Participation | None |
| Jaffee & Hyde (2000) | 13.2 | 4 | 57 | 6815 | Equality in Labor Force Participation | None |
| Jaffee & Hyde (2000) | 13.2 | 4 | 57 | 6815 | LDI | None |
| Jaffee & Hyde (2000) | 13.2 | 4 | 57 | 6815 | Life Expectancy | None |
| Jaffee & Hyde (2000) | 13.2 | 4 | 57 | 6815 | RDI | None |
| Kling et al. (1999) | 14.1 | 18 | 152 | 86483 | Child Mortality Rate | None |
| Kling et al. (1999) | 14.1 | 18 | 153 | 88206 | Education | None |
| Kling et al. (1999) | 14.1 | 18 | 153 | 88206 | Equality in Education | Possibly smaller |
| Kling et al. (1999) | 14.1 | 19 | 154 | 88326 | GDP per capita | None |
| Kling et al. (1999) | 14.1 | 19 | 154 | 88326 | GECL | None |
| Kling et al. (1999) | 14.1 | 17 | 151 | 86363 | HGEI | None |
| Kling et al. (1999) | 14.1 | 19 | 154 | 88326 | Labor Force Participation | None |
| Kling et al. (1999) | 14.1 | 19 | 154 | 88326 | Equality in Labor Force Participation | None |
| Kling et al. (1999) | 14.1 | 19 | 154 | 88326 | LDI | None |
| Kling et al. (1999) | 14.1 | 18 | 152 | 86483 | Life Expectancy | None |
| Kling et al. (1999) | 14.1 | 19 | 154 | 88326 | RDI | None |
| Leaper & Ayres (2007) | 15.1 | 7 | 39 | 2814 | Child Mortality Rate | None |
| Leaper & Ayres (2007) | 15.1 | 7 | 39 | 2814 | Education | None |
| Leaper & Ayres (2007) | 15.1 | 7 | 39 | 2814 | Equality in Education | None |
| Leaper & Ayres (2007) | 15.1 | 7 | 39 | 2814 | GDP per capita | None |
| Leaper & Ayres (2007) | 15.1 | 7 | 39 | 2814 | GECL | None |
| Leaper & Ayres (2007) | 15.1 | 7 | 39 | 2814 | HGEI | None |
| Leaper & Ayres (2007) | 15.1 | 7 | 24 | 1652 | Labor Force Participation | None |
| Leaper & Ayres (2007) | 15.1 | 7 | 24 | 1652 | Equality in Labor Force Participation | None |
| Leaper & Ayres (2007) | 15.1 | 7 | 39 | 2814 | LDI | None |
| Leaper & Ayres (2007) | 15.1 | 7 | 39 | 2814 | Life Expectancy | None |
| Leaper & Ayres (2007) | 15.1 | 7 | 39 | 2814 | RDI | None |
| Maes et al. (2019) | 16.1 | 40 | 458 | 344069 | Child Mortality Rate | Smaller |
| Maes et al. (2019) | 16.1 | 37 | 375 | 238164 | Education | None |
| Maes et al. (2019) | 16.1 | 37 | 375 | 238164 | Equality in Education | None |
| Maes et al. (2019) | 16.1 | 41 | 463 | 349650 | GDP per capita | Smaller |
| Maes et al. (2019) | 16.1 | 41 | 463 | 349650 | GECL | Smaller |
| Maes et al. (2019) | 16.1 | 24 | 206 | 78829 | HGEI | None |
| Maes et al. (2019) | 16.1 | 40 | 447 | 340406 | Labor Force Participation | Possibly larger |
| Maes et al. (2019) | 16.1 | 40 | 447 | 340406 | Equality in Labor Force Participation | None |
| Maes et al. (2019) | 16.1 | 41 | 463 | 349650 | LDI | Smaller |
| Maes et al. (2019) | 16.1 | 40 | 458 | 344069 | Life Expectancy | Smaller |
| Maes et al. (2019) | 16.1 | 41 | 463 | 349650 | RDI | Smaller |
| Miller et al. (2008) | 17.1 | 9 | 48 | 12944 | Child Mortality Rate | Possibly larger |
| Miller et al. (2008) | 17.1 | 9 | 46 | 12620 | Education | None |
| Miller et al. (2008) | 17.1 | 9 | 46 | 12620 | Equality in Education | None |
| Miller et al. (2008) | 17.1 | 10 | 49 | 13004 | GDP per capita | None |
| Miller et al. (2008) | 17.1 | 10 | 49 | 13004 | GECL | None |
| Miller et al. (2008) | 17.1 | 8 | 43 | 11695 | HGEI | None |
| Miller et al. (2008) | 17.1 | 9 | 48 | 12944 | Labor Force Participation | Possibly larger |
| Miller et al. (2008) | 17.1 | 9 | 48 | 12944 | Equality in Labor Force Participation | None |
| Miller et al. (2008) | 17.1 | 10 | 49 | 13004 | LDI | None |
| Miller et al. (2008) | 17.1 | 9 | 48 | 12944 | Life Expectancy | Possibly larger |
| Miller et al. (2008) | 17.1 | 10 | 49 | 13004 | RDI | Possibly larger |
| Nazareth et al. (2019) | 18.1 | 19 | 162 | 13923 | Child Mortality Rate | None |
| Nazareth et al. (2019) | 18.1 | 16 | 115 | 9492 | Education | None |
| Nazareth et al. (2019) | 18.1 | 16 | 115 | 9492 | Equality in Education | None |
| Nazareth et al. (2019) | 18.1 | 20 | 164 | 14070 | GDP per capita | None |
| Nazareth et al. (2019) | 18.1 | 20 | 164 | 14070 | GECL | None |
| Nazareth et al. (2019) | 18.1 | 10 | 55 | 5292 | HGEI | None |
| Nazareth et al. (2019) | 18.1 | 19 | 161 | 13878 | Labor Force Participation | None |
| Nazareth et al. (2019) | 18.1 | 19 | 161 | 13878 | Equality in Labor Force Participation | None |
| Nazareth et al. (2019) | 18.1 | 20 | 164 | 14070 | LDI | None |
| Nazareth et al. (2019) | 18.1 | 19 | 162 | 13923 | Life Expectancy | Smaller |
| Nazareth et al. (2019) | 18.1 | 20 | 164 | 14070 | RDI | None |
| Randler & Engelke (2019) | 19.1 | 36 | 164 | 165168 | Child Mortality Rate | Larger |
| Randler & Engelke (2019) | 19.1 | 23 | 92 | 69241 | Education | None |
| Randler & Engelke (2019) | 19.1 | 23 | 92 | 69241 | Equality in Education | None |
| Randler & Engelke (2019) | 19.1 | 36 | 164 | 165168 | GDP per capita | Larger |
| Randler & Engelke (2019) | 19.1 | 36 | 164 | 165168 | GECL | Larger |
| Randler & Engelke (2019) | 19.1 | 11 | 27 | 17457 | HGEI | Smaller |
| Randler & Engelke (2019) | 19.1 | 36 | 164 | 165168 | Labor Force Participation | None |
| Randler & Engelke (2019) | 19.1 | 36 | 164 | 165168 | Equality in Labor Force Participation | Larger |
| Randler & Engelke (2019) | 19.1 | 36 | 164 | 165168 | LDI | Larger |
| Randler & Engelke (2019) | 19.1 | 36 | 164 | 165168 | Life Expectancy | Larger |
| Randler & Engelke (2019) | 19.1 | 36 | 164 | 165168 | RDI | Larger |
| Balliet et al. (2011) | 2.1 | 13 | 67 | 9817 | Child Mortality Rate | None |
| Balliet et al. (2011) | 2.1 | 14 | 68 | 10072 | Education | None |
| Balliet et al. (2011) | 2.1 | 14 | 68 | 10072 | Equality in Education | None |
| Balliet et al. (2011) | 2.1 | 14 | 68 | 10072 | GDP per capita | None |
| Balliet et al. (2011) | 2.1 | 14 | 68 | 10072 | GECL | None |
| Balliet et al. (2011) | 2.1 | 7 | 48 | 6801 | HGEI | None |
| Balliet et al. (2011) | 2.1 | 13 | 52 | 7898 | Labor Force Participation | None |
| Balliet et al. (2011) | 2.1 | 13 | 52 | 7898 | Equality in Labor Force Participation | None |
| Balliet et al. (2011) | 2.1 | 14 | 68 | 10072 | LDI | None |
| Balliet et al. (2011) | 2.1 | 13 | 67 | 9817 | Life Expectancy | None |
| Balliet et al. (2011) | 2.1 | 14 | 68 | 10072 | RDI | None |
| Balliet et al. (2011) | 2.2 | 5 | 48 | 4746 | Child Mortality Rate | Possibly smaller |
| Balliet et al. (2011) | 2.2 | 5 | 48 | 4746 | Education | None |
| Balliet et al. (2011) | 2.2 | 5 | 48 | 4746 | Equality in Education | Possibly larger |
| Balliet et al. (2011) | 2.2 | 5 | 48 | 4746 | GDP per capita | Possibly smaller |
| Balliet et al. (2011) | 2.2 | 5 | 48 | 4746 | GECL | None |
| Balliet et al. (2011) | 2.2 | 4 | 44 | 4017 | HGEI | None |
| Balliet et al. (2011) | 2.2 | 4 | 13 | 1519 | Labor Force Participation | None |
| Balliet et al. (2011) | 2.2 | 4 | 13 | 1519 | Equality in Labor Force Participation | None |
| Balliet et al. (2011) | 2.2 | 5 | 48 | 4746 | LDI | Smaller |
| Balliet et al. (2011) | 2.2 | 5 | 48 | 4746 | Life Expectancy | Possibly smaller |
| Balliet et al. (2011) | 2.2 | 5 | 48 | 4746 | RDI | Possibly smaller |
| Schredl & Reinhard (2008) | 20.1 | 13 | 73 | 32884 | Child Mortality Rate | None |
| Schredl & Reinhard (2008) | 20.1 | 12 | 69 | 28960 | Education | None |
| Schredl & Reinhard (2008) | 20.1 | 12 | 69 | 28960 | Equality in Education | None |
| Schredl & Reinhard (2008) | 20.1 | 13 | 71 | 29430 | GDP per capita | Smaller |
| Schredl & Reinhard (2008) | 20.1 | 12 | 71 | 32414 | GECL | None |
| Schredl & Reinhard (2008) | 20.1 | 12 | 69 | 28960 | HGEI | None |
| Schredl & Reinhard (2008) | 20.1 | 11 | 55 | 25361 | Labor Force Participation | None |
| Schredl & Reinhard (2008) | 20.1 | 11 | 55 | 25361 | Equality in Labor Force Participation | None |
| Schredl & Reinhard (2008) | 20.1 | 12 | 71 | 32414 | LDI | None |
| Schredl & Reinhard (2008) | 20.1 | 13 | 73 | 32884 | Life Expectancy | None |
| Schredl & Reinhard (2008) | 20.1 | 13 | 71 | 29430 | RDI | Possibly smaller |
| Schredl & Reinhard (2011) | 21.1 | 21 | 95 | 156470 | Child Mortality Rate | Possibly larger |
| Schredl & Reinhard (2011) | 21.1 | 20 | 94 | 150218 | Education | None |
| Schredl & Reinhard (2011) | 21.1 | 20 | 94 | 150218 | Equality in Education | None |
| Schredl & Reinhard (2011) | 21.1 | 19 | 93 | 150149 | GDP per capita | Larger |
| Schredl & Reinhard (2011) | 21.1 | 20 | 94 | 150757 | GECL | None |
| Schredl & Reinhard (2011) | 21.1 | 18 | 75 | 131907 | HGEI | None |
| Schredl & Reinhard (2011) | 21.1 | 19 | 81 | 106374 | Labor Force Participation | None |
| Schredl & Reinhard (2011) | 21.1 | 19 | 81 | 106374 | Equality in Labor Force Participation | None |
| Schredl & Reinhard (2011) | 21.1 | 20 | 94 | 150757 | LDI | None |
| Schredl & Reinhard (2011) | 21.1 | 20 | 94 | 150757 | Life Expectancy | Larger |
| Schredl & Reinhard (2011) | 21.1 | 19 | 93 | 150149 | RDI | Larger |
| Thompson & Voyer (2014) | 22.1 | 28 | 101 | 54548 | Child Mortality Rate | None |
| Thompson & Voyer (2014) | 22.1 | 28 | 101 | 54548 | Education | None |
| Thompson & Voyer (2014) | 22.1 | 28 | 101 | 54548 | Equality in Education | Possibly smaller |
| Thompson & Voyer (2014) | 22.1 | 28 | 101 | 54548 | GDP per capita | None |
| Thompson & Voyer (2014) | 22.1 | 28 | 101 | 54548 | GECL | None |
| Thompson & Voyer (2014) | 22.1 | 22 | 64 | 35911 | HGEI | None |
| Thompson & Voyer (2014) | 22.1 | 28 | 89 | 53308 | Labor Force Participation | Larger |
| Thompson & Voyer (2014) | 22.1 | 28 | 89 | 53308 | Equality in Labor Force Participation | Possibly larger |
| Thompson & Voyer (2014) | 22.1 | 28 | 101 | 54548 | LDI | None |
| Thompson & Voyer (2014) | 22.1 | 28 | 101 | 54548 | Life Expectancy | None |
| Thompson & Voyer (2014) | 22.1 | 28 | 101 | 54548 | RDI | None |
| Vishnevsky et al. (2010) | 23.1 | 12 | 66 | 13753 | Child Mortality Rate | None |
| Vishnevsky et al. (2010) | 23.1 | 12 | 66 | 14137 | Education | None |
| Vishnevsky et al. (2010) | 23.1 | 12 | 66 | 14137 | Equality in Education | None |
| Vishnevsky et al. (2010) | 23.1 | 13 | 67 | 14325 | GDP per capita | None |
| Vishnevsky et al. (2010) | 23.1 | 13 | 67 | 14325 | GECL | None |
| Vishnevsky et al. (2010) | 23.1 | 11 | 61 | 12793 | HGEI | Possibly smaller |
| Vishnevsky et al. (2010) | 23.1 | 12 | 66 | 13753 | Labor Force Participation | None |
| Vishnevsky et al. (2010) | 23.1 | 12 | 66 | 13753 | Equality in Labor Force Participation | Possibly smaller |
| Vishnevsky et al. (2010) | 23.1 | 13 | 67 | 14325 | LDI | None |
| Vishnevsky et al. (2010) | 23.1 | 12 | 66 | 13753 | Life Expectancy | None |
| Vishnevsky et al. (2010) | 23.1 | 13 | 67 | 14325 | RDI | None |
| Voyer & Voyer (2014) | 24.1 | 8 | 65 | 150417 | Child Mortality Rate | None |
| Voyer & Voyer (2014) | 24.1 | 8 | 62 | 149894 | Education | None |
| Voyer & Voyer (2014) | 24.1 | 8 | 62 | 149894 | Equality in Education | None |
| Voyer & Voyer (2014) | 24.1 | 8 | 62 | 149894 | GDP per capita | None |
| Voyer & Voyer (2014) | 24.1 | 8 | 65 | 150417 | GECL | Smaller |
| Voyer & Voyer (2014) | 24.1 | 6 | 44 | 42228 | HGEI | None |
| Voyer & Voyer (2014) | 24.1 | 6 | 52 | 143997 | Labor Force Participation | None |
| Voyer & Voyer (2014) | 24.1 | 6 | 52 | 143997 | Equality in Labor Force Participation | None |
| Voyer & Voyer (2014) | 24.1 | 8 | 65 | 150417 | LDI | None |
| Voyer & Voyer (2014) | 24.1 | 8 | 65 | 150417 | Life Expectancy | Possibly smaller |
| Voyer & Voyer (2014) | 24.1 | 8 | 62 | 149894 | RDI | Possibly smaller |
| Voyer & Voyer (2014) | 24.2 | 19 | 175 | 396735 | Child Mortality Rate | None |
| Voyer & Voyer (2014) | 24.2 | 20 | 175 | 341118 | Education | None |
| Voyer & Voyer (2014) | 24.2 | 20 | 175 | 341118 | Equality in Education | None |
| Voyer & Voyer (2014) | 24.2 | 20 | 175 | 341118 | GDP per capita | None |
| Voyer & Voyer (2014) | 24.2 | 20 | 176 | 396826 | GECL | None |
| Voyer & Voyer (2014) | 24.2 | 12 | 133 | 240802 | HGEI | None |
| Voyer & Voyer (2014) | 24.2 | 19 | 139 | 303422 | Labor Force Participation | None |
| Voyer & Voyer (2014) | 24.2 | 19 | 139 | 303422 | Equality in Labor Force Participation | None |
| Voyer & Voyer (2014) | 24.2 | 20 | 176 | 396826 | LDI | None |
| Voyer & Voyer (2014) | 24.2 | 19 | 175 | 396735 | Life Expectancy | None |
| Voyer & Voyer (2014) | 24.2 | 20 | 175 | 341118 | RDI | None |
| Voyer & Voyer (2014) | 24.3 | 11 | 47 | 141935 | Child Mortality Rate | None |
| Voyer & Voyer (2014) | 24.3 | 11 | 44 | 141412 | Education | Larger |
| Voyer & Voyer (2014) | 24.3 | 11 | 44 | 141412 | Equality in Education | Larger |
| Voyer & Voyer (2014) | 24.3 | 11 | 44 | 141412 | GDP per capita | Larger |
| Voyer & Voyer (2014) | 24.3 | 11 | 47 | 141935 | GECL | None |
| Voyer & Voyer (2014) | 24.3 | 8 | 31 | 34924 | HGEI | None |
| Voyer & Voyer (2014) | 24.3 | 9 | 34 | 136351 | Labor Force Participation | None |
| Voyer & Voyer (2014) | 24.3 | 9 | 34 | 136351 | Equality in Labor Force Participation | None |
| Voyer & Voyer (2014) | 24.3 | 11 | 47 | 141935 | LDI | None |
| Voyer & Voyer (2014) | 24.3 | 11 | 47 | 141935 | Life Expectancy | None |
| Voyer & Voyer (2014) | 24.3 | 11 | 44 | 141412 | RDI | Possibly larger |
| Voyer et al. (1995) | 25.1 | 12 | 127 | 21126 | Child Mortality Rate | None |
| Voyer et al. (1995) | 25.1 | 13 | 128 | 21222 | Education | Possibly larger |
| Voyer et al. (1995) | 25.1 | 13 | 128 | 21222 | Equality in Education | None |
| Voyer et al. (1995) | 25.1 | 13 | 128 | 21222 | GDP per capita | None |
| Voyer et al. (1995) | 25.1 | 13 | 128 | 21222 | GECL | None |
| Voyer et al. (1995) | 25.1 | 12 | 127 | 21126 | HGEI | None |
| Voyer et al. (1995) | 25.1 | 7 | 48 | 7292 | Labor Force Participation | Possibly larger |
| Voyer et al. (1995) | 25.1 | 7 | 48 | 7292 | Equality in Labor Force Participation | None |
| Voyer et al. (1995) | 25.1 | 13 | 128 | 21222 | LDI | None |
| Voyer et al. (1995) | 25.1 | 12 | 127 | 21126 | Life Expectancy | None |
| Voyer et al. (1995) | 25.1 | 13 | 128 | 21222 | RDI | None |
| Voyer et al. (2017) | 26.1 | 16 | 53 | 7634 | Child Mortality Rate | None |
| Voyer et al. (2017) | 26.1 | 16 | 45 | 6723 | Education | Possibly smaller |
| Voyer et al. (2017) | 26.1 | 16 | 45 | 6723 | Equality in Education | None |
| Voyer et al. (2017) | 26.1 | 16 | 53 | 7634 | GDP per capita | None |
| Voyer et al. (2017) | 26.1 | 16 | 53 | 7634 | GECL | None |
| Voyer et al. (2017) | 26.1 | 7 | 17 | 3029 | HGEI | None |
| Voyer et al. (2017) | 26.1 | 16 | 53 | 7634 | Labor Force Participation | None |
| Voyer et al. (2017) | 26.1 | 16 | 53 | 7634 | Equality in Labor Force Participation | None |
| Voyer et al. (2017) | 26.1 | 16 | 53 | 7634 | LDI | None |
| Voyer et al. (2017) | 26.1 | 16 | 53 | 7634 | Life Expectancy | None |
| Voyer et al. (2017) | 26.1 | 16 | 53 | 7634 | RDI | None |
| Yang & Girgus (2018) | 27.1 | 15 | 91 | 30322 | Child Mortality Rate | Larger |
| Yang & Girgus (2018) | 27.1 | 14 | 86 | 27558 | Education | Larger |
| Yang & Girgus (2018) | 27.1 | 14 | 86 | 27558 | Equality in Education | Larger |
| Yang & Girgus (2018) | 27.1 | 15 | 91 | 30322 | GDP per capita | Larger |
| Yang & Girgus (2018) | 27.1 | 15 | 91 | 30322 | GECL | Larger |
| Yang & Girgus (2018) | 27.1 | 6 | 51 | 13763 | HGEI | Larger |
| Yang & Girgus (2018) | 27.1 | 15 | 91 | 30322 | Labor Force Participation | None |
| Yang & Girgus (2018) | 27.1 | 15 | 91 | 30322 | Equality in Labor Force Participation | Larger |
| Yang & Girgus (2018) | 27.1 | 15 | 91 | 30322 | LDI | Larger |
| Yang & Girgus (2018) | 27.1 | 15 | 91 | 30322 | Life Expectancy | None |
| Yang & Girgus (2018) | 27.1 | 15 | 91 | 30322 | RDI | Larger |
| Block et al. (2000) | 3.1 | 8 | 23 | 3017 | Child Mortality Rate | None |
| Block et al. (2000) | 3.1 | 8 | 21 | 1283 | Education | Larger |
| Block et al. (2000) | 3.1 | 8 | 21 | 1283 | Equality in Education | Larger |
| Block et al. (2000) | 3.1 | 8 | 21 | 1283 | GDP per capita | Possibly larger |
| Block et al. (2000) | 3.1 | 8 | 23 | 3017 | GECL | None |
| Block et al. (2000) | 3.1 | 7 | 20 | 1222 | HGEI | None |
| Block et al. (2000) | 3.1 | 6 | 9 | 632 | Labor Force Participation | None |
| Block et al. (2000) | 3.1 | 6 | 9 | 632 | Equality in Labor Force Participation | None |
| Block et al. (2000) | 3.1 | 8 | 23 | 3017 | LDI | Possibly larger |
| Block et al. (2000) | 3.1 | 8 | 23 | 3017 | Life Expectancy | None |
| Block et al. (2000) | 3.1 | 8 | 21 | 1283 | RDI | Possibly larger |
| Card et al. (2008) | 4.1 | 11 | 100 | 54577 | Child Mortality Rate | None |
| Card et al. (2008) | 4.1 | 11 | 100 | 54577 | Education | None |
| Card et al. (2008) | 4.1 | 11 | 100 | 54577 | Equality in Education | None |
| Card et al. (2008) | 4.1 | 11 | 100 | 54577 | GDP per capita | None |
| Card et al. (2008) | 4.1 | 11 | 100 | 54577 | GECL | None |
| Card et al. (2008) | 4.1 | 11 | 90 | 48875 | HGEI | Larger |
| Card et al. (2008) | 4.1 | 11 | 100 | 54577 | Labor Force Participation | None |
| Card et al. (2008) | 4.1 | 11 | 100 | 54577 | Equality in Labor Force Participation | None |
| Card et al. (2008) | 4.1 | 11 | 100 | 54577 | LDI | Possibly larger |
| Card et al. (2008) | 4.1 | 11 | 100 | 54577 | Life Expectancy | None |
| Card et al. (2008) | 4.1 | 11 | 100 | 54577 | RDI | None |
| Card et al. (2008) | 4.2 | 11 | 98 | 53668 | Child Mortality Rate | None |
| Card et al. (2008) | 4.2 | 11 | 98 | 53668 | Education | None |
| Card et al. (2008) | 4.2 | 11 | 98 | 53668 | Equality in Education | Possibly larger |
| Card et al. (2008) | 4.2 | 11 | 98 | 53668 | GDP per capita | Possibly larger |
| Card et al. (2008) | 4.2 | 11 | 98 | 53668 | GECL | None |
| Card et al. (2008) | 4.2 | 11 | 88 | 47966 | HGEI | None |
| Card et al. (2008) | 4.2 | 11 | 98 | 53668 | Labor Force Participation | None |
| Card et al. (2008) | 4.2 | 11 | 98 | 53668 | Equality in Labor Force Participation | Larger |
| Card et al. (2008) | 4.2 | 11 | 98 | 53668 | LDI | None |
| Card et al. (2008) | 4.2 | 11 | 98 | 53668 | Life Expectancy | None |
| Card et al. (2008) | 4.2 | 11 | 98 | 53668 | RDI | None |
| Chaplin & Aldao (2013) | 5.1 | 5 | 52 | 6445 | Child Mortality Rate | None |
| Chaplin & Aldao (2013) | 5.1 | 5 | 52 | 6445 | Education | None |
| Chaplin & Aldao (2013) | 5.1 | 5 | 52 | 6445 | Equality in Education | None |
| Chaplin & Aldao (2013) | 5.1 | 5 | 52 | 6445 | GDP per capita | None |
| Chaplin & Aldao (2013) | 5.1 | 5 | 52 | 6445 | GECL | Possibly larger |
| Chaplin & Aldao (2013) | 5.1 | 3 | 32 | 3532 | HGEI | None |
| Chaplin & Aldao (2013) | 5.1 | 5 | 52 | 6445 | Labor Force Participation | None |
| Chaplin & Aldao (2013) | 5.1 | 5 | 52 | 6445 | Equality in Labor Force Participation | Possibly larger |
| Chaplin & Aldao (2013) | 5.1 | 5 | 52 | 6445 | LDI | None |
| Chaplin & Aldao (2013) | 5.1 | 5 | 52 | 6445 | Life Expectancy | None |
| Chaplin & Aldao (2013) | 5.1 | 5 | 52 | 6445 | RDI | None |
| Chaplin & Aldao (2013) | 5.2 | 6 | 70 | 8524 | Child Mortality Rate | None |
| Chaplin & Aldao (2013) | 5.2 | 6 | 70 | 8524 | Education | None |
| Chaplin & Aldao (2013) | 5.2 | 6 | 70 | 8524 | Equality in Education | None |
| Chaplin & Aldao (2013) | 5.2 | 6 | 70 | 8524 | GDP per capita | None |
| Chaplin & Aldao (2013) | 5.2 | 6 | 70 | 8524 | GECL | None |
| Chaplin & Aldao (2013) | 5.2 | 4 | 48 | 5691 | HGEI | None |
| Chaplin & Aldao (2013) | 5.2 | 6 | 70 | 8524 | Labor Force Participation | None |
| Chaplin & Aldao (2013) | 5.2 | 6 | 70 | 8524 | Equality in Labor Force Participation | None |
| Chaplin & Aldao (2013) | 5.2 | 6 | 70 | 8524 | LDI | None |
| Chaplin & Aldao (2013) | 5.2 | 6 | 70 | 8524 | Life Expectancy | None |
| Chaplin & Aldao (2013) | 5.2 | 6 | 70 | 8524 | RDI | None |
| Chaplin & Aldao (2013) | 5.3 | 5 | 69 | 7454 | Child Mortality Rate | None |
| Chaplin & Aldao (2013) | 5.3 | 5 | 69 | 7454 | Education | Possibly larger |
| Chaplin & Aldao (2013) | 5.3 | 5 | 69 | 7454 | Equality in Education | None |
| Chaplin & Aldao (2013) | 5.3 | 5 | 69 | 7454 | GDP per capita | None |
| Chaplin & Aldao (2013) | 5.3 | 5 | 69 | 7454 | GECL | None |
| Chaplin & Aldao (2013) | 5.3 | 3 | 45 | 4738 | HGEI | None |
| Chaplin & Aldao (2013) | 5.3 | 5 | 69 | 7454 | Labor Force Participation | None |
| Chaplin & Aldao (2013) | 5.3 | 5 | 69 | 7454 | Equality in Labor Force Participation | None |
| Chaplin & Aldao (2013) | 5.3 | 5 | 69 | 7454 | LDI | None |
| Chaplin & Aldao (2013) | 5.3 | 5 | 69 | 7454 | Life Expectancy | None |
| Chaplin & Aldao (2013) | 5.3 | 5 | 69 | 7454 | RDI | None |
| Chaplin & Aldao (2013) | 5.4 | 9 | 88 | 16847 | Child Mortality Rate | None |
| Chaplin & Aldao (2013) | 5.4 | 9 | 88 | 16847 | Education | Possibly smaller |
| Chaplin & Aldao (2013) | 5.4 | 9 | 88 | 16847 | Equality in Education | None |
| Chaplin & Aldao (2013) | 5.4 | 9 | 88 | 16847 | GDP per capita | Possibly smaller |
| Chaplin & Aldao (2013) | 5.4 | 9 | 88 | 16847 | GECL | Possibly smaller |
| Chaplin & Aldao (2013) | 5.4 | 8 | 63 | 13836 | HGEI | Possibly smaller |
| Chaplin & Aldao (2013) | 5.4 | 9 | 88 | 16847 | Labor Force Participation | None |
| Chaplin & Aldao (2013) | 5.4 | 9 | 88 | 16847 | Equality in Labor Force Participation | Smaller |
| Chaplin & Aldao (2013) | 5.4 | 9 | 88 | 16847 | LDI | None |
| Chaplin & Aldao (2013) | 5.4 | 9 | 88 | 16847 | Life Expectancy | Possibly smaller |
| Chaplin & Aldao (2013) | 5.4 | 9 | 88 | 16847 | RDI | Possibly smaller |
| Cross et al. (2011) | 6.1 | 21 | 150 | 97612 | Child Mortality Rate | None |
| Cross et al. (2011) | 6.1 | 22 | 152 | 100794 | Education | None |
| Cross et al. (2011) | 6.1 | 22 | 152 | 100794 | Equality in Education | Possibly larger |
| Cross et al. (2011) | 6.1 | 22 | 152 | 100794 | GDP per capita | None |
| Cross et al. (2011) | 6.1 | 22 | 152 | 100794 | GECL | None |
| Cross et al. (2011) | 6.1 | 18 | 119 | 66820 | HGEI | None |
| Cross et al. (2011) | 6.1 | 21 | 149 | 97185 | Labor Force Participation | None |
| Cross et al. (2011) | 6.1 | 21 | 149 | 97185 | Equality in Labor Force Participation | None |
| Cross et al. (2011) | 6.1 | 22 | 152 | 100794 | LDI | None |
| Cross et al. (2011) | 6.1 | 21 | 150 | 97612 | Life Expectancy | None |
| Cross et al. (2011) | 6.1 | 22 | 152 | 100794 | RDI | None |
| Cross et al. (2011) | 6.2 | 18 | 104 | 41877 | Child Mortality Rate | None |
| Cross et al. (2011) | 6.2 | 18 | 104 | 41488 | Education | Possibly smaller |
| Cross et al. (2011) | 6.2 | 18 | 104 | 41488 | Equality in Education | None |
| Cross et al. (2011) | 6.2 | 18 | 104 | 41488 | GDP per capita | None |
| Cross et al. (2011) | 6.2 | 18 | 104 | 41488 | GECL | None |
| Cross et al. (2011) | 6.2 | 16 | 81 | 28779 | HGEI | None |
| Cross et al. (2011) | 6.2 | 18 | 102 | 40842 | Labor Force Participation | None |
| Cross et al. (2011) | 6.2 | 18 | 102 | 40842 | Equality in Labor Force Participation | None |
| Cross et al. (2011) | 6.2 | 18 | 104 | 41488 | LDI | None |
| Cross et al. (2011) | 6.2 | 17 | 103 | 41286 | Life Expectancy | None |
| Cross et al. (2011) | 6.2 | 18 | 104 | 41488 | RDI | None |
| Davis et al. (1999) | 7.1 | 5 | 51 | 16631 | Child Mortality Rate | None |
| Davis et al. (1999) | 7.1 | 5 | 51 | 16631 | Education | None |
| Davis et al. (1999) | 7.1 | 5 | 51 | 16631 | Equality in Education | None |
| Davis et al. (1999) | 7.1 | 5 | 51 | 16631 | GDP per capita | None |
| Davis et al. (1999) | 7.1 | 5 | 51 | 16631 | GECL | None |
| Davis et al. (1999) | 7.1 | 5 | 51 | 16631 | HGEI | None |
| Davis et al. (1999) | 7.1 | 5 | 47 | 16102 | Labor Force Participation | None |
| Davis et al. (1999) | 7.1 | 5 | 47 | 16102 | Equality in Labor Force Participation | None |
| Davis et al. (1999) | 7.1 | 5 | 51 | 16631 | LDI | None |
| Davis et al. (1999) | 7.1 | 5 | 51 | 16631 | Life Expectancy | None |
| Davis et al. (1999) | 7.1 | 5 | 51 | 16631 | RDI | None |
| Del Giudice (2011) | 8.1 | 15 | 89 | 33902 | Child Mortality Rate | Possibly larger |
| Del Giudice (2011) | 8.1 | 15 | 89 | 33902 | Education | Smaller |
| Del Giudice (2011) | 8.1 | 15 | 89 | 33902 | Equality in Education | None |
| Del Giudice (2011) | 8.1 | 15 | 89 | 33902 | GDP per capita | Possibly smaller |
| Del Giudice (2011) | 8.1 | 15 | 89 | 33902 | GECL | None |
| Del Giudice (2011) | 8.1 | 13 | 59 | 25254 | HGEI | None |
| Del Giudice (2011) | 8.1 | 15 | 89 | 33902 | Labor Force Participation | Smaller |
| Del Giudice (2011) | 8.1 | 15 | 89 | 33902 | Equality in Labor Force Participation | Possibly smaller |
| Del Giudice (2011) | 8.1 | 15 | 89 | 33902 | LDI | None |
| Del Giudice (2011) | 8.1 | 15 | 89 | 33902 | Life Expectancy | Larger |
| Del Giudice (2011) | 8.1 | 15 | 89 | 33902 | RDI | None |
| Del Giudice (2011) | 8.2 | 15 | 88 | 33842 | Child Mortality Rate | None |
| Del Giudice (2011) | 8.2 | 15 | 88 | 33842 | Education | None |
| Del Giudice (2011) | 8.2 | 15 | 88 | 33842 | Equality in Education | None |
| Del Giudice (2011) | 8.2 | 15 | 88 | 33842 | GDP per capita | None |
| Del Giudice (2011) | 8.2 | 15 | 88 | 33842 | GECL | None |
| Del Giudice (2011) | 8.2 | 13 | 59 | 25254 | HGEI | Possibly larger |
| Del Giudice (2011) | 8.2 | 15 | 88 | 33842 | Labor Force Participation | Possibly larger |
| Del Giudice (2011) | 8.2 | 15 | 88 | 33842 | Equality in Labor Force Participation | Possibly larger |
| Del Giudice (2011) | 8.2 | 15 | 88 | 33842 | LDI | None |
| Del Giudice (2011) | 8.2 | 15 | 88 | 33842 | Life Expectancy | None |
| Del Giudice (2011) | 8.2 | 15 | 88 | 33842 | RDI | None |
| Else-Quest et al. (2006) | 9.1 | 13 | 82 | 25416 | Child Mortality Rate | Larger |
| Else-Quest et al. (2006) | 9.1 | 14 | 82 | 25462 | Education | Larger |
| Else-Quest et al. (2006) | 9.1 | 14 | 82 | 25462 | Equality in Education | None |
| Else-Quest et al. (2006) | 9.1 | 14 | 82 | 25462 | GDP per capita | Larger |
| Else-Quest et al. (2006) | 9.1 | 14 | 82 | 25462 | GECL | None |
| Else-Quest et al. (2006) | 9.1 | 13 | 82 | 25416 | HGEI | Larger |
| Else-Quest et al. (2006) | 9.1 | 13 | 82 | 25416 | Labor Force Participation | None |
| Else-Quest et al. (2006) | 9.1 | 13 | 82 | 25416 | Equality in Labor Force Participation | Possibly larger |
| Else-Quest et al. (2006) | 9.1 | 14 | 82 | 25462 | LDI | Larger |
| Else-Quest et al. (2006) | 9.1 | 13 | 82 | 25416 | Life Expectancy | Larger |
| Else-Quest et al. (2006) | 9.1 | 14 | 82 | 25462 | RDI | Larger |
| Else-Quest et al. (2006) | 9.2 | 18 | 119 | 33015 | Child Mortality Rate | Larger |
| Else-Quest et al. (2006) | 9.2 | 19 | 119 | 33061 | Education | Larger |
| Else-Quest et al. (2006) | 9.2 | 19 | 119 | 33061 | Equality in Education | None |
| Else-Quest et al. (2006) | 9.2 | 19 | 119 | 33061 | GDP per capita | Larger |
| Else-Quest et al. (2006) | 9.2 | 19 | 119 | 33061 | GECL | None |
| Else-Quest et al. (2006) | 9.2 | 18 | 119 | 33015 | HGEI | Larger |
| Else-Quest et al. (2006) | 9.2 | 18 | 119 | 33015 | Labor Force Participation | Possibly larger |
| Else-Quest et al. (2006) | 9.2 | 18 | 119 | 33015 | Equality in Labor Force Participation | Larger |
| Else-Quest et al. (2006) | 9.2 | 19 | 119 | 33061 | LDI | Larger |
| Else-Quest et al. (2006) | 9.2 | 18 | 119 | 33015 | Life Expectancy | Larger |
| Else-Quest et al. (2006) | 9.2 | 19 | 119 | 33061 | RDI | Larger |
| Else-Quest et al. (2006) | 9.3 | 17 | 100 | 31600 | Child Mortality Rate | Possibly larger |
| Else-Quest et al. (2006) | 9.3 | 18 | 100 | 31646 | Education | None |
| Else-Quest et al. (2006) | 9.3 | 18 | 100 | 31646 | Equality in Education | None |
| Else-Quest et al. (2006) | 9.3 | 18 | 100 | 31646 | GDP per capita | Possibly larger |
| Else-Quest et al. (2006) | 9.3 | 18 | 100 | 31646 | GECL | None |
| Else-Quest et al. (2006) | 9.3 | 17 | 100 | 31600 | HGEI | None |
| Else-Quest et al. (2006) | 9.3 | 17 | 100 | 31600 | Labor Force Participation | Smaller |
| Else-Quest et al. (2006) | 9.3 | 17 | 100 | 31600 | Equality in Labor Force Participation | Possibly smaller |
| Else-Quest et al. (2006) | 9.3 | 18 | 100 | 31646 | LDI | None |
| Else-Quest et al. (2006) | 9.3 | 17 | 100 | 31600 | Life Expectancy | None |
| Else-Quest et al. (2006) | 9.3 | 18 | 100 | 31646 | RDI | None |

**Table S3.** Simplified list of data used in the *Review*. See supplementary files for the full dataset.

| **Study** | **Dependent variable number** | **Countries** | **Studies** | **Subjects** | **Living conditions subcategory** | **Association** |
| --- | --- | --- | --- | --- | --- | --- |
| Archer (2006) | 28.1 | 16 | NA | 25067 | GE-Composite indicators | Smaller |
| Archer (2006) | 28.1 | 16 | NA | 25067 | GE-Composite indicators | Smaller |
| Archer (2006) | 28.1 | 16 | NA | 25067 | Other-Hofstede | Smaller |
| Archer (2006) | 28.1 | 16 | NA | 25067 | Other-Hofstede | Smaller |
| Asperholm et al., (2019) | 29.1 | 45 | 495 | 2681 | Economy-GDP | Larger |
| Asperholm et al., (2019) | 29.1 | 45 | 495 | 2681 | Education | Larger |
| Asperholm et al., (2019) | 29.1 | 45 | 495 | 2681 | GE-Education | Larger |
| Barber (2008) | 30.1 | 48 | NA | 14059 | Economy-GDP | Smaller |
| Barber (2008) | 30.1 | 48 | NA | 14059 | HD-Fertility | Smaller |
| Batz-Barbarich et al. (2018) | 31.1 | 9 regions | NA | 1001802 | GE-Composite indicators | None |
| Baumgartner et al. (2014) | 32.1 | 20 | NA | 14946 | Other-Miscellaneous | Larger |
| Bleidorn (2016) | 33.1 | 48 | NA | 985937 | Economy-GDP | Larger |
| Bleidorn (2016) | 33.1 | 48 | NA | 985937 | Economy-Other | None |
| Bleidorn (2016) | 33.1 | 48 | NA | 985937 | GE-Composite indicators | None |
| Bleidorn (2016) | 33.1 | 48 | NA | 985937 | GE-Representation | None |
| Bleidorn (2016) | 33.1 | 48 | NA | 985937 | HD-Composite indicators | Larger |
| Bleidorn (2016) | 33.1 | 48 | NA | 985937 | HD-Fertility | None |
| Bleidorn (2016) | 33.1 | 48 | NA | 985937 | Other-Hofstede | Larger |
| Bleidorn (2016) | 33.1 | 48 | NA | 985937 | Other-Hofstede | Larger |
| Bleidorn (2016) | 33.1 | 48 | NA | 985937 | Other-Hofstede | Larger |
| Bleidorn (2016) | 33.1 | 48 | NA | 985937 | Other-Hofstede | None |
| Bonsang (2017) | 34.1 | 27 | NA | 226661 | GE-Culture | Larger |
| Bonsang (2017) | 34.2 | 27 | NA | 226661 | GE-Culture | Larger |
| Bonsang (2017) | 34.3 | 26 | NA | 226661 | GE-Culture | Smaller |
| Conroy-Beam et al. (2015) | 35.1 | 33 | NA | 10153 | GE-Composite indicators | Smaller |
| Costa et al. (2001) | 36.1 | 26 | NA | 23031 | Economy-GDP | Larger |
| Costa et al. (2001) | 36.1 | 26 | NA | 23031 | GE-Education | Larger |
| Costa et al. (2001) | 36.1 | 26 | NA | 23031 | GE-Education | Larger |
| Costa et al. (2001) | 36.1 | 26 | NA | 23031 | GE-Life Expectancy | Larger |
| Costa et al. (2001) | 36.1 | 26 | NA | 23031 | HD-Fertility | Larger |
| Costa et al. (2001) | 36.1 | 26 | NA | 23031 | Other-Hofstede | Larger |
| Costa et al. (2001) | 36.1 | 26 | NA | 23031 | Other-Hofstede | None |
| Costa et al. (2001) | 36.1 | 26 | NA | 23031 | Other-Hofstede | None |
| Costa et al. (2001) | 36.1 | 26 | NA | 23031 | Other-Hofstede | None |
| Day et al. (2011) | 37.1 | 29 | NA | 33018 | GE-Composite indicators | None |
| Day et al. (2011) | 37.1 | 29 | NA | 33018 | Other-Miscellaneous | Smaller |
| Dickerson et al. (2015) | 38.1 | 19 | NA | 48306 | Economy-GDP | Smaller |
| Dickerson et al. (2015) | 38.1 | 19 | NA | 48306 | GE-Life Expectancy | None |
| Dickerson et al. (2015) | 38.1 | 19 | NA | 48306 | HD-Fertility | Smaller |
| Eagly & Wood (1999) | 39.1 | 36 | NA | ≈9474 | GE-Composite indicators | None |
| Eagly & Wood (1999) | 39.1 | 33 | NA | ≈9474 | GE-Composite indicators | Smaller |
| Eagly & Wood (1999) | 39.1 | 35 | NA | ≈9474 | GE-Composite indicators | Smaller |
| Eagly & Wood (1999) | 39.1 | 34 | NA | ≈9474 | GE-Composite indicators | Smaller |
| Eagly & Wood (1999) | 39.2 | 33 | NA | ≈9474 | GE-Composite indicators | Smaller |
| Eagly & Wood (1999) | 39.2 | 35 | NA | ≈9474 | GE-Composite indicators | Smaller |
| Eagly & Wood (1999) | 39.2 | 34 | NA | ≈9474 | GE-Composite indicators | Smaller |
| Eagly & Wood (1999) | 39.2 | 36 | NA | ≈9474 | GE-Composite indicators | Smaller |
| Eagly & Wood (1999) | 39.3 | 33 | NA | ≈9474 | GE-Composite indicators | None |
| Eagly & Wood (1999) | 39.3 | 35 | NA | ≈9474 | GE-Composite indicators | None |
| Eagly & Wood (1999) | 39.3 | 34 | NA | ≈9474 | GE-Composite indicators | None |
| Eagly & Wood (1999) | 39.3 | 36 | NA | ≈9474 | GE-Composite indicators | None |
| Ebbeler et al. (2017) | 40.1 | 34 | NA | 8219 | GE-Composite indicators | Smaller |
| Ebbeler et al. (2017) | 40.1 | 34 | NA | 8219 | GE-Composite indicators | Smaller |
| Ebbeler et al. (2017) | 40.1 | 34 | NA | 8219 | HD-Composite indicators | Smaller |
| Ebbeler et al. (2017) | 40.1 | 34 | NA | 8219 | Other-Hofstede | Smaller |
| Ebbeler et al. (2017) | 40.1 | 34 | NA | 8219 | Other-Hofstede | Smaller |
| Else-Quest et al. (2010) | 41.1 | 39 | NA | 273883 | Education | None |
| Else-Quest et al. (2010) | 41.1 | 39 | NA | 273883 | Education | None |
| Else-Quest et al. (2010) | 41.1 | 39 | NA | 273883 | Education | None |
| Else-Quest et al. (2010) | 41.1 | 41 | NA | 273883 | GE-Composite indicators | Larger |
| Else-Quest et al. (2010) | 41.1 | 41 | NA | 273883 | GE-Composite indicators | Larger |
| Else-Quest et al. (2010) | 41.1 | 41 | NA | 273883 | GE-Composite indicators | None |
| Else-Quest et al. (2010) | 41.1 | 41 | NA | 273883 | GE-Composite indicators | None |
| Else-Quest et al. (2010) | 41.1 | 38 | NA | 273883 | GE-Economy | None |
| Else-Quest et al. (2010) | 41.1 | 38 | NA | 273883 | GE-Representation | Larger |
| Else-Quest et al. (2010) | 41.1 | 35 | NA | 273883 | GE-Representation | None |
| Else-Quest et al. (2010) | 41.1 | 33 | NA | 273883 | GE-Representation | Smaller |
| Else-Quest et al. (2010) | 41.2 | 39 | NA | 273883 | Education | Larger |
| Else-Quest et al. (2010) | 41.2 | 39 | NA | 273883 | Education | None |
| Else-Quest et al. (2010) | 41.2 | 39 | NA | 273883 | Education | None |
| Else-Quest et al. (2010) | 41.2 | 41 | NA | 273883 | GE-Composite indicators | Larger |
| Else-Quest et al. (2010) | 41.2 | 41 | NA | 273883 | GE-Composite indicators | None |
| Else-Quest et al. (2010) | 41.2 | 41 | NA | 273883 | GE-Composite indicators | None |
| Else-Quest et al. (2010) | 41.2 | 41 | NA | 273883 | GE-Composite indicators | None |
| Else-Quest et al. (2010) | 41.2 | 38 | NA | 273883 | GE-Economy | None |
| Else-Quest et al. (2010) | 41.2 | 38 | NA | 273883 | GE-Representation | Larger |
| Else-Quest et al. (2010) | 41.2 | 35 | NA | 273883 | GE-Representation | None |
| Else-Quest et al. (2010) | 41.2 | 33 | NA | 273883 | GE-Representation | Smaller |
| Else-Quest et al. (2010) | 41.3 | 39 | NA | 273883 | Education | None |
| Else-Quest et al. (2010) | 41.3 | 39 | NA | 273883 | Education | None |
| Else-Quest et al. (2010) | 41.3 | 39 | NA | 273883 | Education | Smaller |
| Else-Quest et al. (2010) | 41.3 | 41 | NA | 273883 | GE-Composite indicators | Larger |
| Else-Quest et al. (2010) | 41.3 | 41 | NA | 273883 | GE-Composite indicators | None |
| Else-Quest et al. (2010) | 41.3 | 41 | NA | 273883 | GE-Composite indicators | None |
| Else-Quest et al. (2010) | 41.3 | 41 | NA | 273883 | GE-Composite indicators | None |
| Else-Quest et al. (2010) | 41.3 | 38 | NA | 273883 | GE-Economy | None |
| Else-Quest et al. (2010) | 41.3 | 38 | NA | 273883 | GE-Representation | Larger |
| Else-Quest et al. (2010) | 41.3 | 35 | NA | 273883 | GE-Representation | None |
| Else-Quest et al. (2010) | 41.3 | 33 | NA | 273883 | GE-Representation | Smaller |
| Else-Quest et al. (2010) | 41.4 | 39 | NA | 273883 | Education | None |
| Else-Quest et al. (2010) | 41.4 | 39 | NA | 273883 | Education | None |
| Else-Quest et al. (2010) | 41.4 | 39 | NA | 273883 | Education | None |
| Else-Quest et al. (2010) | 41.4 | 41 | NA | 273883 | GE-Composite indicators | Larger |
| Else-Quest et al. (2010) | 41.4 | 41 | NA | 273883 | GE-Composite indicators | Larger |
| Else-Quest et al. (2010) | 41.4 | 41 | NA | 273883 | GE-Composite indicators | None |
| Else-Quest et al. (2010) | 41.4 | 41 | NA | 273883 | GE-Composite indicators | None |
| Else-Quest et al. (2010) | 41.4 | 38 | NA | 273883 | GE-Economy | None |
| Else-Quest et al. (2010) | 41.4 | 38 | NA | 273883 | GE-Representation | Larger |
| Else-Quest et al. (2010) | 41.4 | 35 | NA | 273883 | GE-Representation | None |
| Else-Quest et al. (2010) | 41.4 | 33 | NA | 273883 | GE-Representation | Smaller |
| Else-Quest et al. (2010) | 41.5 | 46 | NA | 219612 | Education | None |
| Else-Quest et al. (2010) | 41.5 | 46 | NA | 219612 | Education | Smaller |
| Else-Quest et al. (2010) | 41.5 | 46 | NA | 219612 | Education | Smaller |
| Else-Quest et al. (2010) | 41.5 | 46 | NA | 219612 | GE-Composite indicators | None |
| Else-Quest et al. (2010) | 41.5 | 46 | NA | 219612 | GE-Composite indicators | None |
| Else-Quest et al. (2010) | 41.5 | 46 | NA | 219612 | GE-Composite indicators | None |
| Else-Quest et al. (2010) | 41.5 | 46 | NA | 219612 | GE-Composite indicators | None |
| Else-Quest et al. (2010) | 41.5 | 46 | NA | 219612 | GE-Economy | None |
| Else-Quest et al. (2010) | 41.5 | 46 | NA | 219612 | GE-Representation | None |
| Else-Quest et al. (2010) | 41.5 | 46 | NA | 219612 | GE-Representation | Smaller |
| Else-Quest et al. (2010) | 41.5 | 46 | NA | 219612 | GE-Representation | Smaller |
| Else-Quest et al. (2010) | 41.6 | 39 | NA | 273883 | Education | None |
| Else-Quest et al. (2010) | 41.6 | 39 | NA | 273883 | Education | None |
| Else-Quest et al. (2010) | 41.6 | 39 | NA | 273883 | Education | None |
| Else-Quest et al. (2010) | 41.6 | 41 | NA | 273883 | GE-Composite indicators | Larger |
| Else-Quest et al. (2010) | 41.6 | 41 | NA | 273883 | GE-Composite indicators | Larger |
| Else-Quest et al. (2010) | 41.6 | 41 | NA | 273883 | GE-Composite indicators | None |
| Else-Quest et al. (2010) | 41.6 | 41 | NA | 273883 | GE-Composite indicators | None |
| Else-Quest et al. (2010) | 41.6 | 38 | NA | 273883 | GE-Economy | None |
| Else-Quest et al. (2010) | 41.6 | 38 | NA | 273883 | GE-Representation | Larger |
| Else-Quest et al. (2010) | 41.6 | 35 | NA | 273883 | GE-Representation | None |
| Else-Quest et al. (2010) | 41.6 | 33 | NA | 273883 | GE-Representation | Smaller |
| Else-Quest et al. (2010) | 41.7 | 46 | NA | 219612 | Education | None |
| Else-Quest et al. (2010) | 41.7 | 46 | NA | 219612 | Education | None |
| Else-Quest et al. (2010) | 41.7 | 46 | NA | 219612 | Education | Smaller |
| Else-Quest et al. (2010) | 41.7 | 46 | NA | 219612 | GE-Composite indicators | Larger |
| Else-Quest et al. (2010) | 41.7 | 46 | NA | 219612 | GE-Composite indicators | None |
| Else-Quest et al. (2010) | 41.7 | 46 | NA | 219612 | GE-Composite indicators | None |
| Else-Quest et al. (2010) | 41.7 | 46 | NA | 219612 | GE-Composite indicators | None |
| Else-Quest et al. (2010) | 41.7 | 46 | NA | 219612 | GE-Economy | None |
| Else-Quest et al. (2010) | 41.7 | 46 | NA | 219612 | GE-Representation | None |
| Else-Quest et al. (2010) | 41.7 | 46 | NA | 219612 | GE-Representation | Smaller |
| Else-Quest et al. (2010) | 41.7 | 46 | NA | 219612 | GE-Representation | Smaller |
| Else-Quest et al. (2010) | 41.8 | 39 | NA | 273883 | Education | None |
| Else-Quest et al. (2010) | 41.8 | 39 | NA | 273883 | Education | None |
| Else-Quest et al. (2010) | 41.8 | 39 | NA | 273883 | Education | None |
| Else-Quest et al. (2010) | 41.8 | 41 | NA | 273883 | GE-Composite indicators | Smaller |
| Else-Quest et al. (2010) | 41.8 | 41 | NA | 273883 | GE-Composite indicators | Smaller |
| Else-Quest et al. (2010) | 41.8 | 41 | NA | 273883 | GE-Composite indicators | Smaller |
| Else-Quest et al. (2010) | 41.8 | 41 | NA | 273883 | GE-Composite indicators | Smaller |
| Else-Quest et al. (2010) | 41.8 | 38 | NA | 273883 | GE-Economy | Smaller |
| Else-Quest et al. (2010) | 41.8 | 35 | NA | 273883 | GE-Representation | None |
| Else-Quest et al. (2010) | 41.8 | 38 | NA | 273883 | GE-Representation | Smaller |
| Else-Quest et al. (2010) | 41.8 | 33 | NA | 273883 | GE-Representation | Smaller |
| Else-Quest et al. (2010) | 41.9 | 46 | NA | 219612 | Education | Larger |
| Else-Quest et al. (2010) | 41.9 | 46 | NA | 219612 | Education | Larger |
| Else-Quest et al. (2010) | 41.9 | 46 | NA | 219612 | Education | Larger |
| Else-Quest et al. (2010) | 41.9 | 49 | NA | 219612 | GE-Composite indicators | None |
| Else-Quest et al. (2010) | 41.9 | 49 | NA | 219612 | GE-Composite indicators | None |
| Else-Quest et al. (2010) | 41.9 | 49 | NA | 219612 | GE-Composite indicators | None |
| Else-Quest et al. (2010) | 41.9 | 49 | NA | 219612 | GE-Composite indicators | None |
| Else-Quest et al. (2010) | 41.9 | 46 | NA | 219612 | GE-Economy | None |
| Else-Quest et al. (2010) | 41.9 | 46 | NA | 219612 | GE-Representation | None |
| Else-Quest et al. (2010) | 41.9 | 46 | NA | 219612 | GE-Representation | None |
| Else-Quest et al. (2010) | 41.9 | 46 | NA | 219612 | GE-Representation | None |
| Falk & Hermle (2018) | 42.1 | 76 | NA | 80000 | Economy-GDP | Larger |
| Falk & Hermle (2018) | 42.1 | 76 | NA | 80000 | GE-Composite indicators | Larger |
| Falk & Hermle (2018) | 42.2 | 76 | NA | 80000 | Economy-GDP | Larger |
| Falk & Hermle (2018) | 42.2 | 76 | NA | 80000 | GE-Composite indicators | Larger |
| Falk & Hermle (2018) | 42.3 | 76 | NA | 80000 | Economy-GDP | Larger |
| Falk & Hermle (2018) | 42.3 | 76 | NA | 80000 | GE-Composite indicators | Larger |
| Falk & Hermle (2018) | 42.4 | 76 | NA | 80000 | Economy-GDP | Larger |
| Falk & Hermle (2018) | 42.4 | 76 | NA | 80000 | GE-Composite indicators | Larger |
| Falk & Hermle (2018) | 42.5 | 76 | NA | 80000 | Economy-GDP | Larger |
| Falk & Hermle (2018) | 42.5 | 76 | NA | 80000 | GE-Composite indicators | Larger |
| Falk & Hermle (2018) | 42.6 | 76 | NA | 80000 | Economy-GDP | Larger |
| Falk & Hermle (2018) | 42.6 | 76 | NA | 80000 | GE-Composite indicators | Larger |
| Fischer et al. (2004) | 43.1 | 37 | NA | ≈3000 | GE-Composite indicators | Smaller |
| Fischer et al. (2004) | 43.2 | 37 | NA | ≈3000 | GE-Composite indicators | None |
| Fischer et al. (2004) | 43.3 | 37 | NA | ≈3000 | GE-Composite indicators | None |
| Fischer et al. (2004) | 43.4 | 37 | NA | ≈3000 | GE-Composite indicators | Larger |
| Giolla & Kajonius (2019) | 44.1 | 22 | NA | 130602 | GE-Composite indicators | Larger |
| Goldman & Penner (2016) | 45.1 | 34 | NA | 219770 | GE-Composite indicators | Larger |
| Goldman & Penner (2016) | 45.2 | 34 | NA | 219770 | GE-Composite indicators | None |
| Hopcroft & Burr Bradley (2007) | 46.1 | 29 | NA | 27311 | GE-Composite indicators | Larger |
| Hopcroft & McLaughlin (2012) | 47.1 | 23 | NA | 27967 | GE-Composite indicators | Larger |
| Kaiser (2019) | 48.1 | 50 | NA | 867782 | GE-Composite indicators | Larger |
| Kaiser (2019) | 48.1 | 50 | NA | 867782 | HD-Composite indicators | Larger |
| Kaiser (2019) | 48.1 | 50 | NA | 867782 | HD-Health | Larger |
| Kaiser (2019) | 48.1 | 50 | NA | 867782 | HD-Health | Larger |
| Kaiser (2019) | 48.1 | 50 | NA | 867782 | HD-Health | Larger |
| Kaiser (2019) | 48.1 | 50 | NA | 867782 | HD-Health | Larger |
| Kaiser (2019) | 48.1 | 50 | NA | 867782 | Other-Hofstede | Larger |
| Lauer et al. (2019) | 49.1 | NA | 128 | 30613 | Education | None |
| Lauer et al. (2019) | 49.1 | NA | 128 | 30613 | GE-Composite indicators | None |
| Lauer et al. (2019) | 49.1 | NA | 128 | 30613 | HD-Composite indicators | None |
| Lippa (2007) | 50.1 | 47 | NA | ≈218195 | GE-Composite indicators | None |
| Lippa (2007) | 50.1 | 53 | NA | ≈218195 | GE-Composite indicators | None |
| Lippa (2007) | 50.2 | 47 | NA | ≈218195 | GE-Composite indicators | Smaller |
| Lippa (2007) | 50.2 | 53 | NA | ≈218195 | GE-Composite indicators | Smaller |
| Lippa (2007) | 50.3 | 47 | NA | ≈218195 | GE-Composite indicators | Smaller |
| Lippa (2007) | 50.3 | 53 | NA | ≈218195 | GE-Composite indicators | Smaller |
| Lippa (2008) | 51.1 | 52 | NA | ≈255000 | Economy-GDP | None |
| Lippa (2008) | 51.1 | 47 | NA | ≈255000 | GE-Composite indicators | Larger |
| Lippa (2008) | 51.1 | 53 | NA | ≈255000 | GE-Composite indicators | Larger |
| Lippa (2008) | 51.1 | 53 | NA | ≈255000 | HD-Health | Larger |
| Lippa (2008) | 51.2 | 52 | NA | ≈255000 | Economy-GDP | None |
| Lippa (2008) | 51.2 | 47 | NA | ≈255000 | GE-Composite indicators | None |
| Lippa (2008) | 51.2 | 53 | NA | ≈255000 | GE-Composite indicators | None |
| Lippa (2008) | 51.2 | 53 | NA | ≈255000 | HD-Health | None |
| Lippa (2008) | 51.3 | 52 | NA | ≈255000 | Economy-GDP | None |
| Lippa (2008) | 51.3 | 47 | NA | ≈255000 | GE-Composite indicators | None |
| Lippa (2008) | 51.3 | 53 | NA | ≈255000 | GE-Composite indicators | None |
| Lippa (2008) | 51.3 | 53 | NA | ≈255000 | HD-Health | None |
| Lippa (2009) | 52.1 | 52 | NA | ≈200000 | Economy-GDP | None |
| Lippa (2009) | 52.1 | 47 | NA | ≈200000 | GE-Composite indicators | None |
| Lippa (2009) | 52.1 | 53 | NA | ≈200000 | GE-Composite indicators | None |
| Lippa (2009) | 52.1 | 53 | NA | ≈200000 | HD-Fertility | Smaller |
| Lippa (2009) | 52.1 | 45 | NA | ≈200000 | HD-Fertility | Smaller |
| Lippa (2009) | 52.1 | 53 | NA | ≈200000 | HD-Health | None |
| Lippa (2009) | 52.1 | 53 | NA | ≈200000 | HD-Health | None |
| Lippa (2009) | 52.2 | 52 | NA | ≈200000 | Economy-GDP | Smaller |
| Lippa (2009) | 52.2 | 47 | NA | ≈200000 | GE-Composite indicators | Smaller |
| Lippa (2009) | 52.2 | 53 | NA | ≈200000 | GE-Composite indicators | Smaller |
| Lippa (2009) | 52.2 | 53 | NA | ≈200000 | HD-Fertility | Smaller |
| Lippa (2009) | 52.2 | 45 | NA | ≈200000 | HD-Fertility | Smaller |
| Lippa (2009) | 52.2 | 53 | NA | ≈200000 | HD-Health | Smaller |
| Lippa (2009) | 52.2 | 53 | NA | ≈200000 | HD-Health | Smaller |
| Lippa et al. (2010) | 53.1 | 52 | NA | ≈200000 | Economy-GDP | Larger |
| Lippa et al. (2010) | 53.1 | 47 | NA | ≈200000 | GE-Composite indicators | Larger |
| Lippa et al. (2010) | 53.1 | 53 | NA | ≈200000 | GE-Composite indicators | Larger |
| Lippa et al. (2010) | 53.1 | 53 | NA | ≈200000 | HD-Health | Larger |
| Lippa et al. (2010) | 53.2 | 52 | NA | ≈200000 | Economy-GDP | Larger |
| Lippa et al. (2010) | 53.2 | 47 | NA | ≈200000 | GE-Composite indicators | Larger |
| Lippa et al. (2010) | 53.2 | 53 | NA | ≈200000 | GE-Composite indicators | Larger |
| Lippa et al. (2010) | 53.2 | 53 | NA | ≈200000 | HD-Health | Larger |
| Marks (2008) | 54.1 | 31 | NA | ≈172000 | Economy-GDP | None |
| Marks (2008) | 54.1 | 31 | NA | ≈172000 | Economy-Other | None |
| Marks (2008) | 54.1 | 31 | NA | ≈172000 | Economy-Other | None |
| Marks (2008) | 54.1 | 31 | NA | ≈172000 | Education | Smaller |
| Marks (2008) | 54.1 | 31 | NA | ≈172000 | GE-Economy | None |
| Marks (2008) | 54.1 | 31 | NA | ≈172000 | GE-Representation | Smaller |
| Marks (2008) | 54.2 | 31 | NA | ≈172000 | Economy-GDP | None |
| Marks (2008) | 54.2 | 31 | NA | ≈172000 | Economy-Other | Larger |
| Marks (2008) | 54.2 | 31 | NA | ≈172000 | Economy-Other | Larger |
| Marks (2008) | 54.2 | 31 | NA | ≈172000 | Education | None |
| Marks (2008) | 54.2 | 31 | NA | ≈172000 | GE-Economy | None |
| Marks (2008) | 54.2 | 31 | NA | ≈172000 | GE-Representation | Larger |
| McDuff et al. (2017) | 55.1 | 12 | NA | 740984 | Other-Hofstede | Larger |
| McDuff et al. (2017) | 55.2 | 12 | NA | 740984 | Other-Hofstede | None |
| Merten (2005) | 56.1 | 13 | NA | 33827 | GE-Composite indicators | Larger |
| Merten (2005) | 56.1 | 13 | NA | 33827 | Other-Hofstede | None |
| Merten (2005) | 56.1 | 13 | NA | 33827 | Other-Hofstede | None |
| Nivette et al. (2019) | 57.1 | 52 | NA | 222547 | Economy-Other | Larger |
| Nivette et al. (2019) | 57.1 | 52 | NA | 222547 | GE-Composite indicators | Larger |
| Nivette et al. (2019) | 57.1 | 52 | NA | 222547 | Other-Miscellaneous | None |
| Petersen & Hyde (2010) | 58.1 | 87 | 109 | 1419807 | GE-Composite indicators | None |
| Petersen & Hyde (2010) | 58.10 | 87 | 23 | 1419807 | GE-Composite indicators | None |
| Petersen & Hyde (2010) | 58.11 | 87 | 70 | 1419807 | GE-Composite indicators | None |
| Petersen & Hyde (2010) | 58.2 | 87 | 67 | 1419807 | GE-Composite indicators | Smaller |
| Petersen & Hyde (2010) | 58.3 | 87 | 53 | 1419807 | GE-Composite indicators | Smaller |
| Petersen & Hyde (2010) | 58.4 | 87 | 23 | 1419807 | GE-Composite indicators | None |
| Petersen & Hyde (2010) | 58.5 | 87 | 43 | 1419807 | GE-Composite indicators | None |
| Petersen & Hyde (2010) | 58.6 | 87 | 335 | 1419807 | GE-Composite indicators | Smaller |
| Petersen & Hyde (2010) | 58.7 | 87 | 61 | 1419807 | GE-Composite indicators | Smaller |
| Petersen & Hyde (2010) | 58.8 | 87 | 209 | 1419807 | GE-Composite indicators | None |
| Petersen & Hyde (2010) | 58.9 | 87 | 78 | 1419807 | GE-Composite indicators | Smaller |
| Salk et al. (2017) | 59.1 | 75 | 65 | 1716195 | Economy-GDP | Larger |
| Salk et al. (2017) | 59.1 | 75 | 65 | 1716195 | Economy-Other | None |
| Salk et al. (2017) | 59.1 | 75 | 65 | 1716195 | Education | Larger |
| Salk et al. (2017) | 59.1 | 75 | 65 | 1716195 | GE-Representation | None |
| Salk et al. (2017) | 59.1 | 75 | 65 | 1716195 | HD-Fertility | Larger |
| Salk et al. (2017) | 59.2 | 53 | 95 | 1992064 | Economy-GDP | None |
| Salk et al. (2017) | 59.2 | 53 | 95 | 1992064 | Economy-Other | Larger |
| Salk et al. (2017) | 59.2 | 53 | 95 | 1992064 | Education | None |
| Salk et al. (2017) | 59.2 | 53 | 95 | 1992064 | GE-Representation | None |
| Salk et al. (2017) | 59.2 | 53 | 95 | 1992064 | HD-Fertility | None |
| Schmitt (2008) | 60.1 | 54 | NA | 17804 | Economy-GDP | Larger |
| Schmitt (2008) | 60.1 | 54 | NA | 17804 | HD-Composite indicators | Larger |
| Schmitt (2008) | 60.1 | 54 | NA | 17804 | HD-Fertility | Larger |
| Schmitt (2008) | 60.1 | 54 | NA | 17804 | HD-Health | Larger |
| Schmitt (2008) | 60.1 | 56 | NA | 17804 | HD-Health | Larger |
| Schmitt (2008) | 60.1 | 54 | NA | 17804 | HD-Health | Larger |
| Schmitt (2008) | 60.1 | 54 | NA | 17804 | HD-Health | Larger |
| Schmitt (2008) | 60.1 | 26 | NA | 17804 | HD-Health | None |
| Schmitt (2008) | 60.1 | 50 | NA | 17804 | HD-Health | None |
| Schmitt (2008) | 60.1 | 42 | NA | 17804 | Other-Miscellaneous | None |
| Schmitt (2008) | 60.1 | 29 | NA | 17804 | Other-Miscellaneous | None |
| Schmitt (2005) | 61.1 | 46 | NA | 14059 | Economy-GDP | Smaller |
| Schmitt (2005) | 61.1 | 45 | NA | 14059 | GE-Composite indicators | None |
| Schmitt (2005) | 61.1 | 34 | NA | 14059 | GE-Composite indicators | Smaller |
| Schmitt (2005) | 61.1 | 8-14 | NA | 14059 | GE-Culture | None |
| Schmitt (2005) | 61.1 | 27 | NA | 14059 | GE-Economy | None |
| Schmitt (2005) | 61.1 | 46 | NA | 14059 | GE-Representation | Smaller |
| Schmitt (2005) | 61.1 | 46 | NA | 14059 | GE-Representation | Smaller |
| Schmitt (2005) | 61.1 | 29 | NA | 14059 | GE-Representation | Smaller |
| Schmitt (2005) | 61.1 | 46 | NA | ≈14059 | HD-Composite indicators | None |
| Schmitt (2005) | 61.1 | 41 | NA | 14059 | HD-Fertility | Smaller |
| Schmitt (2005) | 61.1 | 46 | NA | 14059 | HD-Health | None |
| Schmitt (2005) | 61.1 | 20 | NA | 14059 | HD-Health | None |
| Schmitt (2005) | 61.1 | 47 | NA | 14059 | HD-Health | None |
| Schmitt (2005) | 61.1 | 44 | NA | 14059 | HD-Health | None |
| Schmitt et al. (2003) | 62.1 | 53 | NA | ≈17804 | GE-Composite indicators | Larger |
| Schmitt et al. (2003) | 62.1 | 36 | NA | ≈17804 | GE-Composite indicators | None |
| Schmitt et al. (2003) | 62.1 | 54 | NA | ≈17804 | HD-Composite indicators | Larger |
| Schmitt et al. (2003) | 62.1 | 54 | NA | ≈17804 | HD-Fertility | Larger |
| Schmitt et al. (2003) | 62.1 | 54 | NA | ≈17804 | HD-Health | Larger |
| Schmitt et al. (2003) | 62.1 | 54 | NA | ≈17804 | HD-Health | Larger |
| Schmitt et al. (2003) | 62.1 | 20 | NA | ≈17804 | HD-Health | None |
| Schmitt et al. (2003) | 62.1 | 49 | NA | ≈17804 | Other-Hofstede | None |
| Schmitt et al. (2008) | 63.1 | 53 | NA | ≈17637 | Economy-GDP | Larger |
| Schmitt et al. (2008) | 63.1 | 47 | NA | ≈17637 | Economy-Other | None |
| Schmitt et al. (2008) | 63.1 | 53 | NA | ≈17637 | Education | None |
| Schmitt et al. (2008) | 63.1 | 41 | NA | ≈17637 | GE-Composite indicators | Larger |
| Schmitt et al. (2008) | 63.1 | 53 | NA | ≈17637 | GE-Composite indicators | Larger |
| Schmitt et al. (2008) | 63.1 | 53 | NA | ≈17637 | GE-Economy | None |
| Schmitt et al. (2008) | 63.1 | 53 | NA | ≈17637 | GE-Education | Larger |
| Schmitt et al. (2008) | 63.1 | 53 | NA | ≈17637 | GE-Life Expectancy | Larger |
| Schmitt et al. (2008) | 63.1 | 43 | NA | ≈17637 | GE-Representation | Larger |
| Schmitt et al. (2008) | 63.1 | 53 | NA | ≈17637 | HD-Composite indicators | Larger |
| Schmitt et al. (2008) | 63.1 | 53 | NA | ≈17637 | HD-Health | Larger |
| Schmitt et al. (2008) | 63.1 | 45 | NA | ≈17637 | Other-Hofstede | Larger |
| Schmitt et al. (2008) | 63.1 | 45 | NA | ≈17637 | Other-Hofstede | None |
| Schmitt et al. (2008) | 63.1 | 45 | NA | ≈17637 | Other-Hofstede | None |
| Schmitt et al. (2008) | 63.1 | 45 | NA | ≈17637 | Other-Hofstede | None |
| Schmitt et al. (2008) | 63.1 | 46 | NA | ≈17637 | Other-Miscellaneous | Larger |
| Schwartz & Rubel-Lifschitz (2009) | 64.1 | 68 | NA | 25968 | GE-Composite indicators | Larger |
| Schwartz & Rubel-Lifschitz (2009) | 64.10 | 25 | NA | 42355 | GE-Composite indicators | Larger |
| Schwartz & Rubel-Lifschitz (2009) | 64.11 | 68 | NA | 25968 | GE-Composite indicators | None |
| Schwartz & Rubel-Lifschitz (2009) | 64.12 | 25 | NA | 42355 | GE-Composite indicators | None |
| Schwartz & Rubel-Lifschitz (2009) | 64.13 | 68 | NA | 25968 | GE-Composite indicators | Smaller |
| Schwartz & Rubel-Lifschitz (2009) | 64.14 | 25 | NA | 42355 | GE-Composite indicators | None |
| Schwartz & Rubel-Lifschitz (2009) | 64.15 | 68 | NA | 25968 | GE-Composite indicators | Larger |
| Schwartz & Rubel-Lifschitz (2009) | 64.16 | 25 | NA | 42355 | GE-Composite indicators | Larger |
| Schwartz & Rubel-Lifschitz (2009) | 64.17 | 68 | NA | 25968 | GE-Composite indicators | Larger |
| Schwartz & Rubel-Lifschitz (2009) | 64.18 | 25 | NA | 42355 | GE-Composite indicators | Larger |
| Schwartz & Rubel-Lifschitz (2009) | 64.19 | 68 | NA | 25968 | GE-Composite indicators | Larger |
| Schwartz & Rubel-Lifschitz (2009) | 64.2 | 25 | NA | 42355 | GE-Composite indicators | None |
| Schwartz & Rubel-Lifschitz (2009) | 64.20 | 25 | NA | 42355 | GE-Composite indicators | Larger |
| Schwartz & Rubel-Lifschitz (2009) | 64.3 | 68 | NA | 25968 | GE-Composite indicators | Larger |
| Schwartz & Rubel-Lifschitz (2009) | 64.4 | 25 | NA | 42355 | GE-Composite indicators | Larger |
| Schwartz & Rubel-Lifschitz (2009) | 64.5 | 68 | NA | 25968 | GE-Composite indicators | None |
| Schwartz & Rubel-Lifschitz (2009) | 64.6 | 25 | NA | 42355 | GE-Composite indicators | None |
| Schwartz & Rubel-Lifschitz (2009) | 64.7 | 68 | NA | 25968 | GE-Composite indicators | None |
| Schwartz & Rubel-Lifschitz (2009) | 64.8 | 25 | NA | 42355 | GE-Composite indicators | None |
| Schwartz & Rubel-Lifschitz (2009) | 64.9 | 68 | NA | 25968 | GE-Composite indicators | Larger |
| Seedat et al. (2009) | 65.1 | 15 | NA | 72933 | GE-Culture | Smaller |
| Shan et al. (2019) | 66.1 | 30 | 112 | 23016 | GE-Composite indicators | Larger |
| Silverman et al. (2007) | 67.1 | 40 | NA | 247516 | Economy-GDP | None |
| Silverman et al. (2007) | 67.2 | 40 | NA | 244893 | Economy-GDP | None |
| Stoet & Geary (2013) | 68.1 | 43 | NA | 227158 | Economy-Other | None |
| Stoet & Geary (2013) | 68.1 | 43 | NA | 227158 | GE-Composite indicators | Larger |
| Stoet & Geary (2013) | 68.1 | 43 | NA | 227158 | GE-Composite indicators | None |
| Stoet & Geary (2013) | 68.1 | 43 | NA | 227158 | GE-Composite indicators | None |
| Stoet & Geary (2013) | 68.1 | 43 | NA | 227158 | GE-Composite indicators | None |
| Stoet & Geary (2013) | 68.1 | 43 | NA | 227158 | HD-Composite indicators | Larger |
| Stoet & Geary (2013) | 68.2 | 42 | NA | 275369 | Economy-Other | None |
| Stoet & Geary (2013) | 68.2 | 42 | NA | 275369 | GE-Composite indicators | None |
| Stoet & Geary (2013) | 68.2 | 42 | NA | 275369 | GE-Composite indicators | None |
| Stoet & Geary (2013) | 68.2 | 42 | NA | 275369 | GE-Composite indicators | None |
| Stoet & Geary (2013) | 68.2 | 42 | NA | 275369 | GE-Composite indicators | Smaller |
| Stoet & Geary (2013) | 68.2 | 42 | NA | 275369 | HD-Composite indicators | None |
| Stoet & Geary (2013) | 68.3 | 58 | NA | 398746 | Economy-Other | None |
| Stoet & Geary (2013) | 68.3 | 58 | NA | 398746 | GE-Composite indicators | None |
| Stoet & Geary (2013) | 68.3 | 58 | NA | 398746 | GE-Composite indicators | None |
| Stoet & Geary (2013) | 68.3 | 58 | NA | 398746 | GE-Composite indicators | None |
| Stoet & Geary (2013) | 68.3 | 58 | NA | 398746 | GE-Composite indicators | None |
| Stoet & Geary (2013) | 68.3 | 58 | NA | 398746 | HD-Composite indicators | None |
| Stoet & Geary (2013) | 68.4 | 74 | NA | 515956 | Economy-Other | None |
| Stoet & Geary (2013) | 68.4 | 74 | NA | 515956 | GE-Composite indicators | None |
| Stoet & Geary (2013) | 68.4 | 74 | NA | 515956 | GE-Composite indicators | None |
| Stoet & Geary (2013) | 68.4 | 74 | NA | 515956 | GE-Composite indicators | None |
| Stoet & Geary (2013) | 68.4 | 74 | NA | 515956 | GE-Composite indicators | None |
| Stoet & Geary (2013) | 68.4 | 74 | NA | 515956 | HD-Composite indicators | None |
| Stoet & Geary (2013) | 68.5 | 43 | NA | 227158 | Economy-Other | Larger |
| Stoet & Geary (2013) | 68.5 | 43 | NA | 227158 | GE-Composite indicators | Larger |
| Stoet & Geary (2013) | 68.5 | 43 | NA | 227158 | GE-Composite indicators | None |
| Stoet & Geary (2013) | 68.5 | 43 | NA | 227158 | GE-Composite indicators | None |
| Stoet & Geary (2013) | 68.5 | 43 | NA | 227158 | GE-Composite indicators | None |
| Stoet & Geary (2013) | 68.5 | 43 | NA | 227158 | HD-Composite indicators | None |
| Stoet & Geary (2013) | 68.6 | 42 | NA | 275369 | Economy-Other | None |
| Stoet & Geary (2013) | 68.6 | 42 | NA | 275369 | GE-Composite indicators | Larger |
| Stoet & Geary (2013) | 68.6 | 42 | NA | 275369 | GE-Composite indicators | None |
| Stoet & Geary (2013) | 68.6 | 42 | NA | 275369 | GE-Composite indicators | None |
| Stoet & Geary (2013) | 68.6 | 42 | NA | 275369 | GE-Composite indicators | None |
| Stoet & Geary (2013) | 68.6 | 42 | NA | 275369 | HD-Composite indicators | None |
| Stoet & Geary (2013) | 68.7 | 58 | NA | 398746 | Economy-Other | None |
| Stoet & Geary (2013) | 68.7 | 58 | NA | 398746 | GE-Composite indicators | None |
| Stoet & Geary (2013) | 68.7 | 58 | NA | 398746 | GE-Composite indicators | None |
| Stoet & Geary (2013) | 68.7 | 58 | NA | 398746 | GE-Composite indicators | None |
| Stoet & Geary (2013) | 68.7 | 58 | NA | 398746 | GE-Composite indicators | None |
| Stoet & Geary (2013) | 68.7 | 58 | NA | 398746 | HD-Composite indicators | None |
| Stoet & Geary (2013) | 68.8 | 74 | NA | 515956 | Economy-Other | Larger |
| Stoet & Geary (2013) | 68.8 | 74 | NA | 515956 | GE-Composite indicators | None |
| Stoet & Geary (2013) | 68.8 | 74 | NA | 515956 | GE-Composite indicators | None |
| Stoet & Geary (2013) | 68.8 | 74 | NA | 515956 | GE-Composite indicators | None |
| Stoet & Geary (2013) | 68.8 | 74 | NA | 515956 | GE-Composite indicators | None |
| Stoet & Geary (2013) | 68.8 | 74 | NA | 515956 | HD-Composite indicators | None |
| Stoet & Geary (2015) | 69.1 | 41 | NA | 227158 | GE-Composite indicators | None |
| Stoet & Geary (2015) | 69.1 | 32 | NA | 227158 | GE-Composite indicators | None |
| Stoet & Geary (2015) | 69.1 | 41 | NA | 227158 | GE-Economy | Smaller |
| Stoet & Geary (2015) | 69.1 | 42 | NA | 227158 | GE-Representation | None |
| Stoet & Geary (2015) | 69.1 | 33 | NA | 227158 | GE-Representation | Smaller |
| Stoet & Geary (2015) | 69.2 | 37 | NA | 275369 | GE-Composite indicators | Smaller |
| Stoet & Geary (2015) | 69.2 | 33 | NA | 275369 | GE-Composite indicators | Smaller |
| Stoet & Geary (2015) | 69.2 | 37 | NA | 275369 | GE-Economy | Smaller |
| Stoet & Geary (2015) | 69.2 | 39 | NA | 275369 | GE-Representation | None |
| Stoet & Geary (2015) | 69.2 | 31 | NA | 275369 | GE-Representation | None |
| Stoet & Geary (2015) | 69.3 | 37 | NA | 398746 | GE-Composite indicators | None |
| Stoet & Geary (2015) | 69.3 | 43 | NA | 398746 | GE-Composite indicators | None |
| Stoet & Geary (2015) | 69.3 | 51 | NA | 398746 | GE-Economy | None |
| Stoet & Geary (2015) | 69.3 | 54 | NA | 398746 | GE-Representation | None |
| Stoet & Geary (2015) | 69.3 | 42 | NA | 398746 | GE-Representation | None |
| Stoet & Geary (2015) | 69.4 | 62 | NA | 515956 | GE-Composite indicators | Larger |
| Stoet & Geary (2015) | 69.4 | 37 | NA | 515956 | GE-Composite indicators | None |
| Stoet & Geary (2015) | 69.4 | 64 | NA | 515956 | GE-Economy | None |
| Stoet & Geary (2015) | 69.4 | 66 | NA | 515956 | GE-Representation | None |
| Stoet & Geary (2015) | 69.4 | 48 | NA | 515956 | GE-Representation | None |
| Stoet & Geary (2018) | 70.1 | 61 | NA | ≤472242 | GE-Composite indicators | Larger |
| Stoet & Geary (2018) | 70.2 | 67 | NA | ≤472242 | GE-Composite indicators | Larger |
| Stoet & Geary (2018) | 70.3 | 67 | NA | ≤472242 | GE-Composite indicators | Larger |
| Stoet & Geary (2018) | 70.4 | 62 | NA | ≤472242 | GE-Composite indicators | None |
| Su et al. (2019) | 71.1 | 34 | 101 | 204352 | Economy-GDP | Smaller |
| Su et al. (2019) | 71.1 | 34 | 101 | 204352 | GE-Economy | Smaller |
| Tao & Michalopoulos (2018) | 72.1 | 57 | NA | NA | GE-Composite indicators | Smaller |
| Tao & Michalopoulos (2018) | 72.1 | 57 | NA | NA | GE-Representation | None |
| Tao & Michalopoulos (2018) | 72.1 | 57 | NA | NA | GE-Representation | None |
| Thijs (2015) | 73.1 | 24 | NA | 18027 | GE-Composite indicators | Smaller |
| Thijs (2015) | 73.1 | 24 | NA | 18027 | GE-Economy | None |
| Van de Velde et al. (2013) | 74.1 | 25 | NA | 39891 | GE-Composite indicators | None |
| Van de Velde et al. (2013) | 74.1 | 25 | NA | 39891 | GE-Economy | None |
| Van de Velde et al. (2013) | 74.1 | 25 | NA | 39891 | GE-Economy | None |
| Van de Velde et al. (2013) | 74.1 | 25 | NA | 39891 | GE-Representation | None |
| Van Hek et al. (2017) | 75.1 | 37 | NA | 1425356 | GE-Composite indicators | None |
| Van Hek et al. (2017) | 75.1 | 37 | NA | 1425356 | HD-Composite indicators | None |
| Van Hek et al. (2017) | 75.2 | 37 | NA | 1425356 | GE-Composite indicators | None |
| Van Hek et al. (2017) | 75.2 | 37 | NA | 1425356 | HD-Composite indicators | Smaller |
| van Hemert et al. (2011) | 76.1 | 37 | NA | 5715 | GE-Composite indicators | Larger |
| van Hemert et al. (2011) | 76.1 | 28 | NA | 5715 | GE-Composite indicators | Larger |
| van Hemert et al. (2011) | 76.1 | 15 | NA | 5715 | GE-Culture | Larger |
| van Hemert et al. (2011) | 76.1 | 30 | NA | 5715 | Other-Hofstede | None |
| van Hemert et al. (2011) | 76.2 | 37 | NA | 5715 | GE-Composite indicators | Larger |
| van Hemert et al. (2011) | 76.2 | 28 | NA | 5715 | GE-Composite indicators | None |
| van Hemert et al. (2011) | 76.2 | 15 | NA | 5715 | GE-Culture | None |
| van Hemert et al. (2011) | 76.2 | 30 | NA | 5715 | Other-Hofstede | Larger |
| Wang et al. (2016) | 77.1 | 6 continents | 84 | NA | Economy-GDP | None |
| Wang et al. (2016) | 77.1 | 6 continents | 84 | NA | Economy-Other | None |
| Weber et al. (2014) | 78.1 | 13 | NA | ≈31000 | GE-Education | Larger |
| Weber et al. (2014) | 78.1 | 13 | NA | ≈31000 | HD-Composite indicators | Larger |
| Weber et al. (2014) | 78.2 | 13 | NA | ≈31000 | GE-Education | Smaller |
| Weber et al. (2014) | 78.2 | 13 | NA | ≈31000 | HD-Composite indicators | Smaller |
| Weber et al. (2014) | 78.3 | 13 | NA | ≈31000 | GE-Education | Smaller |
| Weber et al. (2014) | 78.3 | 13 | NA | ≈31000 | HD-Composite indicators | None |
| Zentner & Mitura (2012) | 79.1 | 10 | NA | 3177 | GE-Composite indicators | Smaller |
| Zentner & Mitura (2012) | 79.2 | 31 | NA | 8953 | GE-Composite indicators | Smaller |
| Zhang et al. (2019) | 80.1 | 36 | NA | 2986 | GE-Composite indicators | None |
| Zhang et al. (2019) | 80.1 | 36 | NA | 2986 | GE-Composite indicators | None |
| Zhang et al. (2019) | 80.2 | 36 | NA | 2986 | GE-Composite indicators | None |
| Zhang et al. (2019) | 80.2 | 36 | NA | 2986 | GE-Composite indicators | None |
| Zhang et al. (2019) | 80.3 | 36 | NA | 2986 | GE-Composite indicators | None |
| Zhang et al. (2019) | 80.3 | 36 | NA | 2986 | GE-Composite indicators | None |
| Zuckerman et al. (2016) | 81.1 | 59 | 438 | ≈1170935 | Economy-Other | Larger |
| Zuckerman et al. (2016) | 81.1 | 59 | 438 | ≈1170935 | GE-Representation | Larger |
| Zuckerman et al. (2016) | 81.1 | 59 | 438 | ≈1170935 | HD-Labor | None |
| Zuckerman et al. (2016) | 81.1 | 59 | 438 | ≈1170935 | Other-Hofstede | Larger |
| Zuckerman et al. (2016) | 81.1 | 59 | 438 | ≈1170935 | Other-Hofstede | Larger |
| Zuckerman et al. (2016) | 81.1 | 59 | 438 | ≈1170935 | Other-Hofstede | Larger |
| Zuckerman et al. (2016) | 81.1 | 59 | 438 | ≈1170935 | Other-Hofstede | None |
| Zuckerman et al. (2016) | 81.1 | 59 | 438 | ≈1170935 | Other-Miscellaneous | Larger |
| Zuckerman et al. (2016) | 81.2 | 59 | 287 | ≈1170935 | Economy-Other | Larger |
| Zuckerman et al. (2016) | 81.2 | 59 | 287 | ≈1170935 | GE-Representation | None |
| Zuckerman et al. (2016) | 81.2 | 59 | 287 | ≈1170935 | HD-Labor | None |
| Zuckerman et al. (2016) | 81.2 | 59 | 287 | ≈1170935 | Other-Hofstede | Larger |
| Zuckerman et al. (2016) | 81.2 | 59 | 287 | ≈1170935 | Other-Hofstede | Larger |
| Zuckerman et al. (2016) | 81.2 | 59 | 287 | ≈1170935 | Other-Hofstede | None |
| Zuckerman et al. (2016) | 81.2 | 59 | 287 | ≈1170935 | Other-Hofstede | None |
| Zuckerman et al. (2016) | 81.2 | 59 | 287 | ≈1170935 | Other-Miscellaneous | None |
| Zuckerman et al. (2016) | 81.3 | 59 | 423 | ≈1170935 | Economy-Other | Larger |
| Zuckerman et al. (2016) | 81.3 | 59 | 423 | ≈1170935 | GE-Representation | Larger |
| Zuckerman et al. (2016) | 81.3 | 59 | 423 | ≈1170935 | HD-Labor | None |
| Zuckerman et al. (2016) | 81.3 | 59 | 423 | ≈1170935 | Other-Hofstede | Larger |
| Zuckerman et al. (2016) | 81.3 | 59 | 423 | ≈1170935 | Other-Hofstede | Larger |
| Zuckerman et al. (2016) | 81.3 | 59 | 423 | ≈1170935 | Other-Hofstede | None |
| Zuckerman et al. (2016) | 81.3 | 59 | 423 | ≈1170935 | Other-Hofstede | None |
| Zuckerman et al. (2016) | 81.3 | 59 | 423 | ≈1170935 | Other-Miscellaneous | Larger |

*Note.* GECL = Gender Equality in Respect for Civil Liberties. HGEI = Historical Gender Equality Index. RDI = Regional Development Index. LDI = Liberal Democracy Index. GE = Gender Equality. HD = Human Development.
